# Neuronal Modulators from the Coral-Associated Fungi *Aspergillus candidus*

**DOI:** 10.3390/md19050281

**Published:** 2021-05-19

**Authors:** Gao-Yang Peng, Tibor Kurtán, Attila Mándi, Jing He, Zheng-Yu Cao, Hua Tang, Shui-Chun Mao, Wen Zhang

**Affiliations:** 1 School of Pharmacy, Nanchang University, 461 Bayi Road, Nanchang 330006, China; penggaoyang42@163.com; 2 School of Medicine, Tongji University, 1239 Si-Ping Road, Shanghai 200092, China; 3 School of Pharmacy, Navy Medical University, 325 Guo-He Rd., Shanghai 200433, China; 4 Department of Organic Chemistry, University of Debrecen, POB 400, H-4002 Debrecen, Hungary; kurtan.tibor@science.unideb.hu (T.K.); mandi.attila@science.unideb.hu (A.M.); 5 State Key Laboratory of Natural Medicines, Department of TCM Pharmacology, School of Traditional Chinese Pharmacy, China Pharmaceutical University, 639 Long-Mian Ave., Nanjing 211198, China; 18796219566@163.com (J.H.); zycao1999@hotmail.com (Z.-Y.C.); 6 Institute of Translational Medicine, Shanghai University, 99 Shang-Da Road, Shanghai 200444, China; tanghua0309@126.com

**Keywords:** coral-associated fungi, secondary metabolites, *p*-terphenyl, indole-diterpene alkaloids, spontaneous Ca^2+^ oscillations

## Abstract

Three new *p*-terphenyl derivatives, named 4″-*O*-methyl-prenylterphenyllin B (**1**) and phenylcandilide A and B (**17** and **18**), and three new indole-diterpene alkaloids, asperindoles E–G (**22**-**24**), were isolated together with eighteen known analogues from the fungi *Aspergillus candidus* associated with the South China Sea gorgonian *Junceela fragillis*. The structures and absolute configurations of the new compounds were elucidated on the basis of spectroscopic analysis, and DFT/NMR and TDDFT/ECD calculations. In a primary cultured cortical neuronal network, the compounds **6**, **9**, **14**, **17**, **18** and **24** modulated spontaneous Ca^2+^ oscillations and 4-aminopyridine hyperexcited neuronal activity. A preliminary structure–activity relationship was discussed.

## 1. Introduction

Fungi of *Aspergillus candidus* is found to be wide-spread in soil [1] and marine environments [2,3,4], and also co-existing with various animals and plants [5,6,7,8]. The fungi are reported to produce prolific secondary metabolites, including *p*-terphenyls [9,10,11], terpenes [3], flavonoids [7,12,13], cyclopeptides [6], alkaloids [5], polyketones, and fatty acids [3]. These metabolites display a wide spectrum of biological activities, including cytotoxic [5,8], antibacterial [5,6], antifungal [3,8], antioxidant [6], and immunosuppressive activities [14].

As part of our continuing search for bioactive molecules from marine invertebrates and the associated fungi [15,16,17], a strain of *A. candidus* was isolated from the internal tissues of the gorgonian coral *Junceela fragillis*, collected from the Xisha area of the South China Sea. Chemical investigation of the fermentation extract of this fungus resulted in the isolation of three new *p*-terphenyl derivatives and three new indole-diterpene alkaloids, together with eighteen known analogues. Further, *p*-Terphenyls are regarded as the main metabolites of *A. candidus*. More than 230 analogues have been reported up to date, with the chemical diversity being attributed to the substituents on rings A and C [8,18,19]. The indole-diterpene alkaloids are a cluster of characteristic metabolites from this genus and were firstly reported from the titled fungi [20]. These metabolites are structurally constructed with an indole molecule and a saculatane diterpenoid. The biosynthetic process of both types of metabolites were conducted by investigating 6′-hydroxy-4,2′,3′,4″-tetra-methoxy-*p*-terphenyl [18,19,21] for *p*-terphenyls, and penitrem [22] and lolitrems [23,24] for the indole-diterpene alkaloids. The bioactivities and the intriguing ring-systems of these molecules also attracted attention for total synthesis [19,25,26]. Herein, we report the isolation, structure elucidation, and neuronal modulatory activity of these compounds.

## 2. Results

**Figure 1:**
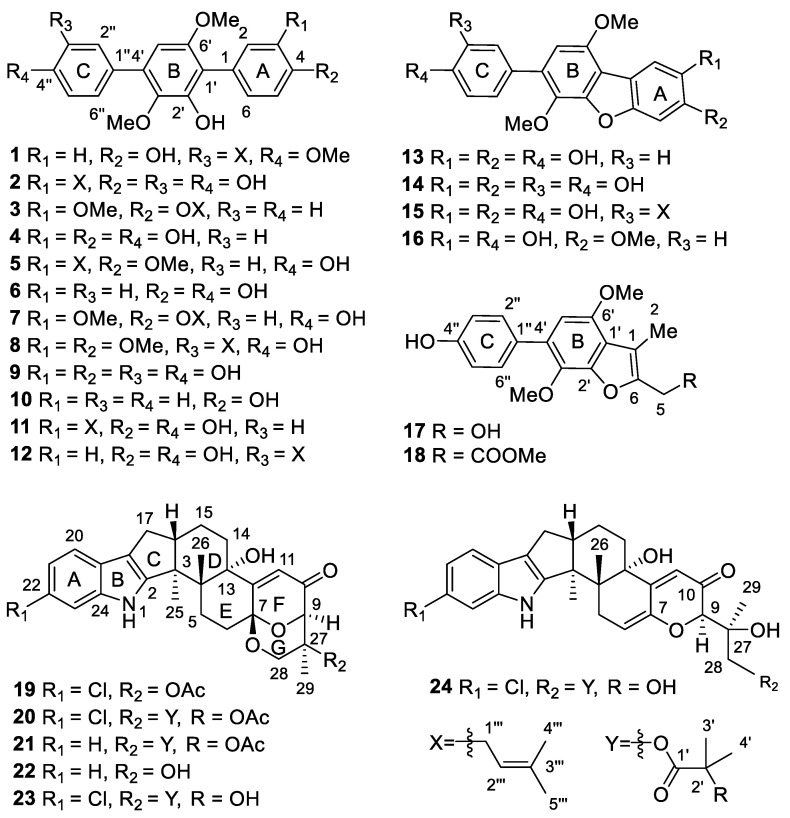
Structures of compounds **1**–**24**.

The *A. candidus* was cultivated on biomalt agar medium and then extracted with EtOAc. The EtOAc extract was subjected to the usual workup for isolation [15,16,27] to yield compounds **1**–**24** (Figure 1), including eighteen *p*-terphenyl derivatives and six indole-diterpene alkaloids. Based on the spectroscopic analysis and comparison with the reported data, the known compounds were determined as 3″-hydroxyl-prenylterphenyllin (**2**), 3-methoxy-4″-deoxyterprenin (**3**), 3-hydroxyterphenyllin (**4**) [28], 4-*O*-methylprenyl-terphenyllin (**5**) [9], terphenyllin (**6**) [12,28], 3-methoxyterprenin (**7**) [14], prenylterphenyllin J (**8**) [8], 3,3″-dihydroxy-6′-desmethyl terphenyllin (**9**) [29], deoxyterhenyllin (**10**) [30], prenylterphenyllin (**11**) [4], prenylterphenyllin B (**12**) [11], candidusins A (**13**) and B (**14**) [31], prenylcandidusin A (**15**) [11], 4-methyl-candidusin A (**16**) [32], and asperindoles A (**19**), C (**20**) and D (**21**) [20]. A complete NMR assignment is presented for the compounds **2** and **3** since there is no report for the NMR data of both the compounds. The assignments are fully supported by those of co-occurring prenylterphenyllin (**11**) [4] and 3-methoxyterprenin (**7**) [14], two analogues once reported from the soil-derived strains of the same species.

Further, 4″-*O*-methyl-prenylterphenyllin B (**1**) was obtained as a colorless amorphous solid. Its molecular formula was determined as C_26_H_28_O_5_ by HRESIMS, requiring 13 degrees of unsaturation. The IR spectrum displayed absorptions for hydroxy (3357 cm^−^^1^) and substituted benzol (1609, 1462, 834, and 815 cm^−^^1^) functionalities. The presence of benzol rings was supported by the strong UV absorptions at 276, 248, and 209 nm. The NMR spectra of **1** displayed resonances for twenty *sp*^2^ carbons and six *sp*^3^ carbons, taking into account the ten degrees of unsaturation. The remaining three degrees of unsaturation assigned to the ring system of this molecule were in agreement with that of the terphenyl framework. Its NMR data were almost identical to those of the co-isolated prenylterphenyllin B (**12**) [11], except for the appearance of an additional methoxy group (*δ*_H_ 3.89, 3H, s; *δ*_C_ 55.6, CH_3_). The methoxy group was assigned as 4″-OMe *via* its HMBC correlations to C-4″, and further confirmed by its NOE correlation with H-5″ (Figure S1). The structure of compound **1** was therefore determined as 4″-*O*-methyl-prenylterphenyllin B.

**Figure 2:**
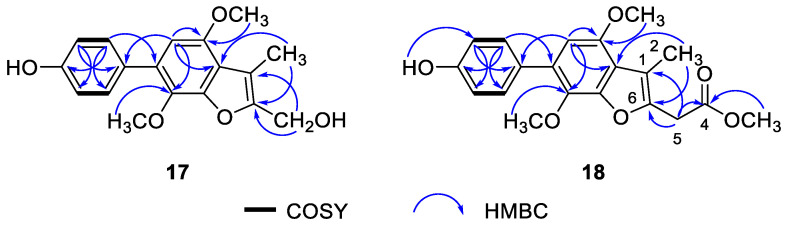
Selected COSY and HMBC correlations of compounds **17** and **18**.

Phenylcandilide A (**17**), obtained as a yellow amorphous solid, had a molecular formula of C_18_H_18_O_5_ as deduced from the HRESIMS, indicating 10 degrees of unsaturation. The NMR data of 17 showed similarity to those of 4-methyl-candidusin A (**16**) [32], regarding the signals for the phenol ring C and benzofuran ring B. The C-2 to C-5 diene fragment of the phenol ring A in **16**, however, was degraded to a methyl and a hydroxymethyl groups in **17**. The distinct HMBC correlations from H_3_-2 to C-1′ and C-6, and H_2_-5 to C-6 and C-1 confirmed the location of 1-Me and 6-CH_2_OH (Figure 2). The structure of **17** was determined and nominated as phenylcandilide A.

Phenylcandilide B (**18**) was obtained as a yellow amorphous solid. Its molecular formula was established as C_20_H_20_O_6_ on the basis of the HRESIMS. Comparison of its NMR data (Table 1) with those of **17** revealed a similarity in the structures. A difference was recognized for the signals of the hydroxymethyl group in **17** being replaced by a methyl acetate subunit in **18**, confirmed by the IR at 1740 cm^–1^. The location of the methyl acetate subunit was indicated by the diagnostic HMBC correlations from H_2_-5 to C-4, C-1 and C-6, and 4-OMe to C-4 (Figure 2). The structure of compound **18** was thus determined and nominated as phenylcandilide B.

**Figure 3:**
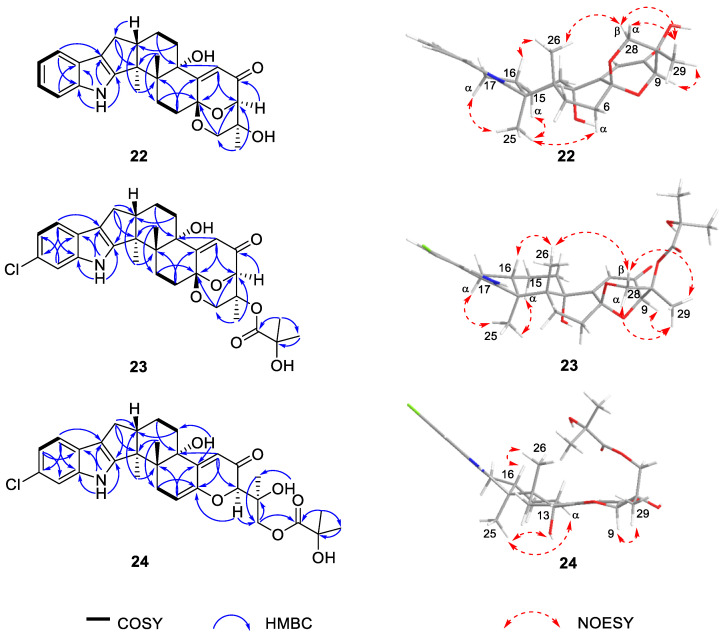
Selected COSY, HMBC, and NOESY correlations of compounds **22**–**24**.

**Figure 4:**
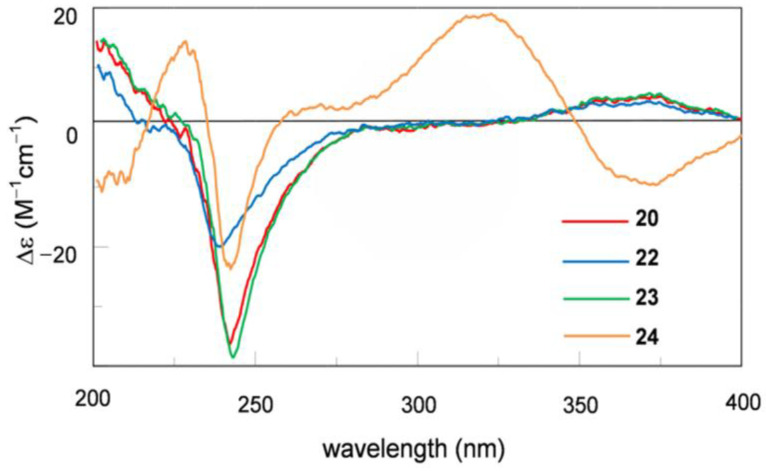
Experimental ECD data of compounds **20** and **22–24** in MeCN.

Asperindole E (**22**) was obtained as an optically active, white powder. Its molecular formula was established as C_27_H_31_NO_5_ by HRESIMS, implying 13 degrees of unsaturation. The IR spectrum of **22** displayed absorptions for hydroxy (3360 cm^–1^) and substituted benzol (1632, 1468, 800, and 742 cm^−^^1^) functionalities. The presence of an *α*,*β*-unsaturated ketone moiety was suggested by the characteristic IR absorption at 1658 cm^−^^1^. The strong UV absorptions at 279, 268, 230 and 210 nm were in agreement with the presence of a benzol ring or an *α*,*β*-unsaturated ketone moiety in the structure. The NMR spectra (Table 2) demonstrated a great similarity to the known metabolites of asperindoles A–D, previously obtained from an ascidian-derived fungus *Aspergillus* sp. [20], suggesting the same indole-diterpene framework for these molecules. Signals for the acetyl group in asperindole B were not observed for **22**. The structure of **22** was suggested to be the deacetyl analogues of asperindole B, which was fully confirmed by 2D NMR experiments, particularly HMBC and NOESY (Figure 3). The absolute configurations of **22** were the same as that of asperindoles A–D on the basis of the similar ECD spectra (Figure 4). The structure of compound **22** was thus determined as asperindole E.

Asperindole F (**23**), obtained as an optically active, white powder, has a molecular formula of C_31_H_36_ClNO_7_ as determined by HRESIMS. The presence of a chlorine atom in the molecule was indicated by the isotopic peaks at *m*/*z* 568/570 [M − H]^−^ with a ratio of 3:1. The NMR spectra of **23** (Table 2) resembled those of asperindole C (**20**) [20] (Table S1), except for the acetyl group which is absent, showing the same difference pattern as that between asperindole E (**22**) and B. Compound **23** is the deacetylated derivative of asperindole C, and was named as asperindole F. This assignment was further confirmed by NMR and ECD experiments (Figure 3 and Figure 4).

**Figure 5:**
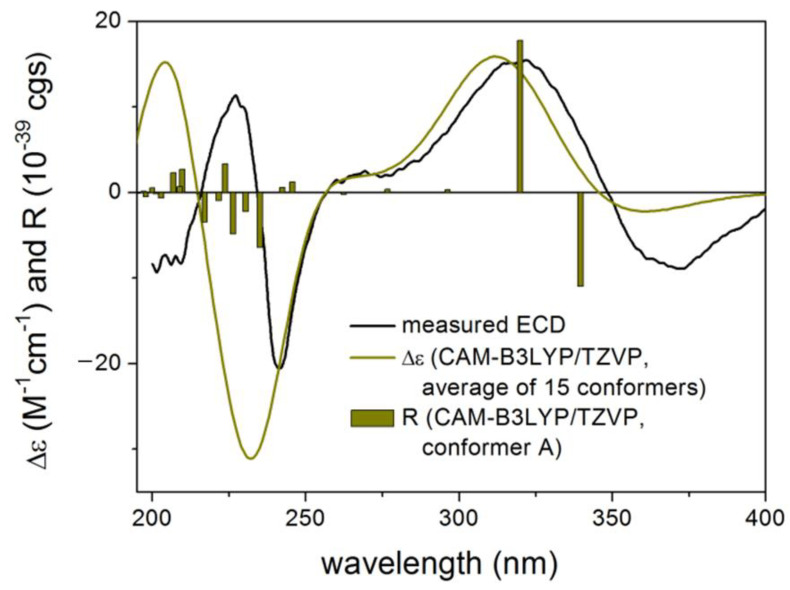
Experimental ECD spectrum of **24** in MeCN compared with the Boltzmann-weighted CAM-B3LYP/TZVP PCM/MeCN ECD spectrum of (3*S*,4*R*,9*R*,13*S*,16*S*,27*S*)-**24**. Level of optimization: ωB97X/TZVP PCM/MeCN. Bars represent the rotatory strength values of the lowest-energy conformer.

**Figure 6:**
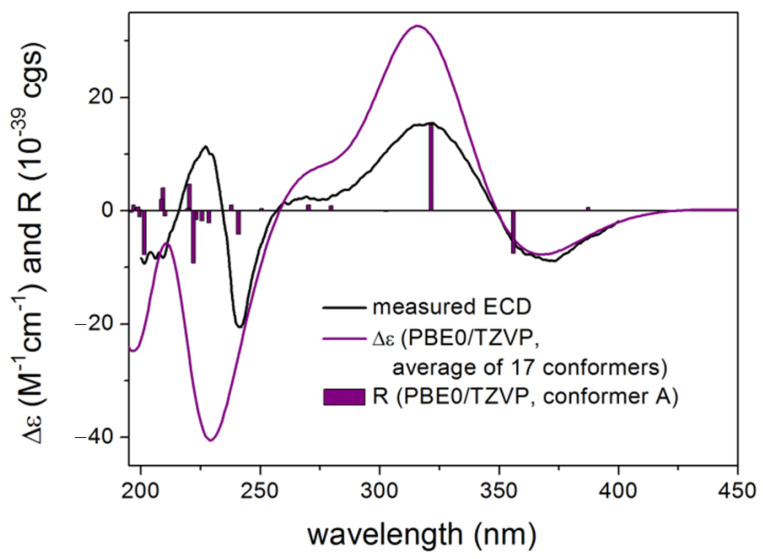
Experimental ECD spectrum of **24** in MeCN compared with the Boltzmann-weighted CAM-B3LYP/TZVP PCM/MeCN ECD spectrum of (3*S*,4*R*,9*S*,13*S*,16*S*,27*S*)-**24**. Level of optimization: ωB97X/TZVP PCM/MeCN. Bars represent the rotatory strength values of the lowest-energy conformer.

Asperindole G (**24**) was obtained as an optically active, white powder. Its HRESIMS gave the same molecular formula as that of asperindole F (**23**). As expected, the NMR data of **24** (Table 2) showed similarity to those of **23**. However, two *sp*^3^ carbon atoms (*δ* 94.0, C; *δ* 30.7, CH_2_) in **23** were replaced by two *sp*^2^ carbon atoms (*δ* 145.0, C; *δ* 111.5, CH) in **24**, suggesting a cleavage of the ether bridge of ring G in **23** to form a C-6 to C-7 double bond, and a 28-hydroxymethyl group in **24**. The assignment for the C-6 to C-7 double bond was confirmed by the HMBC correlations of H-6 with C-4 and C-12, and H-9 with C-7. In addition, the *α*-hydroxyisobutyrate moiety was attached to the C-28 methylene group of the side-chain instead of the C-27 of the bridged 1,3-dioxane ring (as those in **19–23**), as confirmed by the diagnostic HMBC correlations between H_2_-28 and the ester carbonyl carbon (*δ* 175.5, C). Compound **24** was expected to have the same stereochemistry as that of **19–23** due to their obvious correlations in biogenetic origin, even though the ECD spectrum of **24** was significantly different from those of **20–23**. However, a very weak NOE correlation was observed between H-9 and H_3_-26 in the NOESY spectrum of **24**. This suggested that an epimerization might occur at C-9, which is adjacent to the C-10 ketone group. In order to determine the absolute configuration of C-9, TDDFT-ECD [33] and DFT-NMR calculations [34] were performed on the (3*S*,4*R*,9*R*,13*S*,16*S*,27*S*) and (3*S*,4*R*,9*S*,13*S*,16*S*,27*S*) epimers. Ring F, containing an *α*,*β*-unsaturated carbonyl chromophore, was expected to have a major impact on the high-wavelength ECD transitions, and thus a large difference was expected between the ECD spectra of the two epimers. The initial Merck molecular force field (MMFF) conformational search resulted in 353 and 156 conformers in a 21 kJ/mol energy window for (3*S*,4*R*,9*R*,13*S*,16*S*,27*S*)-**24** and (3*S*,4*R*,9*S*,13*S*,16*S*,27*S*)-**24**, respectively. The re-optimization of these conformers yielded 15 and 17 low-energy conformers over the 1% Boltzmann distribution at the ωB97X/TZVP [35] PCM/MeCN level (Figures S83 and S84). Unexpectedly, the Boltzmann-weighted ECD spectra of both epimers computed at various levels reproduced well the major transitions of the experimental ECD spectrum, indicating that the contribution of ring E is dominant. However, the (9*R*) epimer could reproduce the 225 nm positive Cotton effect (CE) with a blue shift (Figure 5), while the (9*S*) epimer had only a negative computed CE below 250 nm (Figure 6). This small difference suggested that **24** had (9*R*) absolute configuration, and the difference in the experimental ECD spectra of **20–23** and **24** derives from the different chromophore systems and different planar structures. It is well-documented that even small structural changes can result in markedly different or mirror-image ECD spectra for homochiral derivatives by changing the preferred conformation or electronic properties of the molecule [36]. The (9*R*) and (9*S*) epimers were further distinguished by ^13^C NMR DFT calculations, which has been proven an efficient method to distinguish diastereomeric natural products [17,34]. For the NMR calculations, the above MMFF conformers were re-optimized at the B3LYP/6-31 + G(d,p) level yielding 16 and 14 low-energy structures above the 1% Boltzmann distribution, respectively. Despite the DMSO solvent and the presence of the halogen, both causing larger deviations in the computed data, the calculated ^13^C NMR shifts of the (9*R*) epimer had a slightly smaller MAE average value than those of the (9*S*) epimer, and the DP4+ statistical analysis [35,37] resulted in an 83.75% confidence for the (9*R*) epimer (Table S2). Since both the computed ECD and NMR data suggested (9*R*) configuration, the H-9 and H_3_-26 NOE cross-peak must be an artefact, and the absolute configuration of **24** was determined to be (3*S*,4*R*,9*R*,13*S*,16*S*,27*S*). The interatomic distance of H-9 and H_3_-26 is 3.7 Å in the (9*S*) epimer and it is above 5.0 Å in the (9*R*) epimer.

All the isolated compounds were evaluated for their neuronal modulatory activities by testing their effect on spontaneous Ca^2+^ oscillations (SCOs), and the seizurogenic agent 4-aminopyridine (4-AP) induced hyperexcitation in primary cultured neocortical neurons (Table 3). SCOs play a crucial role in mediating neuron development, and are closely associated with neuronal excitable and inhibitory neuronal transmission [38,39,40]. The compounds with modulatory activity on SCOs may have potential in drug candidates for treating neurological diseases such as epilepsy, pain and depression [41].

In the present study, we found that four compounds **6**, **17**, **18** and **24** inhibited SCO activity, and 4-AP induced hyperexcitability by decreasing the SCO amplitude and frequency in the primary cultured cortical neuronal network. However, compounds **9** and **14** produced a more complicated Ca^2+^ response. Their concentration dependently increased the SCO frequency with the concurrent suppression of the SCO amplitude at concentrations below 10 μM and 3 μM, respectively, and transiently increased the intracellular Ca^2+^ concentration which recovered to basal level within 5 min at concentrations of 30 μM and 10 μM, respectively (Table 3, and Figures S85–S90). For the cluster of *p*-terphenyl derivatives, all the active compounds have hydroxyls for both R_2_ and R_4_ and those with hydrogens for both R_1_ and R_3_ displayed the strongest activity. Substitution for one of the R_2_/R_4_ pair of hydroxyls or one of the R_1_/R_3_ pair of hydrogens will decrease the activity. Interestingly, the degradation of ring A to a hydroxymethyl group may lead to an increase in activity. For the cluster of indole-diterpene alkaloids, ring cleavage on the ether bridge of ring G seems critical for the activity since all those compounds that have ring G are not active.

## 3. Experimental Section

### 3.1. General Experimental Procedures

Optical rotations were determined with a Rudolph Autopol VI polarimeter. UV spectra were recorded on a Shimadzu UV-2700 spectrophotometer. IR spectra were recorded on a Bruker TENSOR II spectrophotometer. The ECD spectra were measured with a Jasco-715 spectropolarimeter. NMR spectra were recorded on Bruker DRX-600 and DRX-500 spectrometers, and the signals of residual CHCl_3_ (*δ*_H_ 7.26 ppm; *δ*_C_ 77.0 ppm), DMSO (*δ*_H_ 2.50 ppm; *δ*_C_ 39.6 ppm) and CH_3_OH (*δ*_H_ 3.31 ppm; *δ*_C_ 49.0 ppm) were used as references for chemical shifts. The HR-ESI-MS data were recorded on an Agilent 1290-6545 UHPLC-Q-TOF-MS spectrometer. Semipreparative HPLC was performed using an Agilent Technology 1100 system with an ODS column (YMC Pack ODS-A, 10 × 250 mm, 5 μM). Column chromatography (CC) was performed with Sephadex LH-20 gel (SE-751 84 Uppsala, GE Healthcare Bio-Sciences AB) and silica gel (200–300 mesh, 400–600 mesh; Yantai, China), respectively. The thin-layer chromatography (TLC) experiments were conducted with silica gel plates (HSGF-254, Yantai, China), and detected by heating after spraying with anisaldehyde sulfuric acid reagent.

### 3.2. Fungal Material

The fungal strain SG-8-⑤ was isolated from the internal tissues of the gorgonian coral *J. fragillis*, which was collected from the Xisha area of the South China Sea, and identified as *A. candidus* by 18sRNA sequence (GenBank accession number AB008396.1). The fungus was deposited in Tongji University, Shanghai, China.

### 3.3. Culture, Extraction and Isolation

The fungal strain *A. candidus* was cultivated on 20.0 L of 5% w/v biomalt (Villa Natura, Germany) solid agar medium (20.0 g/L biomalt, 15.0 g/L agar, 800 mL/L artificial seawater) at room temperature for 28 days. The fungal mycelia with the medium were extracted with EtOAc (4.0 L × 5) under ultrasonic conditions. The EtOAc extract was concentrated under vacuum (13.6 g) and was separated into thirteen fractions (Fr.1–13) by silica gel CC, eluting with a gradient CH_2_Cl_2_/MeOH (*v/v* 100:0 to 4:1). Fraction 2 (52.7 mg) was subjected to a Sephadex LH-20 CC (CH_2_Cl_2_/MeOH 2:1) and then purified by HPLC (MeOH/H_2_O 89:11, 1.5 mL/min) to give **3** (1.3 mg, *t*_R_ 18 min). Fraction 5 (29.9 mg) was subjected to a Sephadex LH-20 CC (CH_2_Cl_2_/MeOH 2:1) and HPLC (MeOH/H_2_O 80:20, 2.0 mL/min) to afford **5** (3.1 mg, *t*_R_ 24 min). Fraction 6 (644.9 mg) was subjected to a Sephadex LH-20 CC (CH_2_Cl_2_/MeOH 2:1) to give eight subfractions (Fr.6a to Fr.6h). The Fr.6e (215.8 mg) was separated by silica gel CC using a gradient petroleum (PE) in acetone (39:1 to 6:4), then split by HPLC to yield **7** (1.2 mg, MeOH/H_2_O 79:21, 1.5 mL/min, *t*_R_ 18 min), **8** (0.9 mg, MeOH/H_2_O 79:21, 1.5 mL/min, *t*_R_ 21 min), **1** (1.2 mg, MeOH/H_2_O 80:20, 2.0 mL/min, *t*_R_ 33 min), **19** (1.8 mg, MeOH/H_2_O 80:20, 2.0 mL/min, *t*_R_ 50 min), **20** (2.3 mg, MeOH/H_2_O 80:20, 2.0 mL/min, *t*_R_ 63 min), **21** (1.1 mg, MeOH/H_2_O 80:20, 2.0 mL/min, *t*_R_ 36 min). Fr.6f (54.7 mg) was separated by silica gel CC (PE-EtOAc 9:1 to 1:1) and purified by HPLC (MeOH/H_2_O 80:20, 2.0 mL/min) to afford **23** (1.2 mg, *t*_R_ 44 min) and **24** (0.7 mg, *t*_R_ 25 min). Fr.6g (18.7 mg) was purified by HPLC (MeOH/H_2_O 70:30, 2.0 mL/min) to afford **10** (0.8 mg, *t*_R_ 23 min) and **22** (0.9 mg, *t*_R_ 64 min). Fr.6h (52.4 mg) was purified by HPLC (MeOH/H_2_O 65:35, 2.0 mL/min) to yield **16** (1.6 mg, *t*_R_ 30 min), **11** (1.5 mg, *t*_R_ 48 min), and **12** (1.0 mg, *t*_R_ 51 min). Fraction 8 (164.7 mg) was subjected to a Sephadex LH-20 CC (CH_2_Cl_2_/MeOH 2:1) to yield thirteen subfractions (Fr.8a to Fr.8m). Fr.8h (19.8 mg) was further purified by HPLC (MeOH/H_2_O 70:30, 1.5 mL/min) to afford **6** (3.6 mg, *t*_R_ 13 min), **2** (0.5 mg, *t*_R_ 20 min), and **15** (3.1 mg, *t*_R_ 41 min). Fr.8j (33.4 mg) was purified by HPLC (MeOH/H_2_O 65:35, 2.0 mL/min) to afford **13** (13.9 mg, *t*_R_ 18 min). Fraction 9 (364.2 mg) was subjected to a Sephadex LH-20 CC (CH_2_Cl_2_/MeOH 2:1) to yield **4** (50.0 mg). Fraction 10 (649.6 mg) was subjected to a Sephadex LH-20 CC (CH_2_Cl_2_/MeOH 2:1) to furnish fourteen subfractions (Fr.10a to Fr.10n) and pure compound **14** (11.6 mg). Fr.10g (29.3 mg) was further purified by HPLC (MeOH/H_2_O 70:30, 2.0 mL/min) to afford **18** (2.0 mg, *t*_R_ 27.5 min). Fr.10i (84.5 mg) was separated by silica gel CC with the eluent of gradient petroleum ether-ethyl acetate from 9:1 to 1:1, followed by the purification of HPLC (MeOH/H_2_O 58:42, 2.0 mL/min) to yield **17** (2.5 mg, *t*_R_ 42 min). Fraction 11 (201.0 mg) was applied on a Sephadex LH-20 CC (CH_2_Cl_2_/MeOH 2:1) and purified by HPLC (MeOH/H_2_O 55:45, 2.0 mL/min) to afford **9** (13.0 mg, *t*_R_ 11 min).

The 4″-*O*-methyl-prenylterphenyllin B (**1**): colorless, amorphous solid; UV (MeCN) *λ*_max_ (log *ε*) 276 (3.39), 248 (3.12), 209 (3.64) nm; IR (film) *ν*_max_ 3357, 2922, 2852, 1659, 1609, 1462, 1246, 1073, 1029, 834, 815 cm^−1^; ^1^H and ^13^C NMR data see Table 1; HRESIMS *m/z* 419.1870 [M − H]^−^ (calcd for C_26_H_27_O_5_, 419.1864).

The 3″-hydroxyl-prenylterphenyllin (**2**): colorless, amorphous solid; UV (MeCN) *λ*_max_ (log *ε*) 277 (3.28), 250 (3.13), 210 (3.65) nm; IR (film) *ν*_max_ 3346, 2922, 2853, 1665, 1607, 1461, 1260, 1093, 799, 722 cm^−1^; ^1^H NMR (CD_3_OD, 500 MHz): *δ*_H_ 7.11 (1H, d, *J* = 2.2 Hz, H-2″), 7.05 (1H, d, *J* = 2.2 Hz, H-2), 7.01 (1H, dd, *J* = 8.2, 2.2 Hz, H-6), 6.96 (1H, dd, *J* = 8.2, 2.2 Hz, H-6″), 6.83 (1H, d, *J* = 8.2 Hz, H-5″), 6.77 (1H, d, *J* = 8.2 Hz, H-5), 6.42 (1H, s, H-5′), 5.37 (1H, m, H-2‴), 3.67 (3H, s, 6′-OMe), 3.41 (3H, s, 3′-OMe), 3.32 (2H, m, H-1‴), 1.73 (3H, s, H-5‴), 1.72 (3H, s, H-4‴); ^13^C NMR (CD_3_OD, 125 MHz): *δ*_C_ 154.8 (C, C-4), 149.1 (C, C-2′), 146.1 (C, C-3″), 145.9 (C, C-4″), 140.8 (C, C-3′), 134.2 (C, C-4′), 133.2 (CH, C-2), 132.7 (C, C-3‴), 131.7 (C, C-1″), 130.3 (CH, C-6), 128.2 (C, C-3), 126.3 (C, C-1), 124.2 (CH, C-2‴), 121.5 (CH, C-6″), 119.0 (C, C-1′), 117.2 (CH, C-2″), 116.2 (CH, C-5″), 115.1 (CH, C-5), 105.0 (CH, C-5′), 60.8 (CH_3_, 3′-OMe), 56.5 (CH_3_, 6′-OMe), 29.3 (CH_2_, C-1‴), 26.0 (CH_3_, C-5‴), 17.9 (CH_3_, C-4‴); HRESIMS *m*/*z* 423.1787 [M + H]^+^ (calcd for C_25_H_27_O_6_, 423.1802).

The 3-methoxy-4″-deoxyterprenin (**3**): colorless, amorphous solid; UV (MeCN) *λ*_max_ (log *ε*) 275 (3.27), 250 (3.14), 202 (3.68); IR (film) *ν*_max_ 3356, 2924, 2853, 1664, 1601, 1461, 1266, 1075, 829, 771 cm^−1^; ^1^H NMR (CDCl_3_, 500 MHz): *δ*_H_ 7.64 (2H, dd, *J* = 7.5, 1.5 Hz, H-6″), 7.46 (2H, td, *J* = 7.5, 1.5 Hz, H-5″), 7.39 (1H, tt, *J* = 7.5, 1.5 Hz, H-4″), 7.02 (1H, dd, *J* = 8.7, 2.0 Hz, H-6), 7.02 (1H, d, *J* = 2 Hz, H-2), 6.98 (1H, d, *J* = 8.7 Hz, H-5), 6.50 (1H, s, H-5′), 5.93 (1H, s, 2′-OH), 5.57 (1H, m, H-2‴), 4.64 (2H, d, *J* = 7 Hz, H-1‴), 3.88 (3H, s, 3-OMe), 3.76 (3H, s, 6′-OMe), 3.46 (3H, s, 3′-OMe), 1.79 (3H, s, H-5‴), 1.75 (3H, s, H-4‴); ^13^C NMR (CDCl_3_, 125 MHz): *δ*_C_ 153.6 (C, C-6′), 149.1 (C, C-3), 147.8 (C, C-4), 147.4 (C, C-2′), 139.0 (C, C-3′), 138.2 (C, C-1″), 137.6 (C, C-3‴), 133.0 (C, C-4′), 128.9 (CH, C-2″, 6″), 128.6 (CH, C-3″, 5″), 127.7 (CH, C-4″), 125.4 (C, C-1), 123.0 (CH, C-6), 120.3 (CH, C-2‴), 117.0 (C, C-1′), 114.4 (CH, C-2), 112.6 (CH, C-5), 104.2 (CH, C-5′), 65.8 (CH_2_, C-1‴), 61.1 (CH_3_, 3′-OMe), 56.2 (CH_3_, 6′-OMe), 56.1 (CH_3_, 3-OMe), 26.0 (CH_3_, C-5‴), 18.4 (CH_3_, C-4‴); HRESIMS *m*/*z* 421.2027 [M + H]^+^ (calcd for C_26_H_29_O_5_, 421.2010).

Phenylcandilide A (**17**): yellow, amorphous solid; UV (MeCN) *λ*_max_ (log *ε*) 276 (3.41), 256 (3.16), 242 (3.45), 227 (3.33), 209 (3.50), 203 (3.49) nm; IR (film) *ν*_max_ 3359, 2922, 2852, 1658, 1469, 1266, 1206, 1091, 831, 738 cm^−1^; ^1^H and ^13^C NMR data see Table 1; HRESIMS *m*/*z* 337.1055 [M + Na]^+^ (calcd for C_18_H_18_NaO_5_, 337.1046).

Phenylcandilide B (**18**): yellow, amorphous solid; UV (MeCN) *λ*_max_ (log *ε*) 275 (3.58), 255 (3.37), 240 (3.61), 227 (3.54), 212 (3.64) nm; IR (film) *ν*_max_ 3354, 2924, 2853, 1740, 1660, 1497, 1267, 1213, 1172, 1104, 830, 671 cm^−1^; ^1^H and ^13^C NMR data see Table 1; HRESIMS *m*/*z* 357.1349 [M + H]^+^ (calcd for C_20_H_21_O_6_, 357.1333).

Asperindole E (**22**): white powder; ${\left[\alpha \right]}_{D}^{20}$ +50.00 (*c* 0.03, CHCl_3_); UV (MeCN) λ_max_ (log *ε*) 279 (2.78), 268 (2.75), 230 (3.45), 210 (3.24) nm; ECD (0.045 mM, MeCN) *λ*_max_ (Δ*ε*) 238 (−20.91) nm; IR (film) *ν*_max_ 3360, 2920, 2851, 1720, 1658, 1632, 1468, 1260, 1016, 800, 742 cm^−1^; ^1^H and ^13^C NMR data see Table 3; HRESIMS *m*/*z* 472.2086 [M + Na]^+^ (calcd for C_27_H_31_NO_5_Na, 472.2094).

Asperindole F (**23**): white powder; ${\left[\alpha \right]}_{D}^{20}$ +40.56 (*c* 0.03, CHCl_3_); UV (MeCN) *λ*_max_ (log *ε*) 285 (2.89), 267 (2.76), 236 (3.63), 209 (3.23) nm; ECD (0.035 mM, MeCN) λ_max_ (Δ*ε*) 242 (−39.92) nm; IR (film) *ν*_max_ 3358, 2920, 2851, 1730, 1659, 1633, 1467, 1260, 1013, 799, 704 cm^−1^; ^1^H and ^13^C NMR data see Table 3; HRESIMS *m*/*z* 568.2120 [M − H]^−^ (calcd for C_31_H_35_ClNO_7_, 568.2108).

Asperindole G (**24**): white powder; ${\left[\alpha \right]}_{D}^{20}$ +31.11 (*c* 0.03, CHCl_3_); UV (MeCN) λ_max_ (log *ε*) 303 (2.92), 293 (2.90), 286 (2.91), 270 (2.85), 236 (3.56), 211 (3.33) nm; ECD (0.035 mM, MeCN), *λ*_max_ (Δ*ε*) 373 (−8.91), 362 (−7.78), 322 (+15.46), 269 (+2.49), 241 (−20.56), 227 (+11.34) nm; IR (film) *ν*_max_ 3349, 2920, 2851, 1730, 1659, 1462, 1260, 1018, 798 cm^−1^; ^1^H and ^13^C NMR data see Table 3; HRESIMS *m*/*z* 592.2068 [M + Na]^+^ (calcd for C_31_H_36_ClNO_7_Na, 592.2073).

### 3.4. Neuronal Modulatory Activity Assay In Vitro

The neuronal modulatory activities of **1**–**24** were evaluated by testing the effect on spontaneous Ca^2+^ oscillations (SCOs) and seizurogenic agent 4-aminopyridine (4-AP)-induced hyperactive SCOs frequency and amplitude in primary cultured neocortical neurons as described previously [16,38]. Neocortical neurons at 9 days in vitro (DIV) were used to investigate the influence of tested compounds on intracellular Ca^2+^ concentration ([Ca^2+^]_i_). Briefly, the neurons were loaded with Fluo-4 for 1 h at 37 °C in Locke’s buffer. After recording the baseline spontaneous Ca^2+^ oscillations for 5 min, different concentrations of compounds were added to the corresponding well, and the [Ca^2+^]_i_ was monitored for 15 min using FLIPR^tetra®^. To test anti-epileptic potential of the meroterpenoids, 4-AP (10 μM) was added and the monitoring for [Ca^2+^]_i_ was continued for an additional 10 min. The presented data were values of F/F_0_, where F is the fluorescence intensity at any time point whereas F_0_ is the basal fluorescence. An event with Δ F/F_0_ over 0.1 unit was considered to be an SCO. The frequency and amplitude of SCOs were quantified using Origin software (V7.0) from a time period of 5 min after first or second addition of compound or vehicle (0.1% DMSO).

### 3.5. Computational Section

Mixed torsional/low-frequency mode conformational searches were carried out by means of the Macromodel 10.8.011 software by using the Merck molecular force field (MMFF) with an implicit solvent model for CHCl_3_ [42]. Geometry re-optimizations were carried out at the B3LYP/6-31 + G(d,p) level in vacuo and the ωB97X/TZVP level with the PCM solvent model for MeCN. TDDFT-ECD calculations were run with various functionals (B3LYP, BH&HLYP, CAMB3LYP, and PBE0) and the TZVP basis was set as implemented in the Gaussian 09 package, with the same or no solvent model as in the preceding DFT optimization step [43]. ECD spectra were generated as sums of Gaussians with 3000 and 2700 cm^−1^ widths at half-height, using dipole-velocity-computed rotational strength values [44]. NMR calculations were performed at the mPW1PW91/6-311 + G(2d,p) level [45]. Computed NMR shift data were corrected with I = 185.2853 and S = −1.0306 [46]. Boltzmann distributions were estimated from the B3LYP and ωB97X energies. The MOLEKEL software package was used for visualization of the results [47].

## 4. Conclusions

From the fungi *A. candidus*, associated with the South China Sea gorgonian *Junceela fragillis*, twenty-four metabolites having *p*-terphenyl and indole-diterpene frameworks were obtained with their structures and absolute configurations being elucidated on the basis of spectroscopic analysis and computational calculations. It was found that small structural changes can result in a markedly different ECD spectra, and ^13^C NMR DFT calculations are an efficient method to distinguish diastereomeric natural products. Six compounds could modulate SCOs and 4-aminopyridine hyperexcited neuronal activity in the *in vitro* biotest. A preliminary structure–activity relationship was discussed, which may give a reference for further investigation or chemical optimization of SCO modulators.

## Figures and Tables

**Table 1:** ^1^H and ^13^C NMR data for **1**, **17** and **18**.

Position	1 (in CDCl_3_)		17 (in DMSO)		18 (in DMSO)	
	*δ*_H_^a^ (*J* in Hz)	*δ*_C_^b^, Type	*δ*_H_^c^ (*J* in Hz)	*δ*_C_^b^, Type	*δ*_H_^a^ (*J* in Hz)	*δ*_C_^b^, Type
1		125.7, C		111.7, C		112.6, C
2	7.36, d (8.5)	132.2, CH	2.30, s	9.5, CH_3_	2.25, s	9.4, CH_3_
3	6.92, d (8.5)	115.2, CH				
4		154.8, C				169.4, C
5	6.92, d (8.5)	115.2, CH	4.51, d (5.7)	53.5, CH_2_	3.93, s	31.6, CH_2_
6	7.36, d (8.5)	132.2, CH		152.2, C		145.8, C
1′		116.1, C		119.1, C		119.0, C
2′		147.3, C		146.9, C		146.9, C
3′		138.9, C		135.9, C		135.7, C
4′		132.8, C		129.0, C		128.9, C
5′	6.46, s	104.0, CH	6.57, s	105.0, CH	6.58, s	105.2, CH
6′		153.5, C		149.6, C		149.3, C
1″		130.3, C		128.6, C		128.6, C
2″	7.44, d (2.5)	130.0, CH	7.38, d (8.7)	130.3, CH	7.37, d (8.5)	130.3, CH
3″		130.2, C	6.83, d (8.7)	115.0, CH	6.83, d (8.5)	115.0, CH
4″		157.0, C		156.5, C		156.5, C
5″	6.93, d (8.7)	110.3, CH	6.83, d (8.7)	115.0, CH	6.83, d (8.5)	115.0, CH
6″	7.45, dd (8.7, 2.5)	127.2, CH	7.38, d (8.7)	130.3, CH	7.37, d (8.5)	130.3, CH
1‴	3.38, d (7.5)	28.7, CH_2_				
2‴	5.36, t (7.5)	122.5, CH				
3‴		132.8, C				
4‴	1.73, s	18.0, CH_3_				
5‴	1.75, s	26.0, CH_3_				
4-OMe					3.66, s	52.1, CH_3_
3′-OMe	3.45, s	60.8, CH_3_	3.71, s	60.4, CH_3_	3.68, s	60.3, CH_3_
6′-OMe	3.74, s	56.1, CH_3_	3.85, s	55.7, CH_3_	3.85, s	55.7, CH_3_
4″-OMe	3.89, s	55.6, CH_3_				
5-OH			5.28, t (5.7)			
2′-OH	5.94, s					
4″-OH			9.51, s		9.48, s	

^a^ 500 MHz. ^b^ 125 MHz. ^c^ 600 MHz.

**Table 2:** ^1^H and ^13^C NMR data for **22–24**.

Position	22 (in CDCl_3_)		23 (in CDCl_3_)		24 (in DMSO)	
	*δ*_H_^a^ (*J* in Hz)	*δ*_C_^b^, Type	*δ*_H_^c^ (*J* in Hz)	*δ*_C_^b^, Type	*δ*_H_^a^ (*J* in Hz)	*δ*_C_^b^, Type
1-NH	7.70, s		7.72, s		10.96, s	
2		152.0, C		152.8, C		153.9, C
3		52.0, C		52.1, C		50.4, C
4		39.4, C		39.4, C		42.7, C
5α	1.87, dd (11.5, 9.2)	27.3, CH_2_	1.84, ov ^d^	27.2, CH_2_	2.37, dd (17.0, 6.0)	30.6, CH_2_
5β	2.65, dd (11.5, 9.5)		2.60, ov ^d^		3.02, d (17.0)	
6α	2.23, dd (13.0, 9.2)	31.1, CH_2_	2.12, dd (13.2, 9.0)	30.7, CH_2_	5.69, m	111.5, CH
6β	2.66, dd (13.0, 9.5)		2.61, ov ^d^			
7		94.5, C		94.0, C		145.0, C
9	4.05, d (2.0)	84.0, CH	4.81, d (2.4)	79.2, CH	4.25, d (1.5)	82.1, CH
10		196.5, C		196.0, C		194.7, C
11	6.24, s	120.7, CH	6.22, s	120.4, CH	5.84, s	116.1, CH
12		158.9, C		159.8, C		154.5, C
13		78.9, C		78.8, C		73.8, C
14α	2.00, ov^d^	33.9, CH_2_	2.00, ov ^d^	33.8, CH_2_	1.93, ov^d^	31.7, CH_2_
14β	1.97, ov^d^		1.96, dd (13.2, 4.8)		1.83, ov^d^	
15α	2.08, dd (13.0, 4.0)	21.3, CH_2_	2.09, ov ^d^	21.3, CH_2_	1.92, ov^d^	21.1, CH_2_
15β	1.82, m		1.82, ov ^d^		1.66, m	
16	2.83, m	48.6, CH	2.82, m	48.7, CH	2.72, m	49.3, CH
17α	2.45, dd (13.2, 11.0)	27.7, CH_2_	2.43, dd (13.2, 10.2)	27.7, CH_2_	2.33, dd (13.0, 10.5)	26.8, CH_2_
17β	2.74, dd (13.2, 6.5)		2.71, dd (13.2, 6.0)		2.61, dd (13.0, 7.0)	
18		117.4, C		117.5, C		115.3, C
19		125.3, C		123.9, C		123.3, C
20	7.43, dd (6.7, 2.0)	118.7, CH	7.31, d (8.4)	119.3, CH	7.28, d (8.5)	118.9, CH
21	7.08, td (6.7, 2.0)	119.9, CH	7.04, dd (8.4, 1.8)	120.4, CH	6.92, dd (8.5, 2.0)	118.8, CH
22	7.10, td (6.7, 2.0)	120.7, CH		126.4, C		123.9, C
23	7.31, dd (6.7, 2.0)	111.7, CH	7.28, d (1.8)	111.6, CH	7.26, d (2.0)	111.3, CH
24		139.9, C		140.2, C		140.3, C
25	1.38, s	16.4, CH_3_	1.37, s	16.4, CH_3_	1.29, s	16.4, CH_3_
26	1.16, s	24.5, CH_3_	1.14, s	24.5, CH_3_	0.99, s	19.7, CH_3_
27		66.8, C		76.9, C		74.3, C
28α	3.64, dd (12.0, 2.0)	68.2, CH_2_	4.21, dd (13.2, 2.4)	65.0, CH_2_	3.88, s	67.9, CH_2_
28β	3.75, d (12.0)		3.69, d (13.2)			
29	1.07, s	18.9, CH_3_	1.37, s	17.3, CH_3_	1.30, s	21.9, CH_3_
1′				176.9, C		175.5, C
2′				72.5, C		71.3, C
3′			1.52, s	27.4, CH_3_	1.29, s	27.3, CH_3_
4′			1.53, s	27.3, CH_3_	1.29, s	27.3, CH_3_
13-OH					4.98, s	
27-OH					5.06, d (1.5)	
2′-OH					5.26, s	

^a^ 500 MHz. ^b^ 125 MHz. ^c^ 600 MHz. ^d^ overlapped signals.

**Table 3:** Compounds influence SCOs and 4-AP-induced SCOs.

Compounds	SCOs EC_50_ (μM, Mean ± SEM)		4-AP-induced SCOs EC_50_ (μM, Mean ± SEM)	
	Amplitude	Frequency	Amplitude	Frequency
6	10.28 ± 1.22	6.96 ± 0.73	28.45 ± 1.65	27.08 ± 2.94
17	3.86 ± 0.06	2.32 ± 0.67	3.70 ± 2.11	1.90 ± 1.04
18	1.85 ± 0.21	2.68 ± 0.04	3.88 ± 0.09	3.67 ± 0.01
24	5.62 ± 1.39	4.77 ± 0.14	6.05 ± 0.83	3.49 ± 0.51
9	7.48 ± 0.09	5.32 ± 3.92 ^I^	N/T	N/T
14	2.40 ± 0.57	0.26 ± 0.08 ^I^	N/T	N/T

Data represent mean values of five independent experiments; “N/T” means not tested. ”^I^” indicates increase in the SCO frequency.

## Data Availability

Data are contained within the article or Supplementary Materials.

## Supplementary Materials

The following are available online at https://www.mdpi.com/article/10.3390/md19050281/s1, tables for the experimental NMR data of **20** and computed NMR data of **24**, figures for the 2D correlations of **1**–**3**, MS, IR, UV, NMR spectra of **1**–**3**, **17**–**18** and **22**–**24**, the low energy conformers of the computed structures of **24**, and the in vitro effects of **6**, **9**, **14**, **17**, **18** and **24** on SCOs.
