# Supplementary material for: Neuronal Modulators from the Coral-Associated Fungi Aspergillus candidus
Source: Mar Drugs. 2021 May 19;19(5):281. doi: 10.3390/md19050281 (PMC8161303; doi:10.3390/md19050281)
Supplement: Supplementary file 1 [file marinedrugs-19-00281-s001.zip › marinedrugs-1211148-SI.pdf]

Neuronal Modulators from the Coral-Associated Fungi *Aspergillus candidus*

Gao-Yang Peng [1,2,3], Tibor Kurtán [4], Attila Mándi [4], Jing He [5], Zheng-Yu Cao [5], Hua Tang [6], Shui-Chun Mao [1,*] and Wen Zhang [2,3,*]

[1]School of Pharmacy, Nanchang University, 461 Bayi Road, Nanchang 330006, P. R. China

[2]Tongji University School of Medicine, 1239 Si-Ping Rd., Shanghai 200092, P. R. China

[3]School of Pharmacy, Second Military Medical University, 325 Guo-He Rd., Shanghai 200433, P. R. China

[4]Department of Organic Chemistry, University of Debrecen, POB 400, H-4002 Debrecen, Hungary

[5]State Key Laboratory of Natural Medicines and Department of TCM Pharmacology, School of Traditional Chinese Pharmacy, China Pharmaceutical University, 639 Long-Mian Ave., Nanjing 211198, P. R. China

[6]Institute of Translational Medicine, Shanghai University, 99 Shang-Da Rd., Shanghai 200444, P. R. China

*Correspondence: wenzhang1968@163.com (W.Z.); maoshuichun@ncu.edu.cn (S.-C.M.)

Table of contents

Table S1. ^{1}H and ^{13}C NMR Data for **20** S1

Table S2. Computed and experimental ^{13}C NMR data comparison of **24** S3

Figure S1. Selected COSY, HMBC, and NOESY correlations of **1-3** S4

Figure S2. MS of **1** S5

Figure S3. IR (film) of **1** S5

Figure S4. UV of **1** in MeCN S6

Figure S5. ^{1}H-NMR spectrum of **1** in $CDCl_3$ at 500 MHz S7

Figure S6. ^{13}C-NMR spectrum of **1** in $CDCl_3$ at 125 MHz S8

Figure S7. DEPT spectrum of **1** in $CDCl_3$ at 125 MHz S9

Figure S8. HSQC spectrum of **1** in $CDCl_3$ S10

Figure S9. COSY spectrum of **1** in $CDCl_3$ S10

Figure S10. HMBC spectrum of **1** in $CDCl_3$ S11

Figure S11. NOESY spectrum of **1** in $CDCl_3$ S11

Figure S12. MS of **2** S12

Figure S13. IR (film) of **2** S12

Figure S14. UV of **2** in MeCN S13

Figure S15. ^{1}H-NMR spectrum of **2** in CD_3OD at 500 MHz S14

Figure S16. ^{13}C-NMR spectrum of **2** in CD_3OD at 125 MHz S15

Figure S17. DEPT spectrum of **2** in CD_3OD at 125 MHz S16

Figure S18. HSQC spectrum of **2** in CD_3OD S17

Figure S19. COSY spectrum of **2** in CD_3OD S17

Figure S20. HMBC spectrum of **2** in CD_3OD S18

Figure S21. NOESY spectrum of **2** in CD_3OD S18

Figure S22. MS of **3** S19

Figure S23. IR (film) of **3** S19

Figure S24. UV of **3** in MeCN S20

Figure S25. ^{1}H-NMR spectrum of **3** in $CDCl_3$ at 500 MHz S21

Figure S26. ^{13}C-NMR spectrum of **3** in $CDCl_3$ at 125 MHz S22

Figure S27. DEPT spectrum of **3** in $CDCl_3$ at 125 MHz S23
Figure S28. HSQC spectrum of **3** in $CDCl_3$ S24
Figure S29. COSY spectrum of **3** in $CDCl_3$ S24
Figure S30. HMBC spectrum of **3** in $CDCl_3$ S25
Figure S31. NOESY spectrum of **3** in $CDCl_3$ S25
Figure S32. MS of **17** S26
Figure S33. IR (film) of **17** S26
Figure S34. UV of **17** in MeCN S27
Figure S35. ^{1}H-NMR spectrum of **17** in DMSO at 600 MHz S28
Figure S36. ^{13}C-NMR spectrum of **17** in DMSO at 125 MHz S29
Figure S37. DEPT spectrum of **17** in DMSO at 125 MHz S30
Figure S38. HSQC spectrum of **17** in DMSO S31
Figure S39. COSY spectrum of **17** in DMSO S31
Figure S40. HMBC spectrum of **17** in DMSO S32
Figure S41. NOESY spectrum of **17** in DMSO S32
Figure S42. MS of **18** S33
Figure S43. IR (film) of **18** S33
Figure S44. UV of **18** in MeCN S34
Figure S45. ^{1}H-NMR spectrum of **18** in DMSO at 500 MHz S35
Figure S46. ^{13}C-NMR spectrum of **18** in DMSO at 125 MHz S36
Figure S47. DEPT spectrum of **18** in DMSO at 125 MHz S37
Figure S48. HSQC spectrum of 18 in DMSO S38
Figure S49. COSY spectrum of **18** in DMSO S38
Figure S50. HMBC spectrum of **18** in DMSO S39
Figure S51. NOESY spectrum of **18** in DMSO S39
Figure S52. MS of **22** S40
Figure S53. IR (film) of **22** S40
Figure S54. UV of **22** in MeCN S41
Figure S55. ^{1}H-NMR spectrum of **22** in $CDCl_3$ at 500 MHz S42

Figure S56. ^{13}C-NMR spectrum of **22** in $CDCl_3$ at 125 MHz S43
Figure S57. DEPT spectrum of **22** in $CDCl_3$ at 125 MHz S44
Figure S58. HSQC spectrum of **22** in $CDCl_3$ S45
Figure S59. COSY spectrum of **22** in $CDCl_3$ S45
Figure S60. HMBC spectrum of **22** in $CDCl_3$ S46
Figure S61. NOESY spectrum of **22** in $CDCl_3$ S46
Figure S62. MS of **23** S47
Figure S63. IR (film) of **23** S47
Figure S64. UV of **23** in MeCN S48
Figure S65. ^{1}H-NMR spectrum of **23** in $CDCl_3$ at 600 MHz S49
Figure S66. ^{13}C-NMR spectrum of **23** in $CDCl_3$ at 125 MHz S50
Figure S67. DEPT spectrum of **23** in $CDCl_3$ at 125 MHz S51
Figure S68. HSQC spectrum of **23** in $CDCl_3$ S52
Figure S69. COSY spectrum of **23** in $CDCl_3$ S52
Figure S70. HMBC spectrum of **23** in $CDCl_3$ S53
Figure S71. NOESY spectrum of **23** in $CDCl_3$ S53
Figure S72. MS of **24** S54
Figure S73. IR (film) of **24** S54
Figure S74. UV of **24** in MeCN S55
Figure S75. ^{1}H-NMR spectrum of **24** in DMSO at 500 MHz S56
Figure S76. ^{13}C-NMR spectrum of **24** in DMSO at 125 MHz S57
Figure S77. DEPT spectrum of **24** in DMSO at 125 MHz S58
Figure S78. HSQC spectrum of **24** in DMSO S59
Figure S79. COSY spectrum of **24** in DMSO S59
Figure S80. HMBC spectrum of **24** in DMSO S60
Figure S81. NOESY spectrum of **24** in DMSO S60
Figure S82. NOESY spectrum (enlarged) of **24** in DMSO S61
Figure S83. Structure and population of the low-energy conformers (> 1%) of (3*S*,4*R*,9*R*,13*S*,16*S*,27*S*)-**24** S61
Figure S84. Structure and population of the low-energy conformers S62

(> 1%) of (3*S*,4*R*,9*S*,13*S*,16*S*,27*S*)-**24**.

Figure S85. Effects of **6** on SCOs *in vitro* S62

Figure S86. Effects of **9** on SCOs *in vitro* S63

Figure S87. Effects of **14** on SCOs *in vitro* S63

Figure S88. Effects of **17** on SCOs *in vitro* S64

Figure S89. Effects of **18** on SCOs *in vitro* S64

Figure S90. Effects of **24** on SCOs *in vitro* S65

Table S1. ^{1}H and ^{13}C NMR Data for **20**

	**20** (in DMSO)		**20** (in $CDCl_3$)	
position	δ_H[a] (J in Hz)	δ_C,[b] type	δ_H[a] (J in Hz)	δ_C,[b] type
1-NH	10.79 s		7.69 s	
2		154.2, C		152.9, C
3		51.4, C		52.1, C
4		38.6, C		39.4, C
5α	1.94, ov[c]	26.2, CH_2	1.86, ov[c]	27.2, CH_2
5β	2.40 dd (12.0, 9.5)		2.60, ov[c]	
6α	1.95, ov[c]	30.1, CH_2	2.16 m	30.6, CH_2
6β	2.55 m		2.61, ov[c]	
7		93.6, C		94.0, C
9	4.64 d (2.5)	78.6, CH	4.76 d (2.5)	79.6, CH
10		195.8, C		196.2, C
11	6.13 s	120.0, CH	6.21 s	120.4, CH
12		159.2, C		159.9, C
13		77.1, C		78.7, C
14α	2.12 dt (13.2, 3.0)	31.6, CH_2	1.99, ov[c]	33.8, CH_2
14β	1.76 td (13.2, 4.5)		1.95, ov[c]	
15α	1.95, ov[c]	20.9, CH_2	2.08, ov[c]	21.3, CH_2
15β	1.66 m		1.82, ov[c]	
16	2.72 m	48.5, CH	2.82 m	48.7, CH
17α	2.31 dd (13.0, 10.5)	27.1, CH_2	2.42 dd (13.0, 10.5)	27.7, CH_2
17β	2.61 dd (13.0, 6.0)		2.71 dd (13.0, 6.5)	
18		115.1, C		117.4, C
19		123.4, C		123.9, C
20	7.27 d (8.0)	118.8, CH	7.32 d (8.0)	119.3, CH
21	6.92 dd (8.0, 1.7)	118.7, CH	7.04 dd (8.0, 1.5)	120.4, CH
22		123.8, C		126.4, C
23	7.26 d (1.7)	111.4, CH	7.27 d (1.5)	111.6, CH
24		140.3, C		140.2, C
25	1.31 s	16.3, CH_3	1.37 s	16.4, CH_3
26	1.01 s	23.5, CH_3	1.15 s	24.5, CH_3
27		75.8, C		76.4, C
28α	4.12 dd (13.2, 2.5)	64.2, CH_2	4.24 dd (13.0, 2.5)	65.1, CH_2
28β	3.69 d (13.2)		3.66 d (13.0)	
29	1.18 s	16.5, CH_3	1.34 s	16.9, CH_3
1′		171.3, C		171.8, C
2′		78.0, C		78.8, C
3′	1.52 s	24.0, CH_3	1.62 s	24.5, CH_3

4′	1.54 s	24.3, CH_3	1.62 s	24.9, CH_3
5′		169.5, C		170.1, C
6′	2.05 s	20.9, CH_3	2.09 s	21.3, CH_3
13-OH	5.17 s			

[b]500 MHz; [b]125MHz; [c]overlapped signals.

Table S2. Comparison of the experimental and the mPW1PW91/6-311+G(2d,p)// B3LYP/6-31+G(d,p) ^{13}C NMR data of (3*S*,4*R*,9*R*,13*S*,16*S*,27*S*)-**24** and (3*S*,4*R*,9*S*,13*S*,16*S*,27*S*)-**24**.

Carbon	Exp.	calcd (9*R*)	calcd (9*S*)	Δδ (9*R*)	Δδ (9*S*)
C-2	153.9	150.21	150.42	3.69	3.48
C-3	50.4	54.80	54.90	4.40	4.50
C-4	42.7	47.91	47.71	5.21	5.01
C-5	30.6	32.71	33.00	2.11	2.40
C-6	111.5	117.68	118.90	6.18	7.40
C-7	145.0	147.95	147.95	2.95	2.95
C-9	82.1	81.62	82.57	0.48	0.47
C-10	194.7	200.87	198.58	6.17	3.88
C-11	116.1	116.33	116.65	0.23	0.55
C-12	154.5	156.48	157.67	1.98	3.17
C-13	73.8	78.40	77.22	4.60	3.42
C-14	31.7	33.46	33.17	1.76	1.47
C-15	21.1	22.40	22.48	1.30	1.38
C-16	49.3	50.14	50.18	0.84	0.88
C-17	26.8	28.25	28.29	1.45	1.49
C-18	115.3	118.96	118.87	3.66	3.57
C-19	123.3	123.87	123.87	0.57	0.57
C-20	118.9	118.99	118.96	0.09	0.06
C-21	118.8	121.41	121.34	2.61	2.54
C-22	123.9	135.76	135.68	11.86	11.78
C-23	111.3	110.79	110.78	0.51	0.52
C-24	140.3	139.80	139.77	0.50	0.53
C-25	16.4	15.28	15.29	1.12	1.11
C-26	19.7	18.06	18.28	1.64	1.42
C-27	74.3	75.86	75.13	1.56	0.83
C-28	67.9	69.30	69.89	1.40	1.99
C-29	21.9	21.65	18.97	0.25	2.93
C-1'	175.5	179.61	180.01	4.11	4.51
C-2'	71.3	73.74	74.04	2.44	2.74
C-3'	27.3	26.32	24.89	0.98	2.41
C-4'	27.3	24.81	25.89	2.49	1.41
CMAE	N/A	N/A	N/A	2.55	2.63

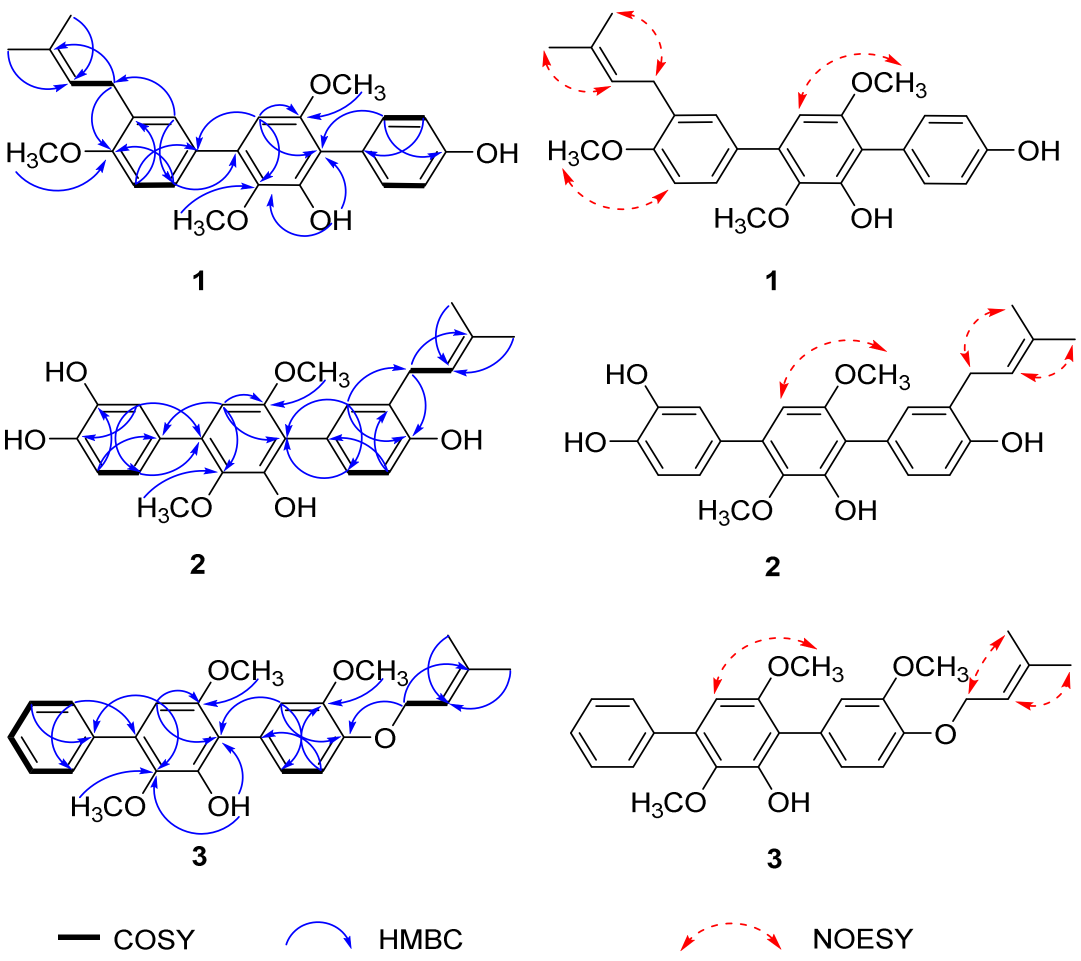

Figure S1. Selected COSY, HMBC, and NOESY correlations of **1-3**

Fragmentor Voltage	Collision Energy	Ionization Mode
120	0	ESI

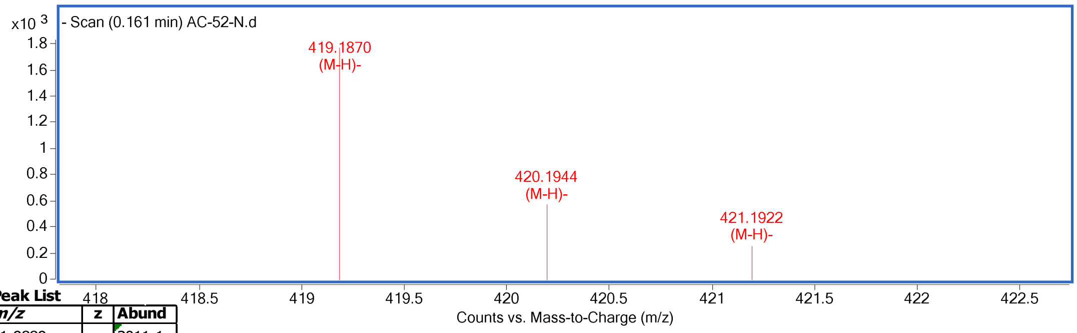

Peak List

m/z	z	Abund
61.9889		3011.1
160.8416		3595.8
162.8392		2249.8
174.9561		2148
186.1143	1	4905
365.2476	1	3342.4
459.2076	1	2958.2
966.0007	1	9462.3
967.002	1	2290.7
982.9938	1	2743.9

Formula Calculator Element Limits

Element	Min	Max
C	3	80
H	0	120
O	0	6
N	0	0
S	0	0
Cl	0	0
F	0	0

Formula Calculator Results

Formula	Best	Mass	Tgt Mass	Diff (ppm)	Ion Species	Score
C26 H28 O5	TRUE	420.1944	420.1937	-1.8	C26 H27 O5	75.61

Figure S2. MS of **1**

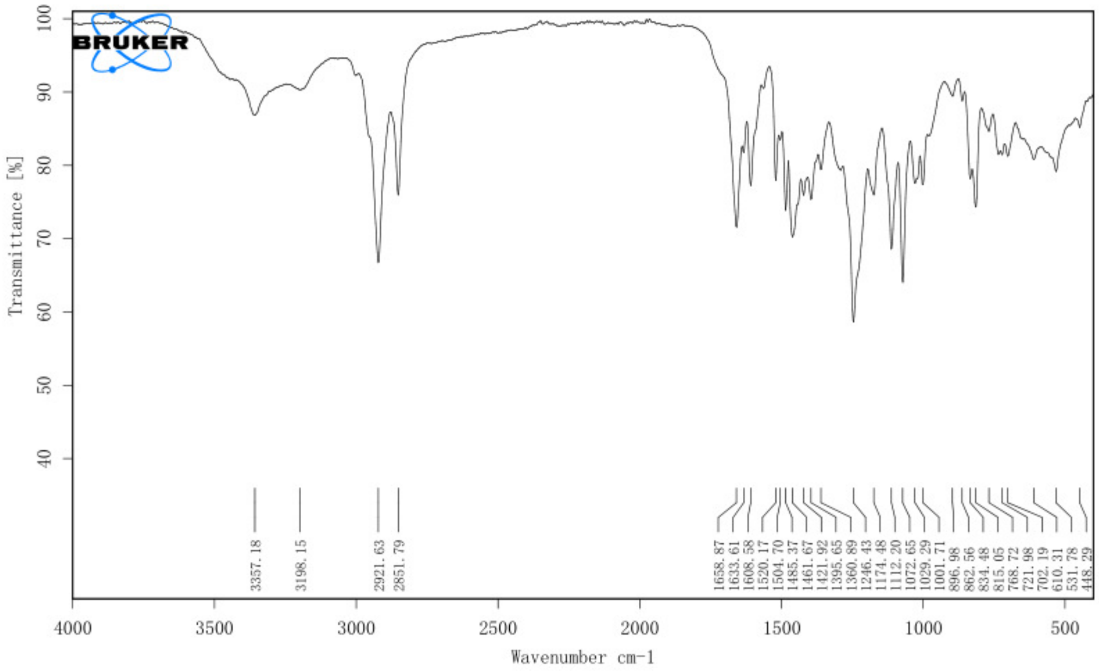

Figure S3. IR (film) of **1**

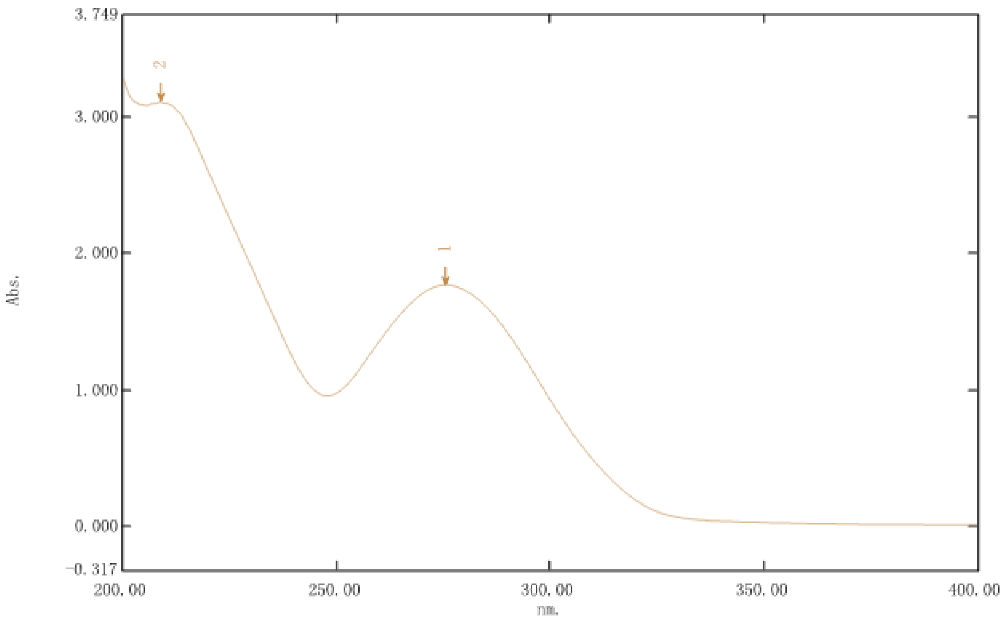

< 峰值检测表 >

No.	P/V	波长(nm)	Abs.	描述
1	T	275.80	1.770	
2	T	209.00	3.112	
3	↓	247.80	0.956	
4	↓	205.40	3.080	

Figure S4. UV of **1** in MeCN

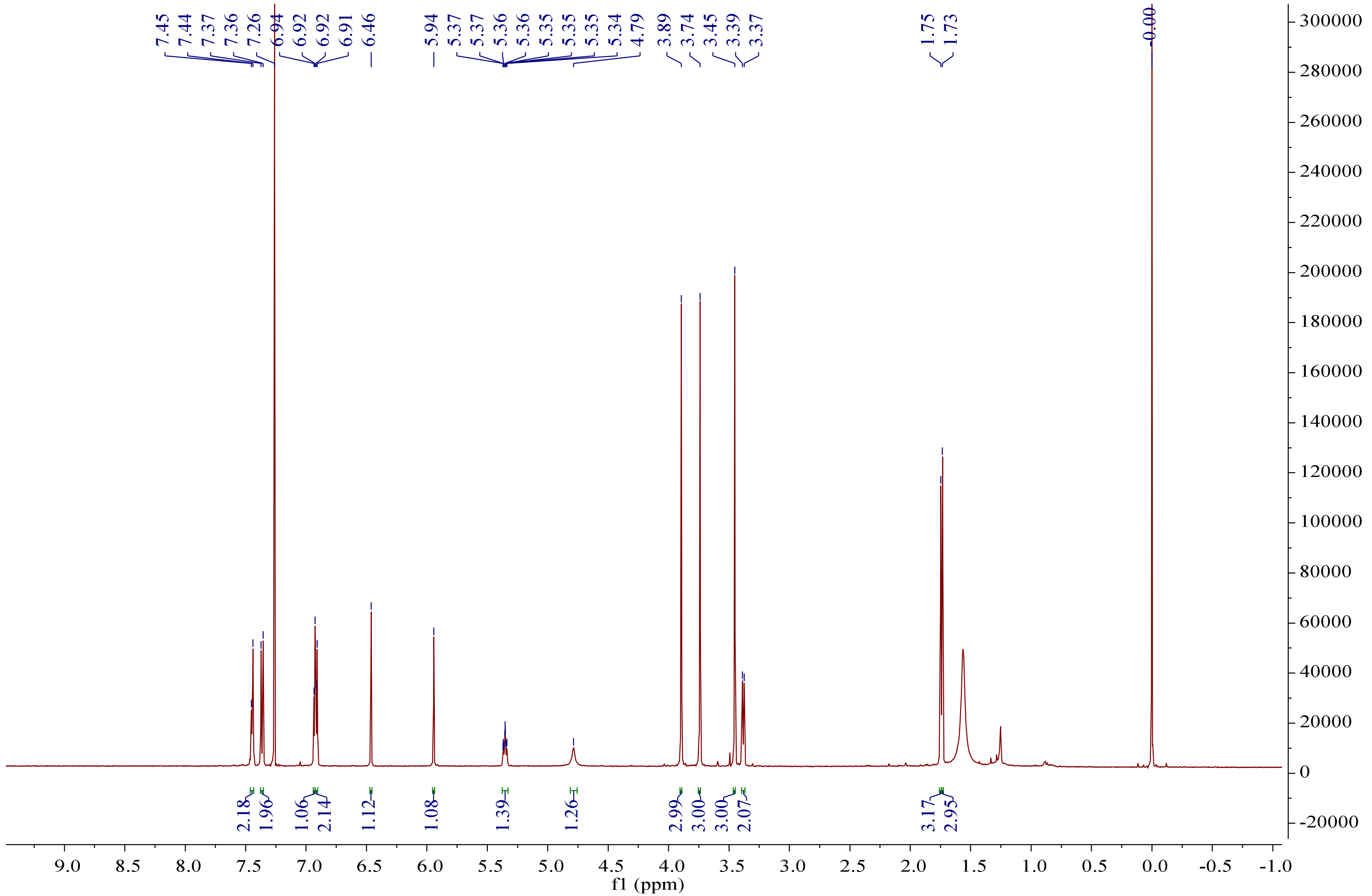

Figure S5. ^{1}H-NMR spectrum of **1** in $CDCl_3$ at 500 MHz

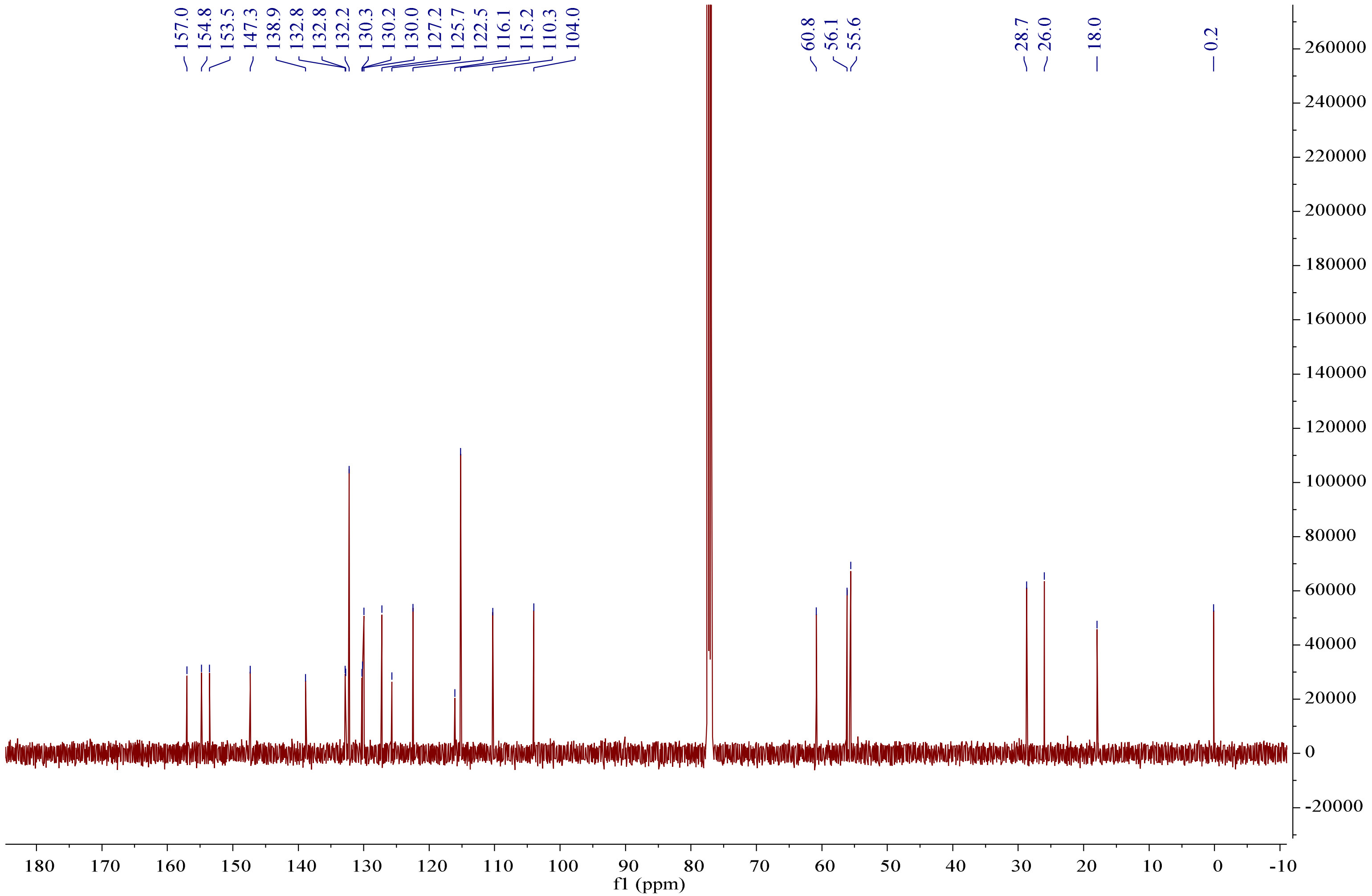

Figure S6. ^{13}C-NMR spectrum of **1** in $CDCl_3$ at 125 MHz

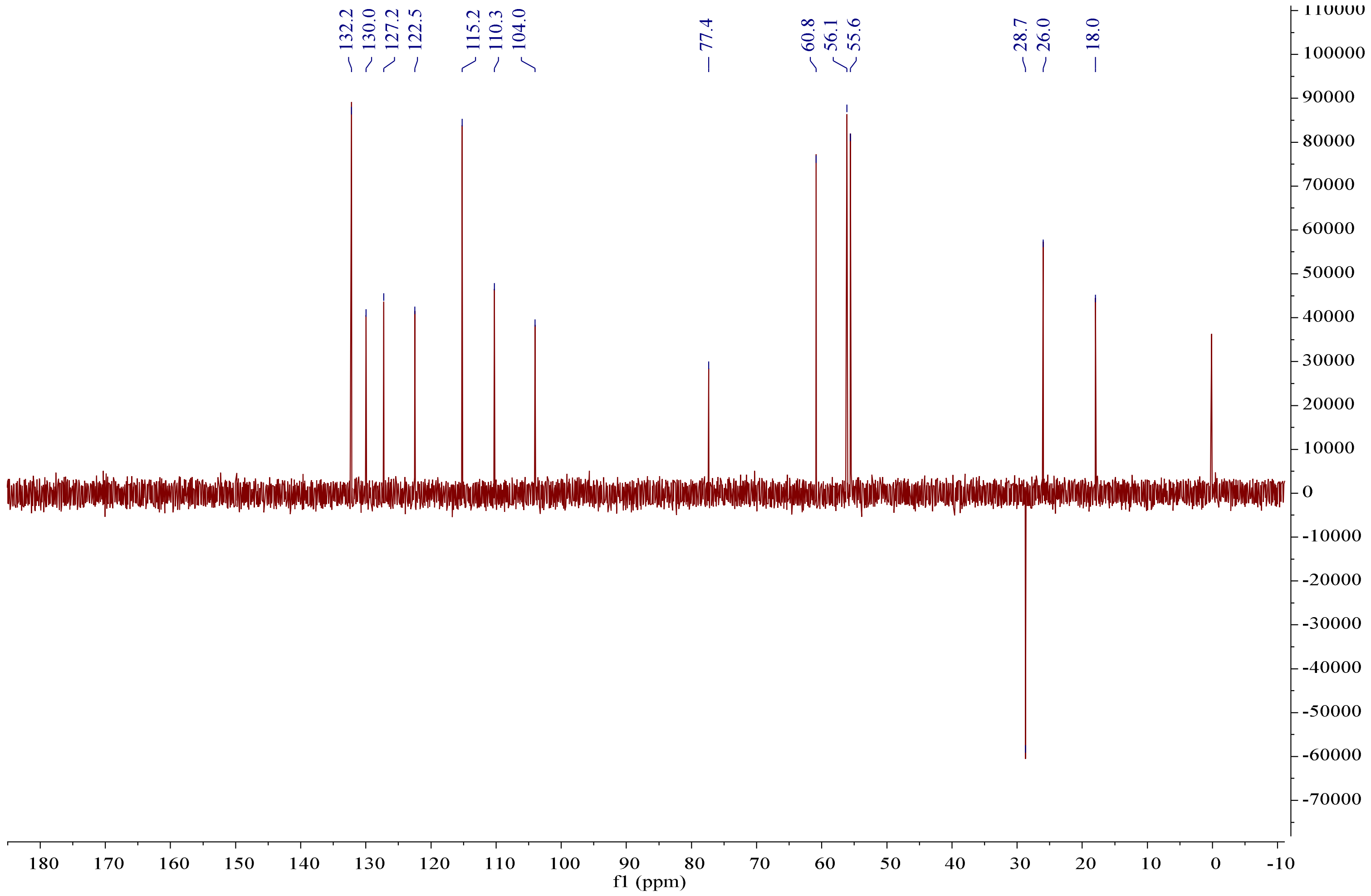

Figure S7. DEPT spectrum of **1** in $CDCl_3$ at 125 MHz

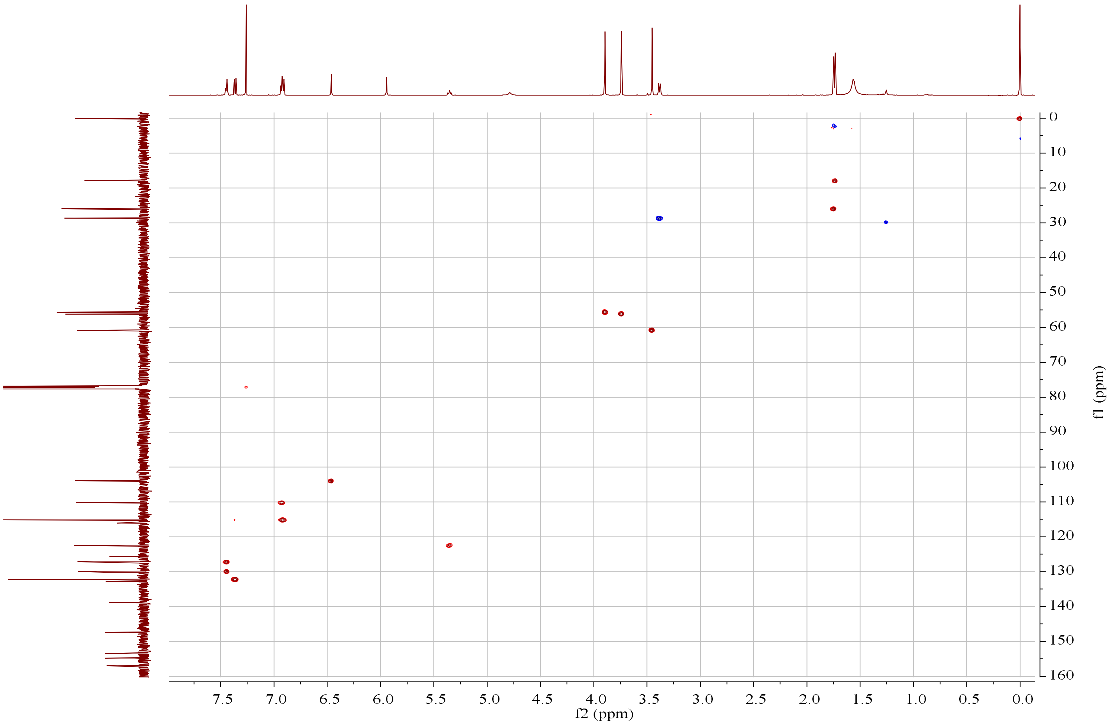

Figure S8. HSQC spectrum of **1** in $CDCl_3$

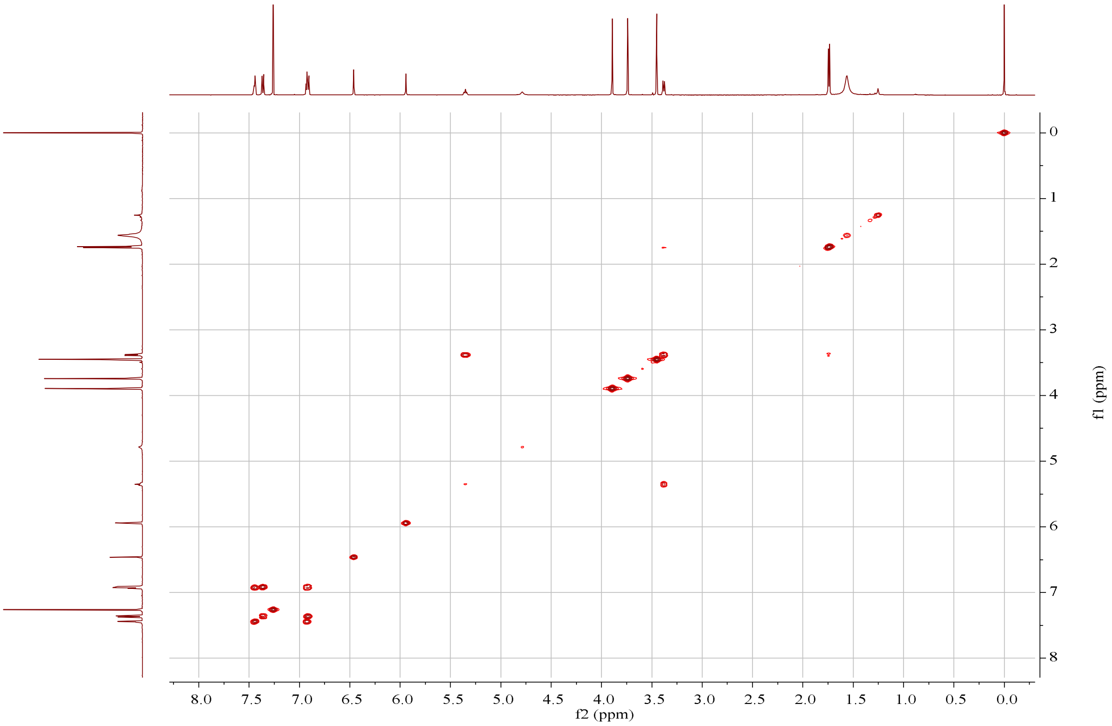

Figure S9. COSY spectrum of **1** in $CDCl_3$

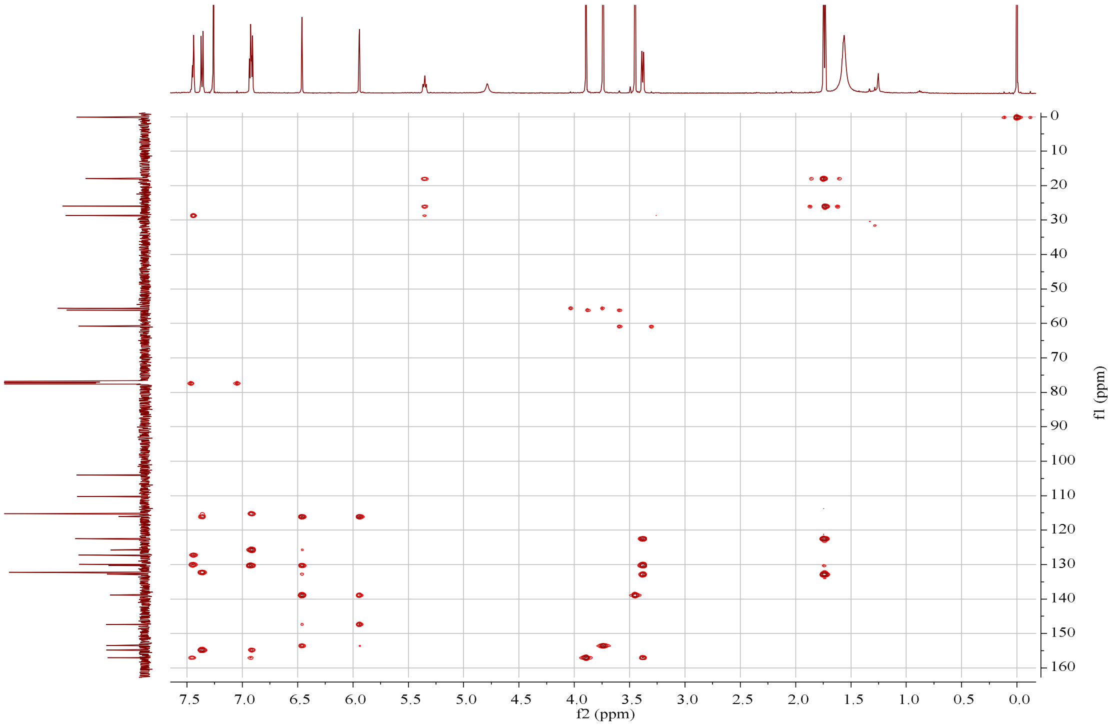

Figure S10. HMBC spectrum of **1** in $CDCl_3$

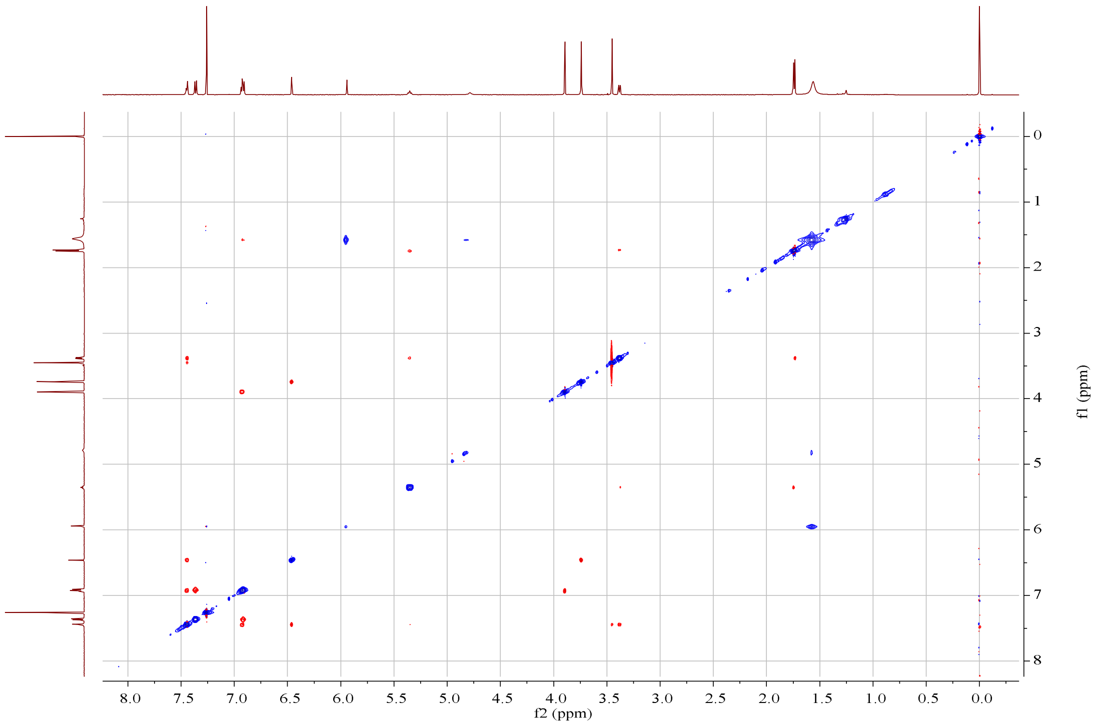

Figure S11. NOESY spectrum of **1** in $CDCl_3$

User Spectra

Fragmentor Voltage	Collision Energy	Ionization Mode
120	0	ESI

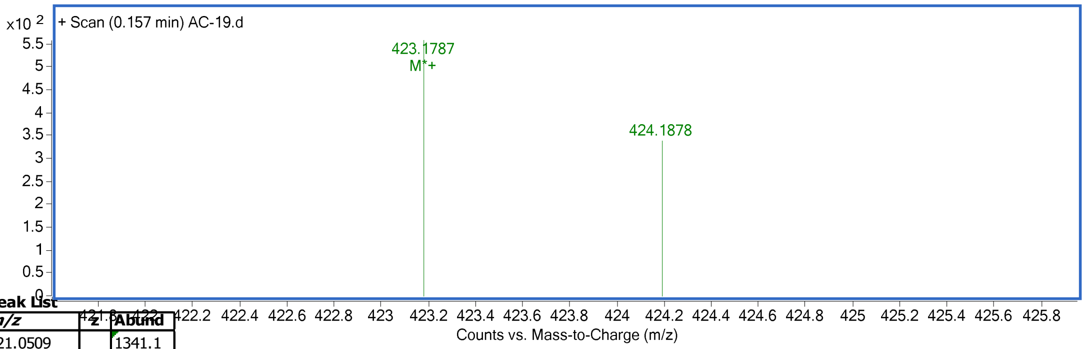

Peak List

m/z	z	Abund
121.0509		1341.1
130.1598		2771.7
141.9601	1	2468
158.0049		1783.1
415.2092	2	1746
437.1929	1	5690.4
438.1974	1	1720.7
453.1664	1	3528.3
454.1678	1	1111.3
922.0098	1	2463.2

Formula Calculator Element Limits

Element	Min	Max
C	3	80
H	0	120
O	0	6
N	0	0
S	0	0
Cl	0	0
F	0	0

Formula Calculator Results

Formula	Best	Mass	Tgt Mass	Diff (ppm)	Ion Species	Score
C25 H27 O6	TRUE	423.1793	423.1808	3.54	C25 H27 O6	42.35

Figure S12. MS of **2**

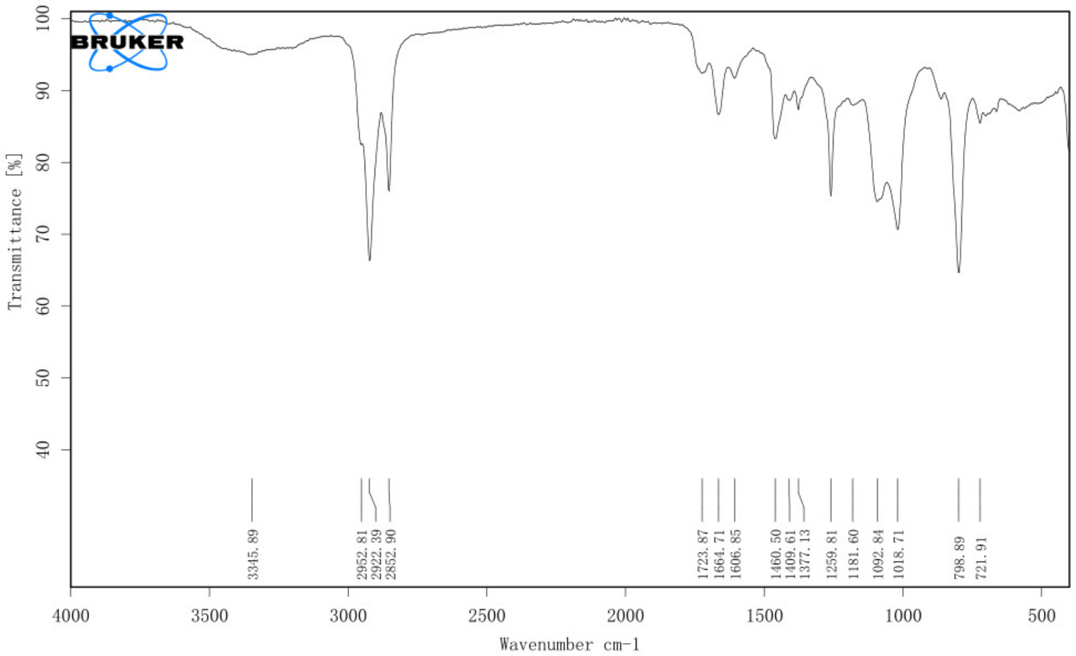

Figure S13. IR (film) of **2**

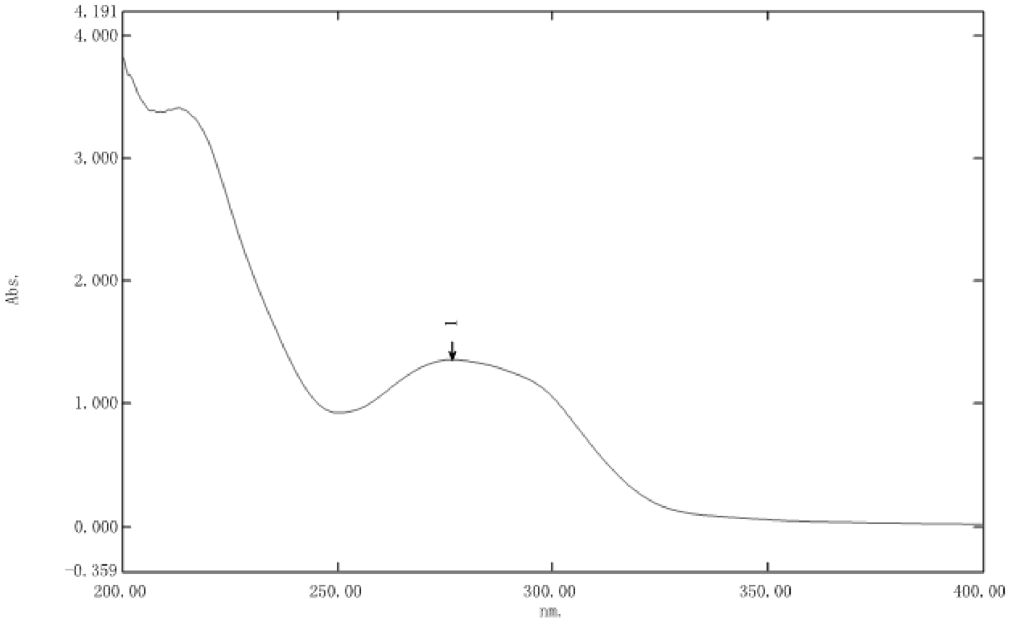

< 峰值检测表 >

No.	P/V	波长(nm)	Abs.	描述
1		276.60	1.359	
2		250.20	0.927	

Figure S14. UV of **2** in MeCN

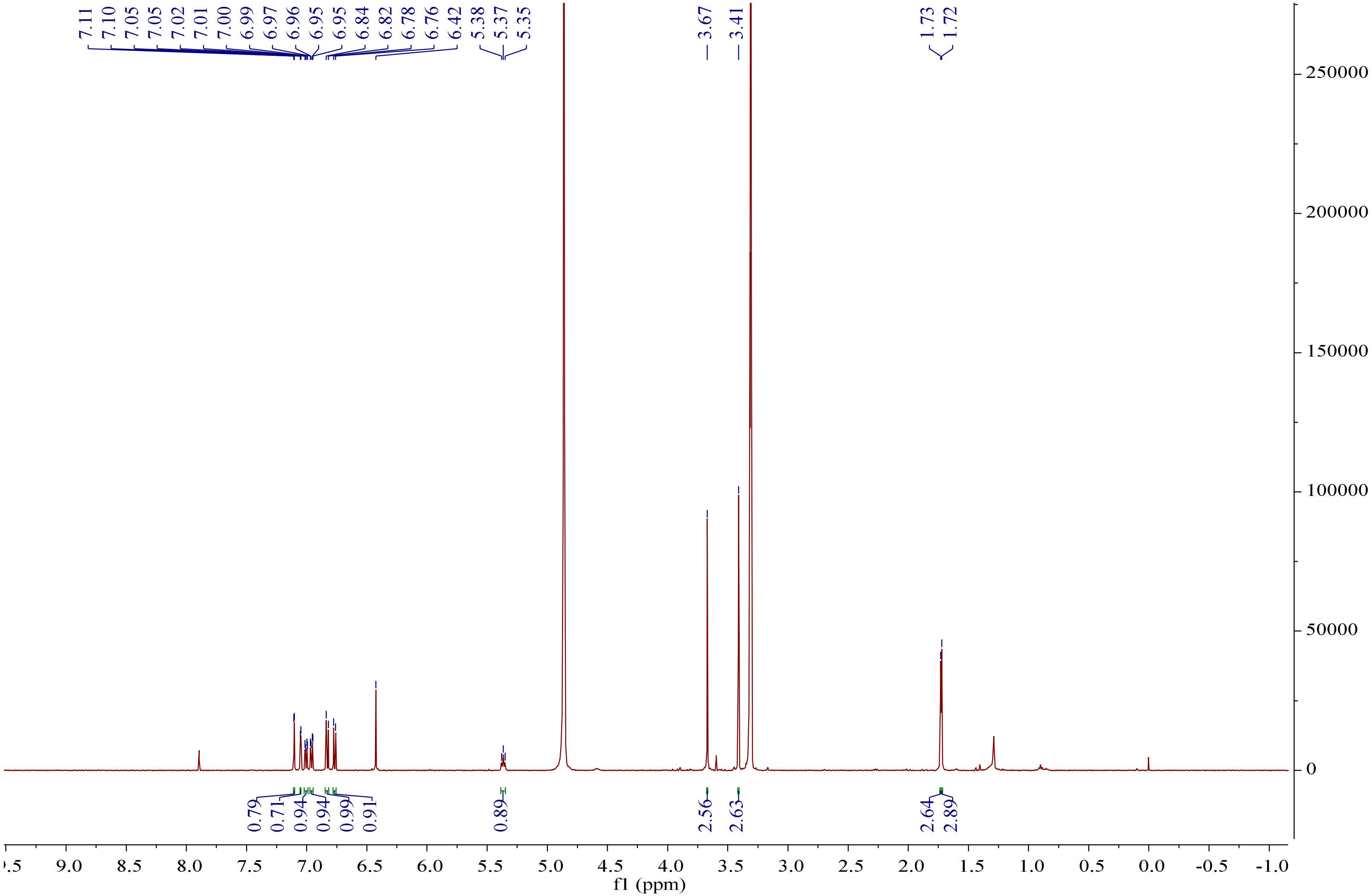

Figure S15. ^{1}H-NMR spectrum of **2** in CD_3OD at 500 MHz

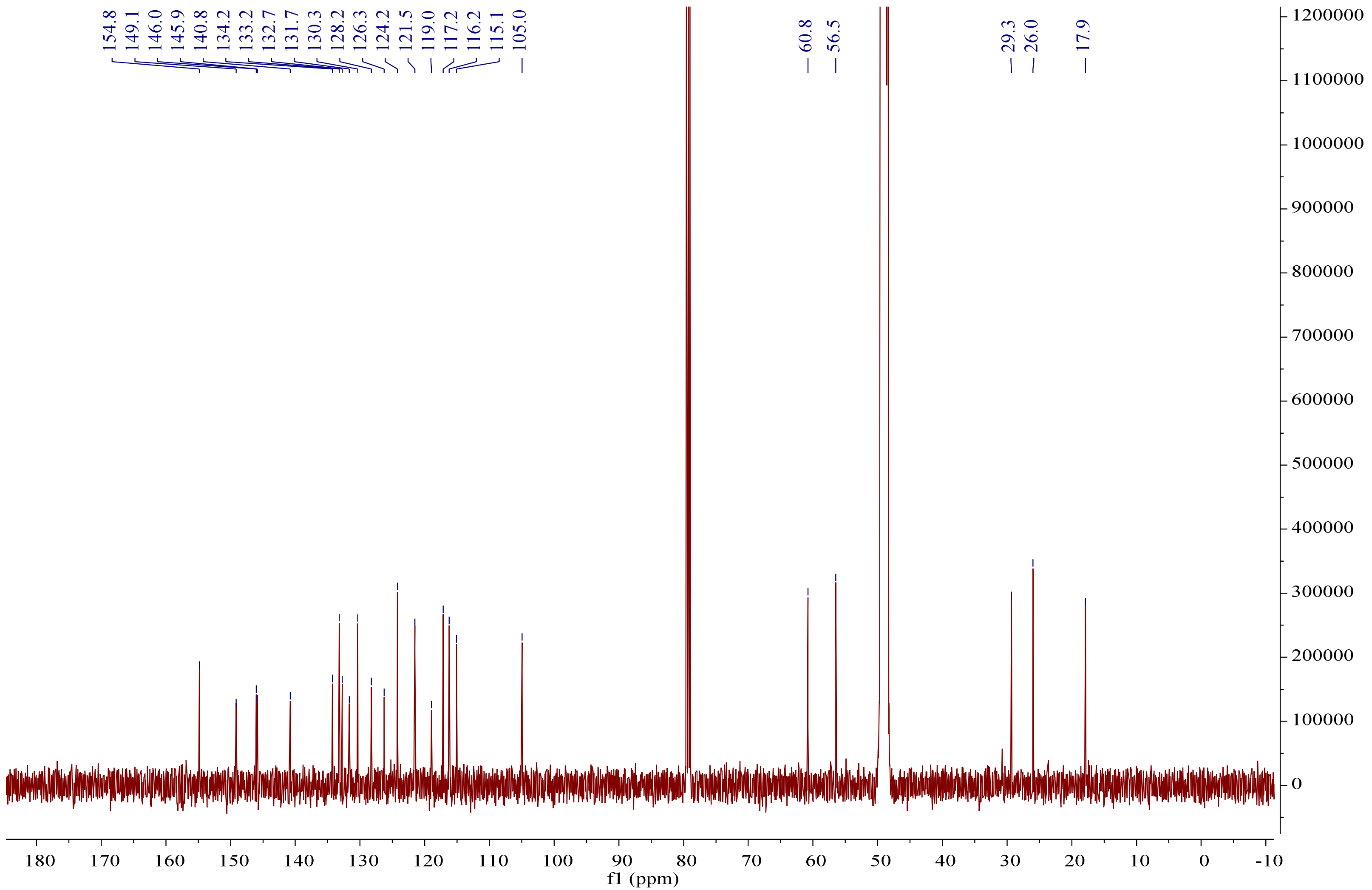

Figure S16. ^{13}C-NMR spectrum of **2** in CD_3OD at 125 MHz

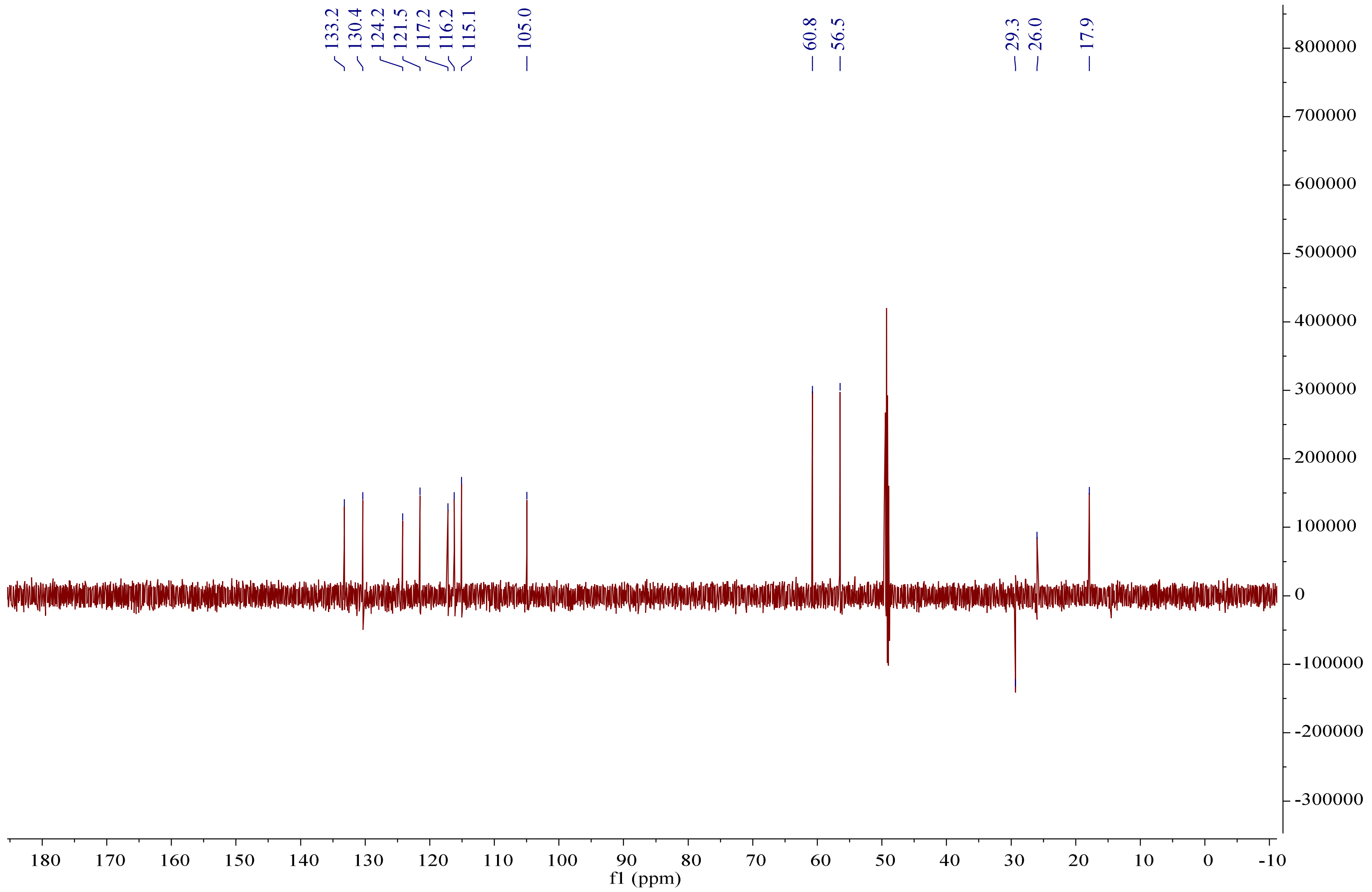

Figure S17. DEPT spectrum of **2** in CD_3OD at 125 MHz

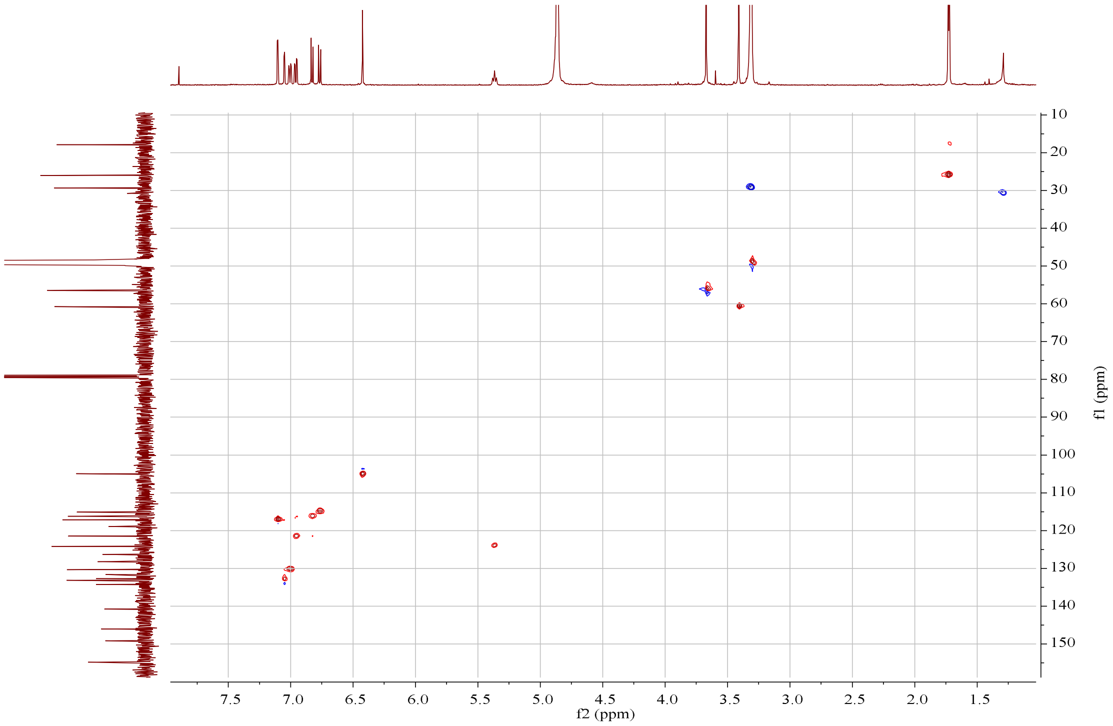

Figure S18. HSQC spectrum of **2** in CD_3OD

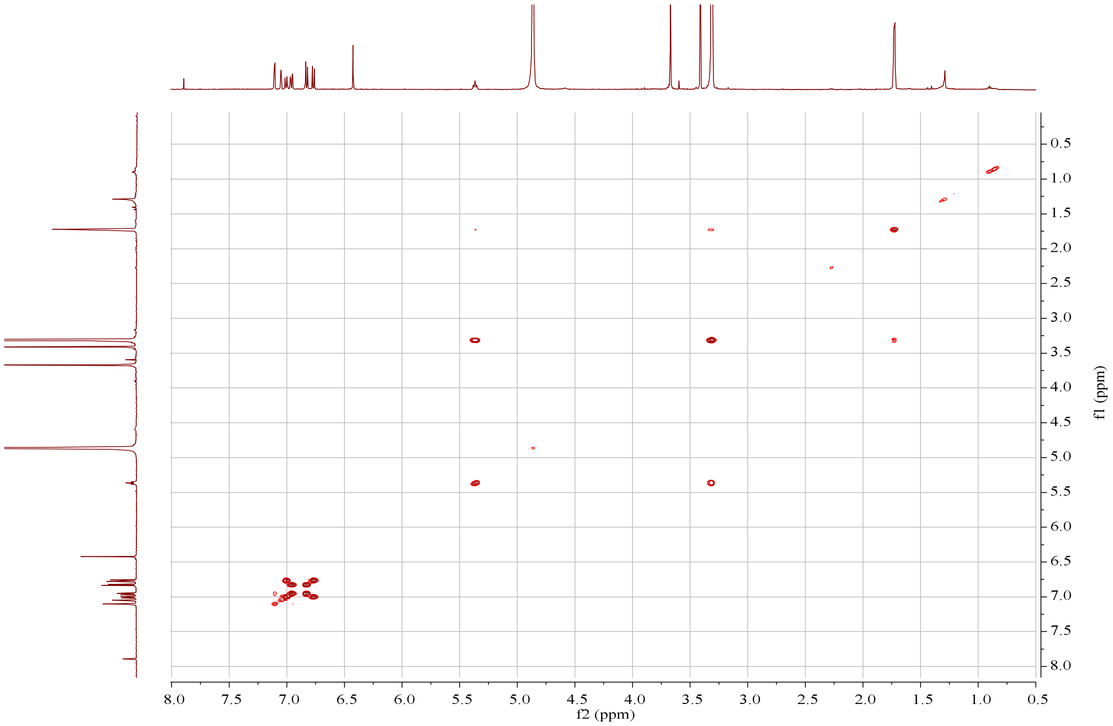

Figure S19. COSY spectrum of **2** in CD_3OD

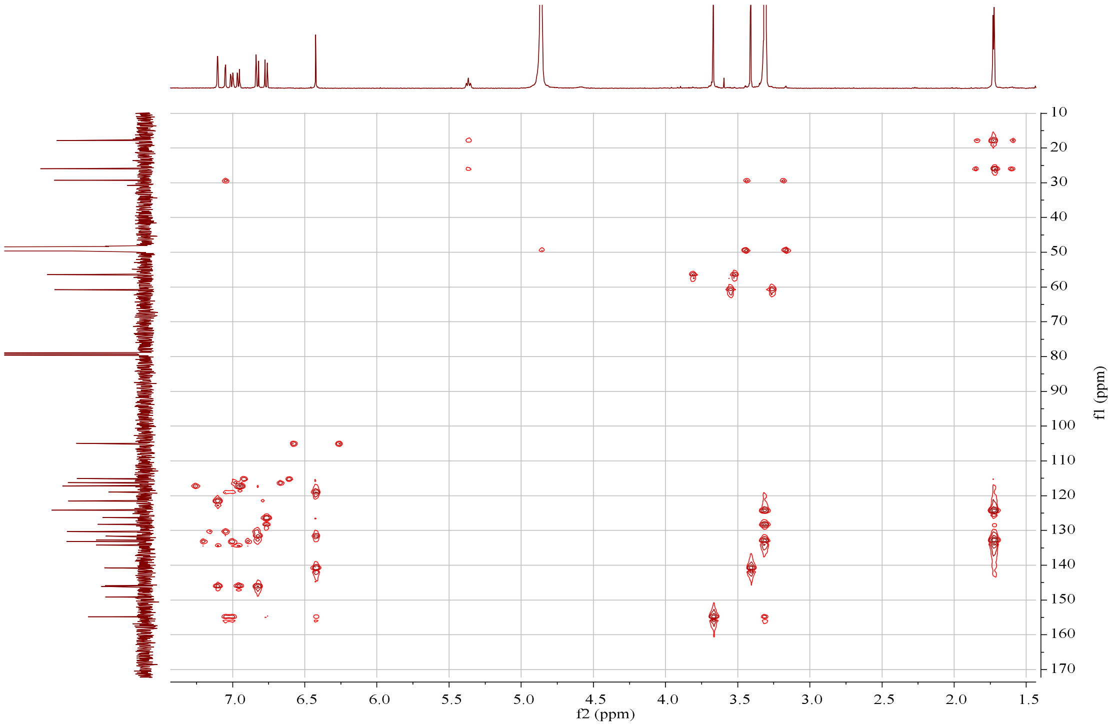

Figure S20. HMBC spectrum of **2** in CD_3OD

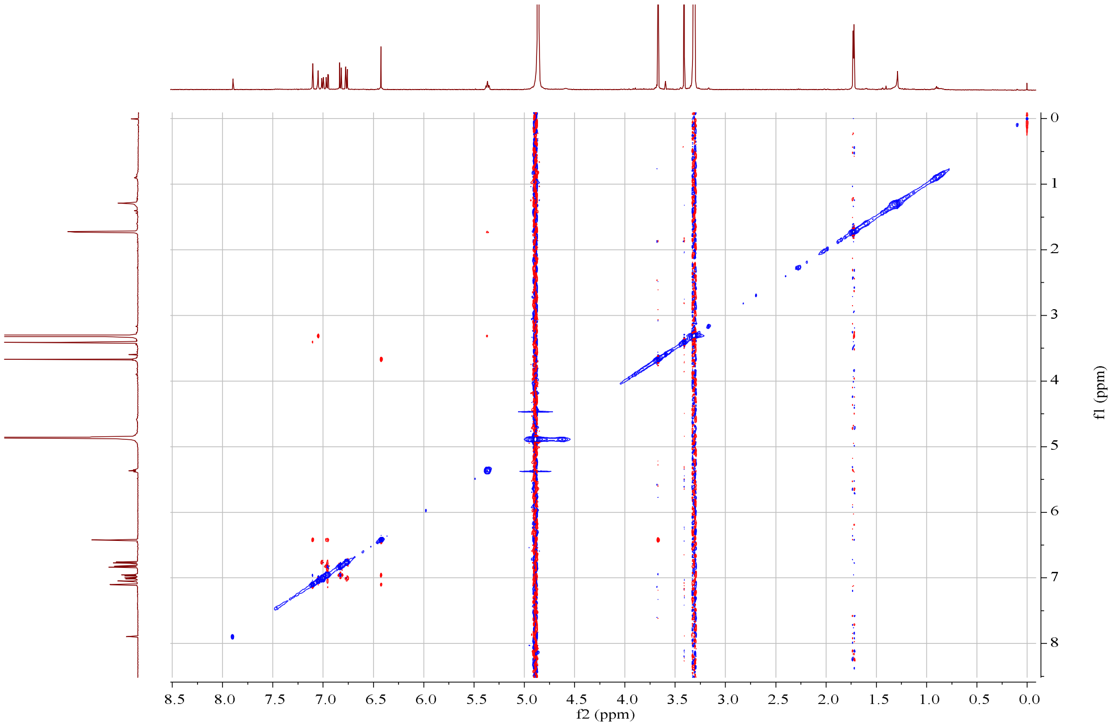

Figure S21. NOESY spectrum of **2** in CD_3OD

User Spectra

Fragmentor Voltage	Collision Energy	Ionization Mode
120	0	ESI

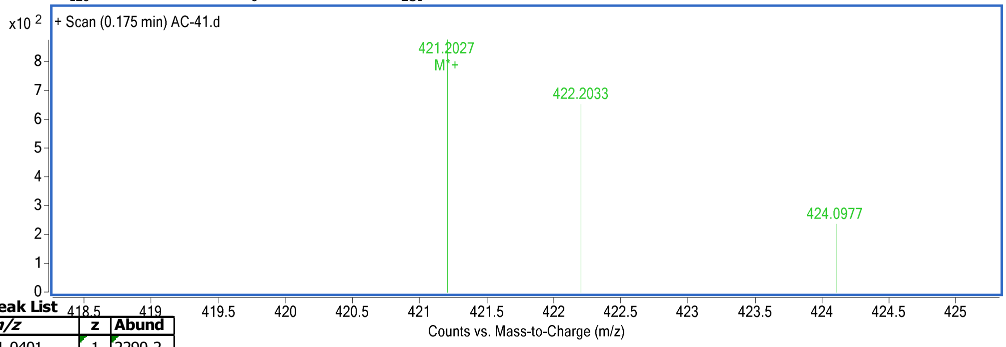

Peak List

m/z	z	Abund
61.0401	1	2290.2
130.1594		1682.6
141.9597	1	2106.2
353.1387	1	1444.3
353.2696	1	1516.4
415.2155	1	3666.3
437.195	1	8396.4
438.199	1	2190.5
453.1683	1	3686
922.0098	1	1940.9

Formula Calculator Element Limits

Element	Min	Max
C	3	80
H	0	120
O	0	5
N	0	0
S	0	0
Cl	0	0
F	0	0

Formula Calculator Results

Formula	Best	Mass	Tgt Mass	Diff (ppm)	Ion Species	Score
C26 H29 O5	TRUE	421.2033	421.2015	-4.25	C26 H29 O5	40.22

Figure S22. MS of **3**

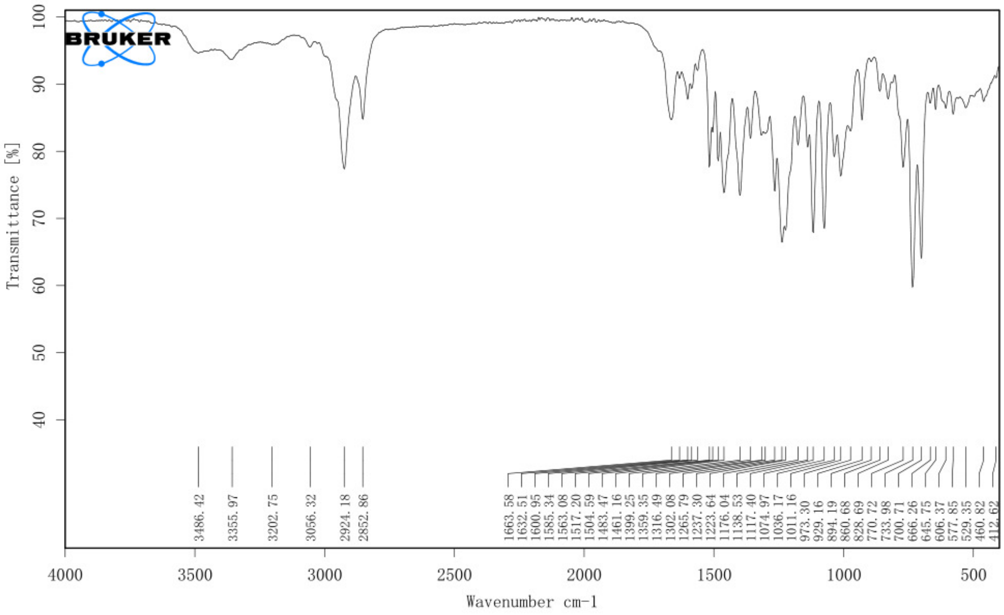

Figure S23. IR (film) of **3**

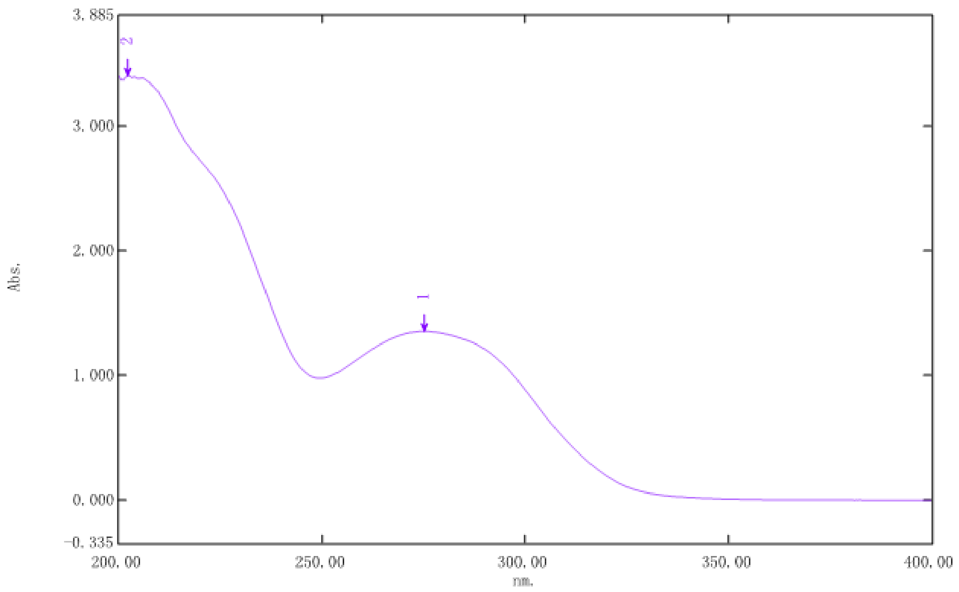

< 峰值检测表 >

No.	P/V	波长(nm)	Abs.	描述
1		275.20	1.355	
2		202.40	3.399	
3		249.60	0.980	

Figure S24. UV of **3** in MeCN

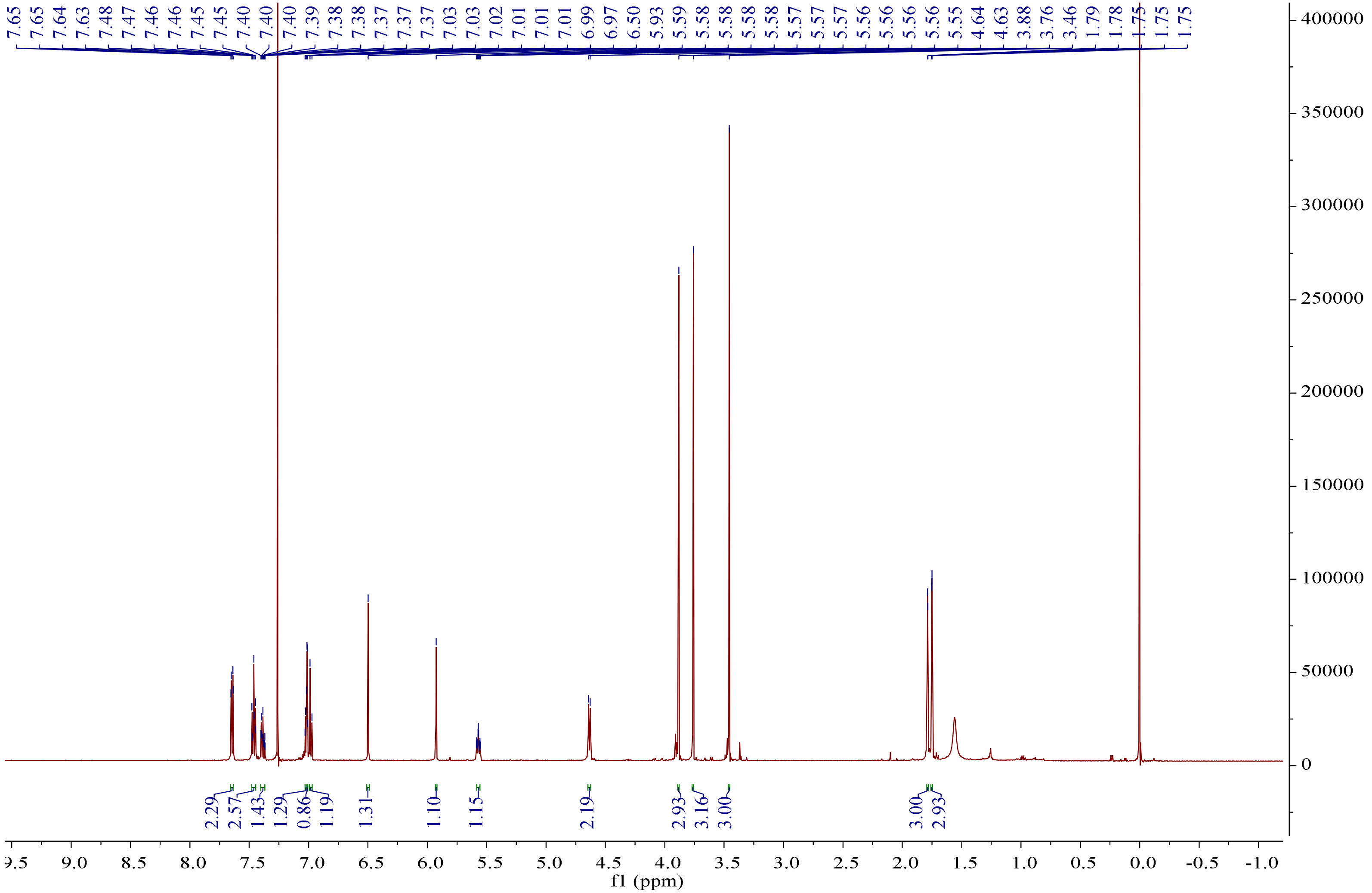

Figure S25. ^{1}H-NMR spectrum of **3** in $CDCl_3$ at 500 MHz

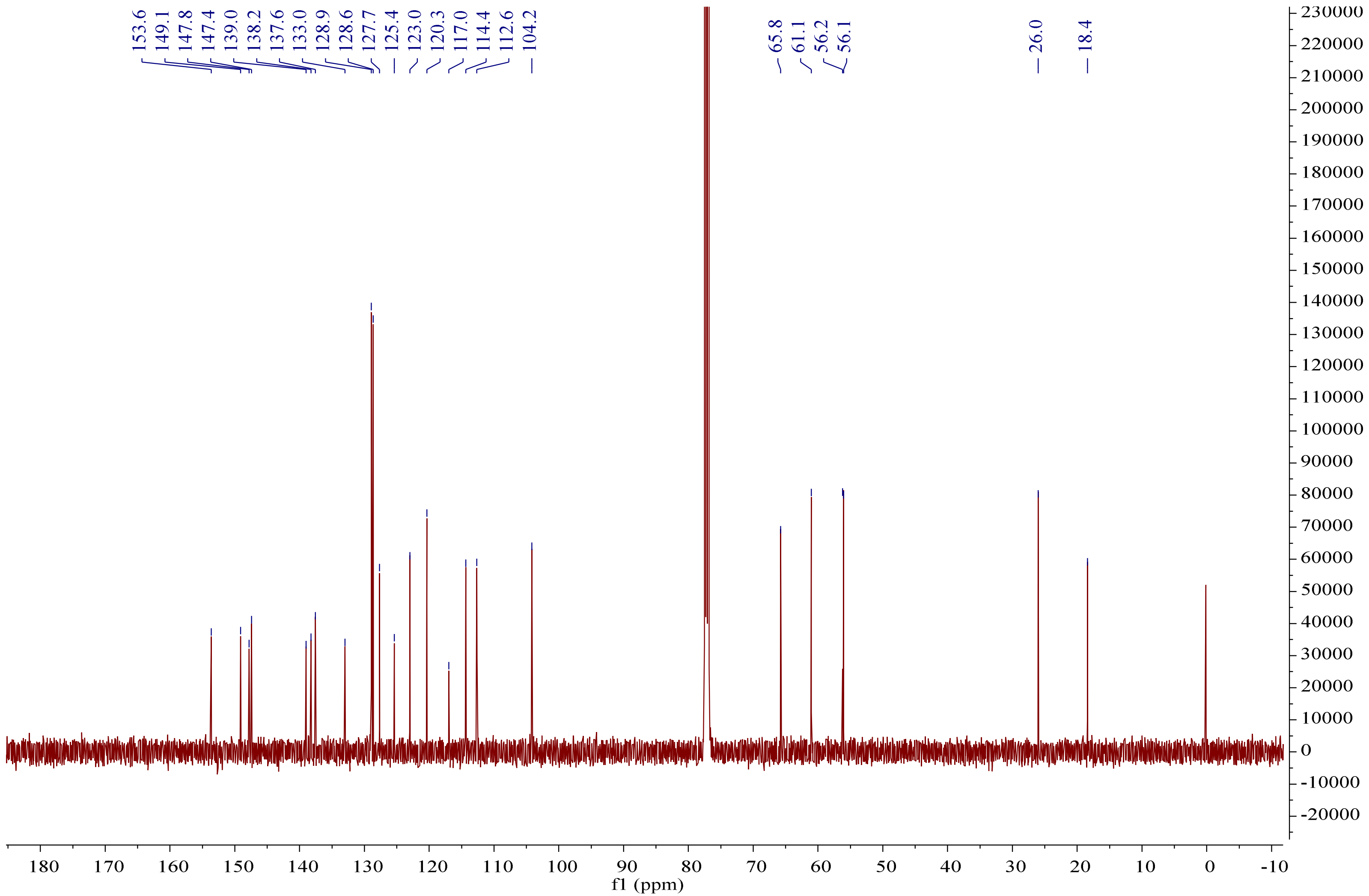

Figure S26. ^{13}C-NMR spectrum of **3** in $CDCl_3$ at 125 MHz

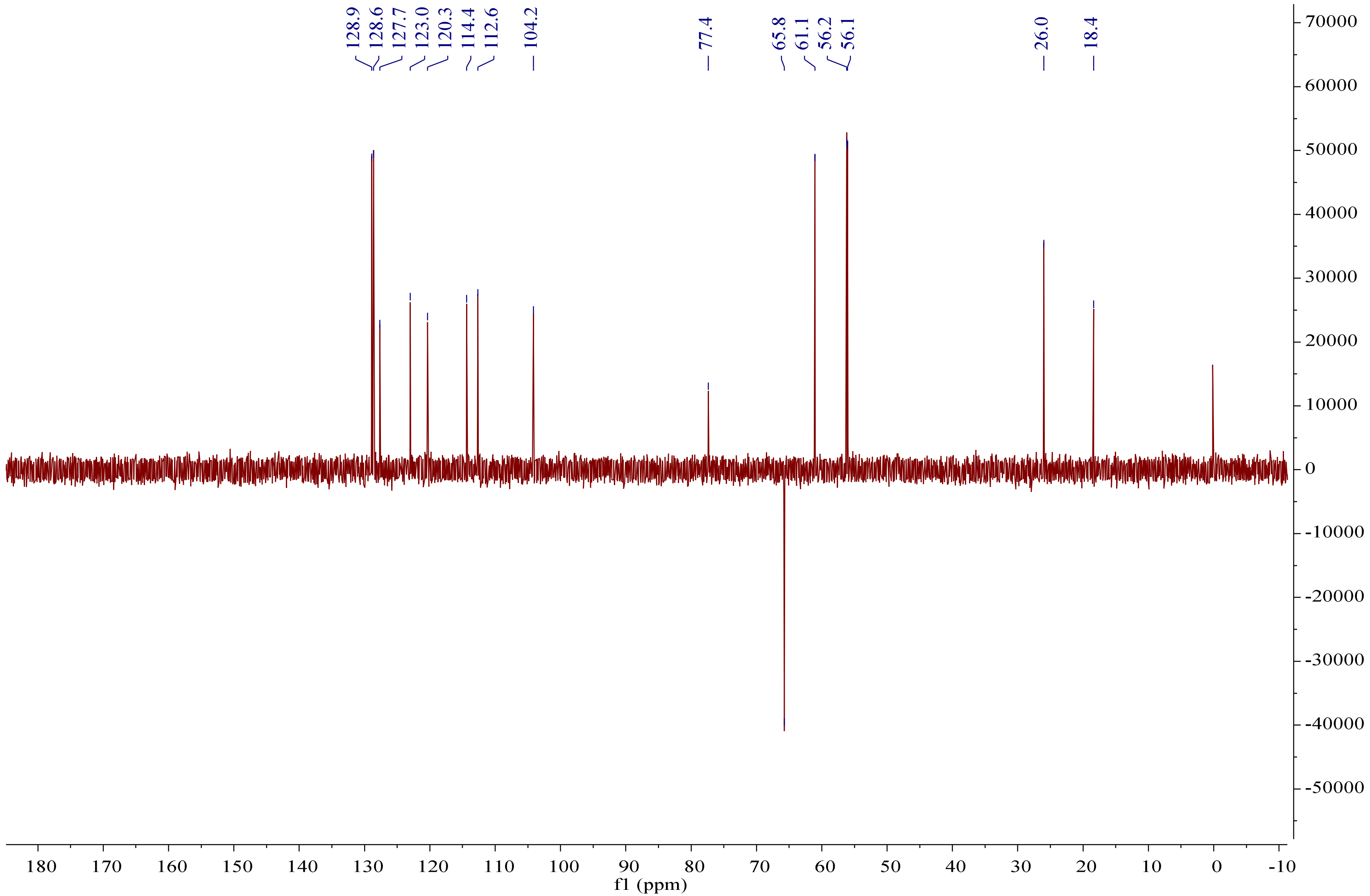

Figure S27. DEPT spectrum of **3** in $CDCl_3$ at 125 MHz

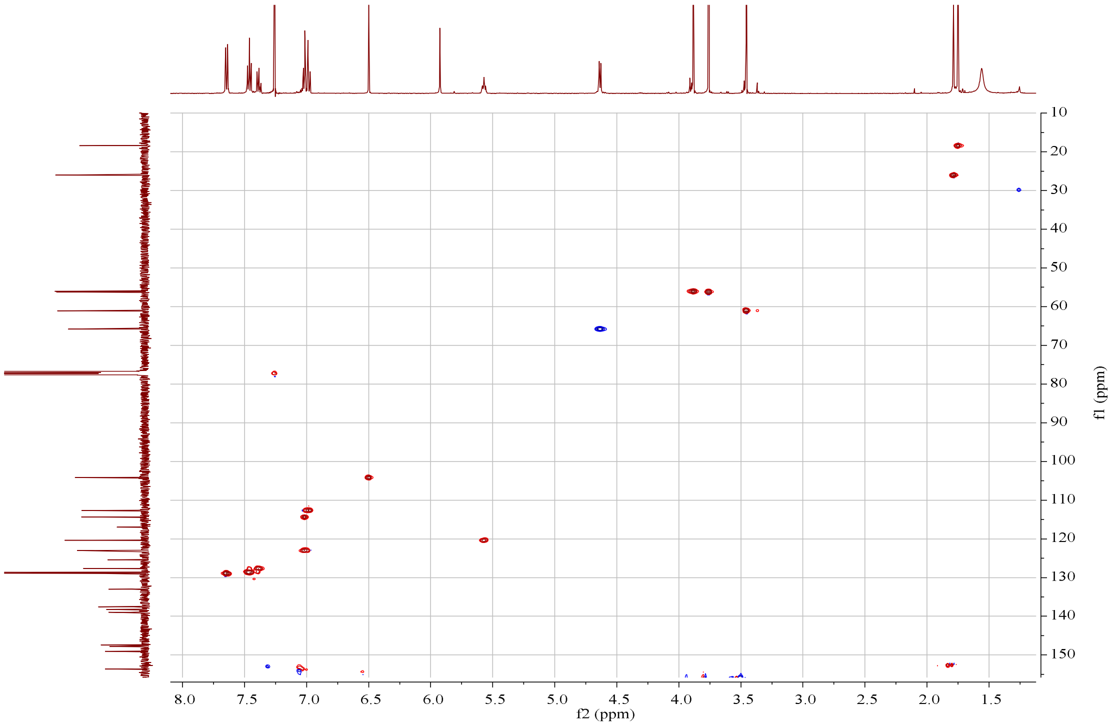

Figure S28. HSQC spectrum of **3** in $CDCl_3$

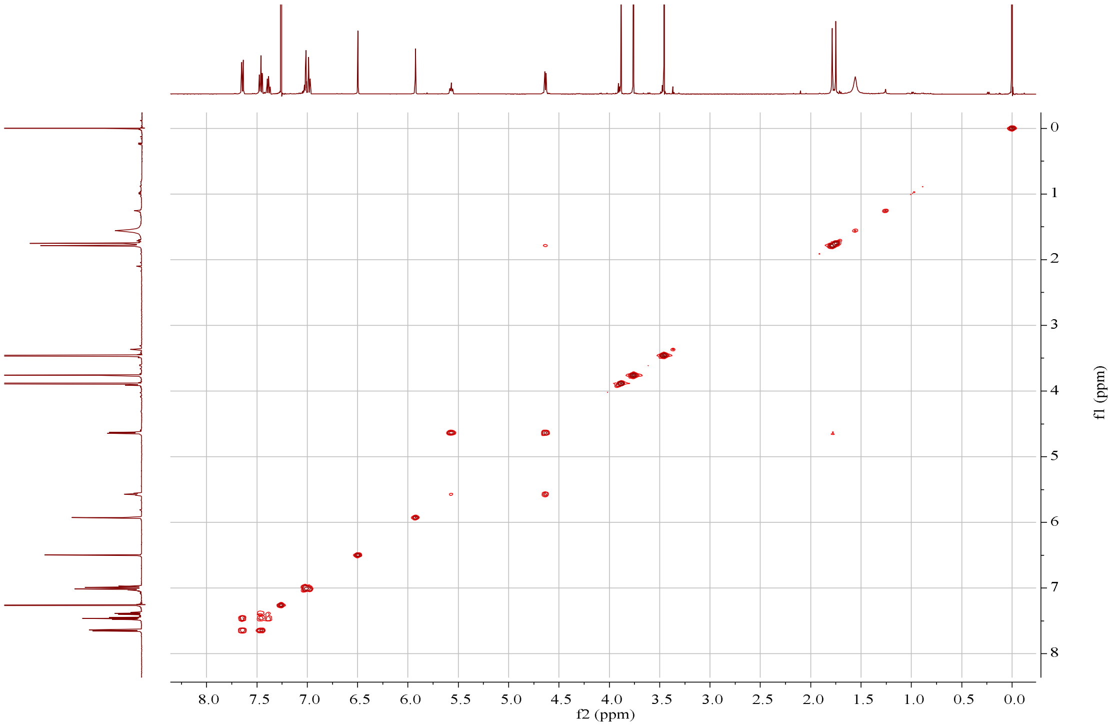

Figure S29. COSY spectrum of **3** in $CDCl_3$

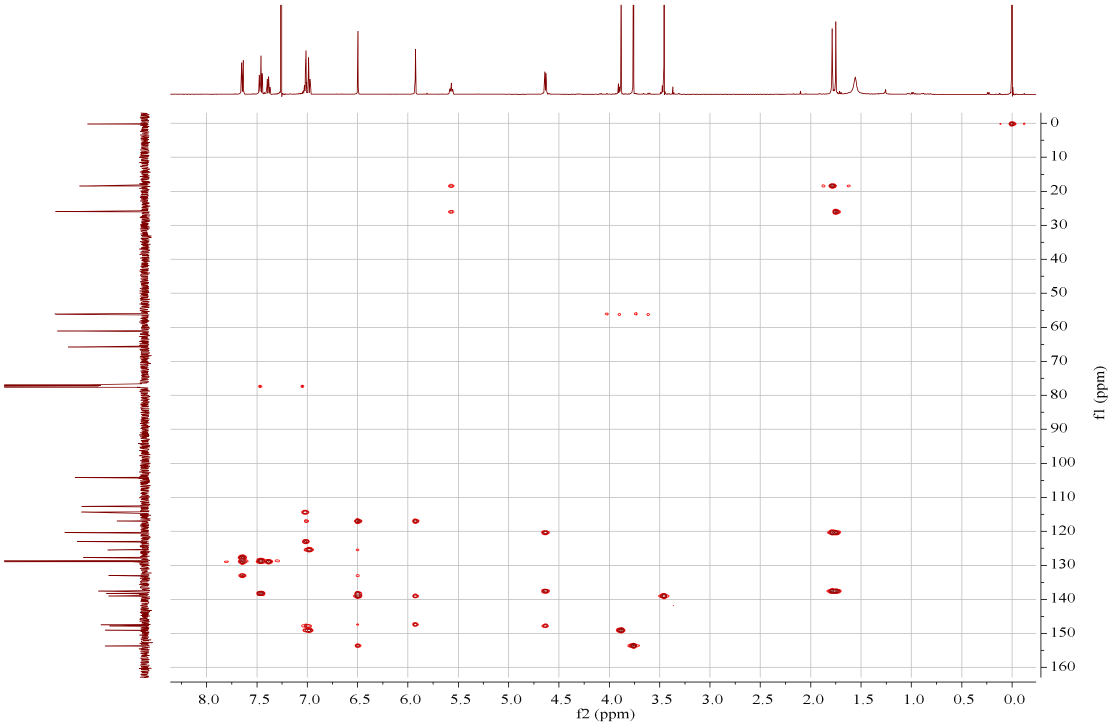

Figure S30. HMBC spectrum of **3** in $CDCl_3$

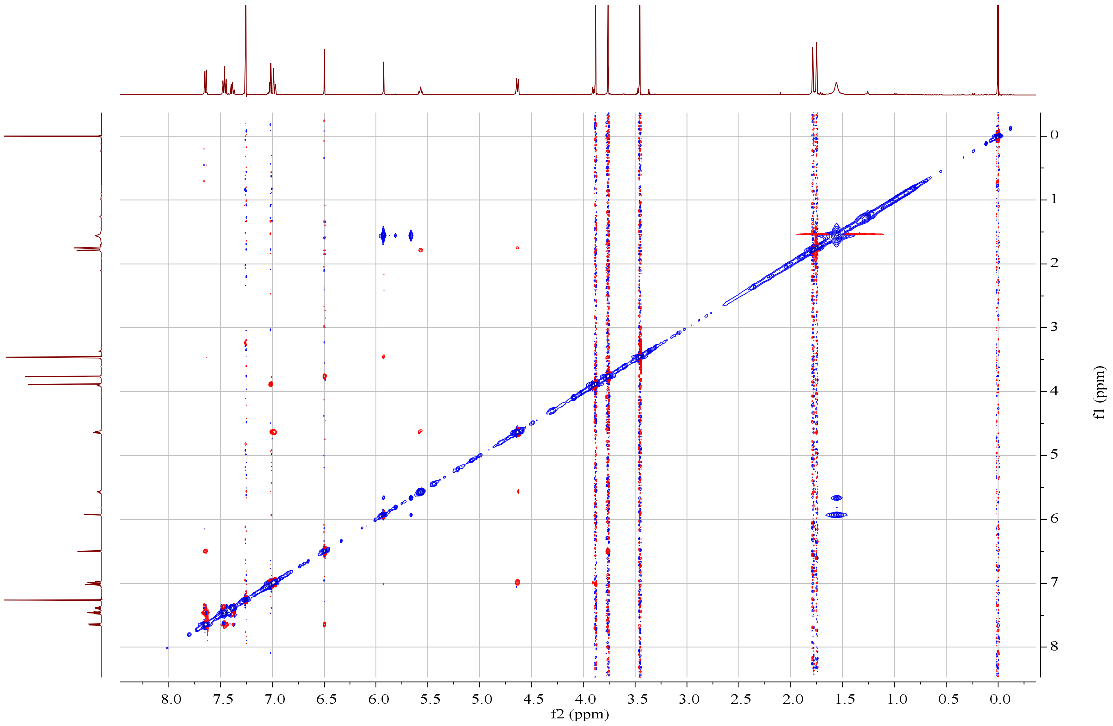

Figure S31. NOESY spectrum of **3** in $CDCl_3$

User Spectra

Fragmentor Voltage	Collision Energy	Ionization Mode
120	0	ESI

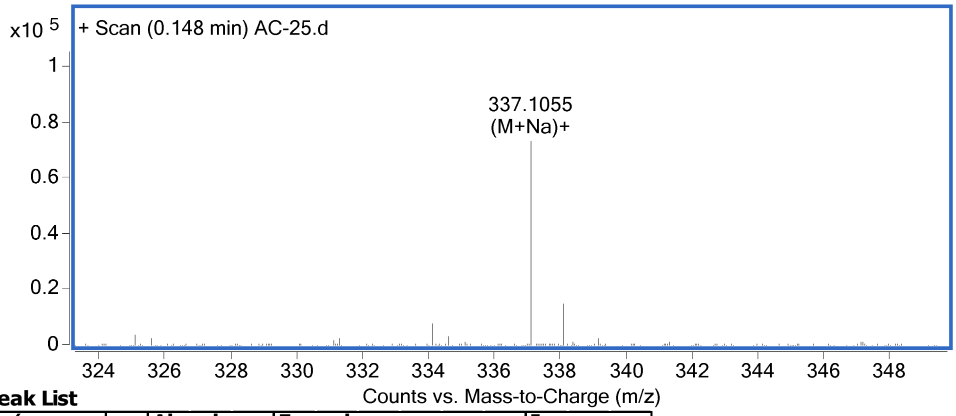

Peak List

m/z	z	Abund	Formula	Ion
125.9862		106224.1		
297.1136	1	424845.8		
298.1158	1	106297.5		
337.1055		73342.9	C18 H18 Na O5	(M+Na)+

Formula Calculator Element Limits

Element	Min	Max
C	0	70
H	0	120
O	5	5
N	0	0

Formula Calculator Results

Formula	Best	Mass	Tgt Mass	Diff (ppm)	Ion Species	Score
C18 H18 O5	TRUE	314.1163	314.1154	-2.84	C18 H18 Na O5	96.85

Figure S32. MS of **17**

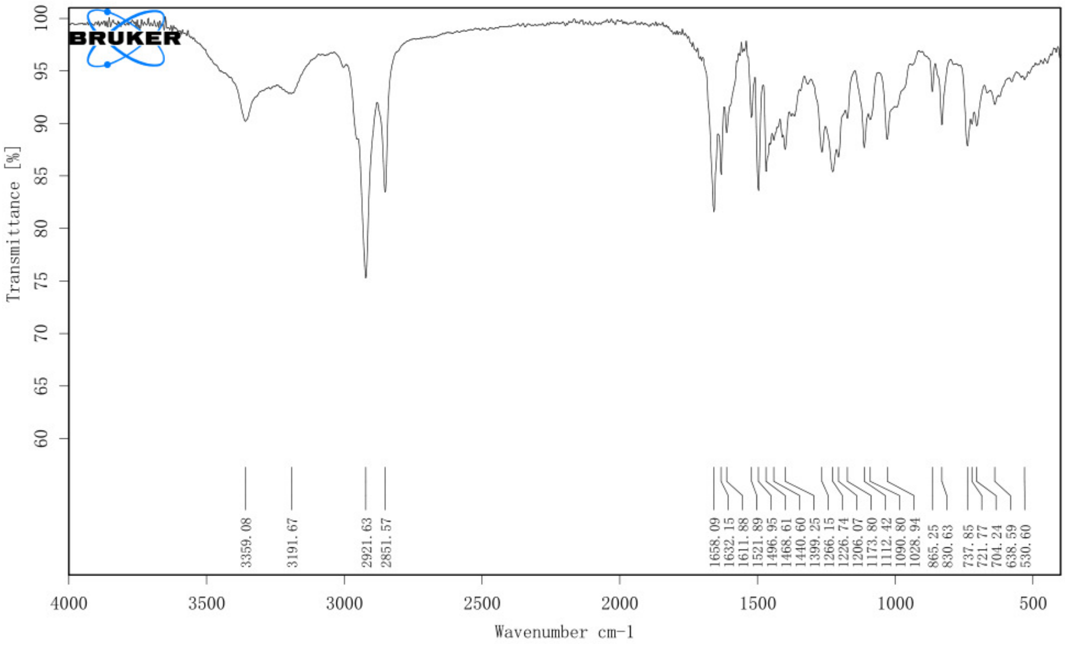

Figure S33. IR (film) of **17**

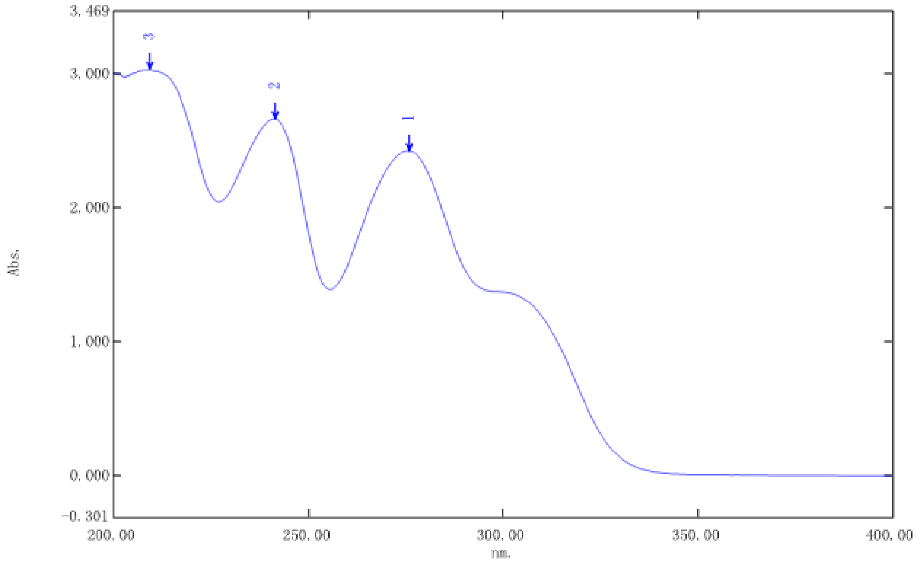

< 峰值检测表 >

No.	P/V	波长(nm)	Abs.	描述
1	↑	276.00	2.429	
2	↑	241.60	2.666	
3	↑	209.20	3.035	
4	↓	255.60	1.393	
5	↓	227.20	2.048	
6	↓	202.80	2.975	

Figure S34. UV of **17** in MeCN

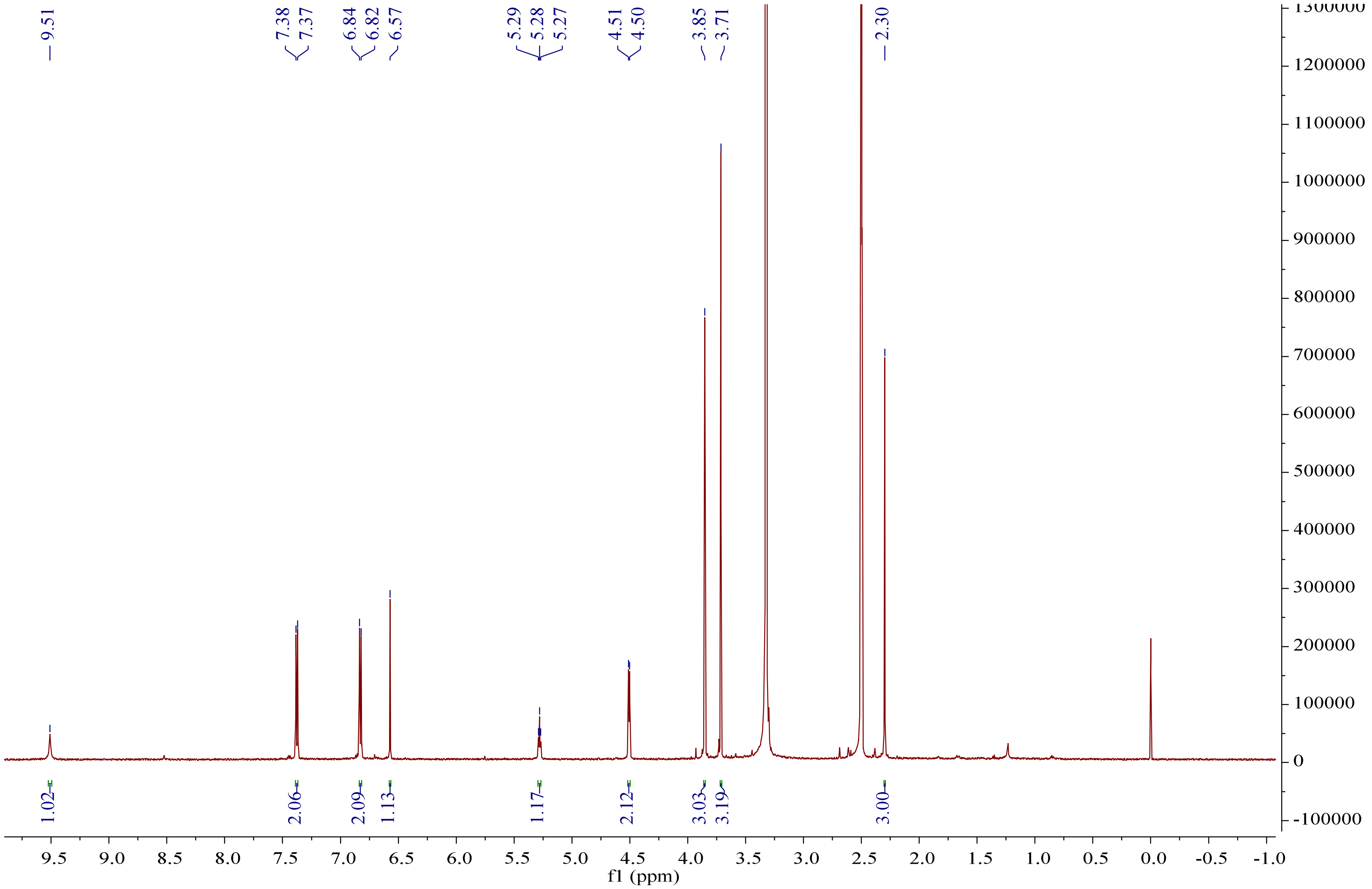

Figure S35. ^{1}H-NMR spectrum of **17** in DMSO at 600 MHz

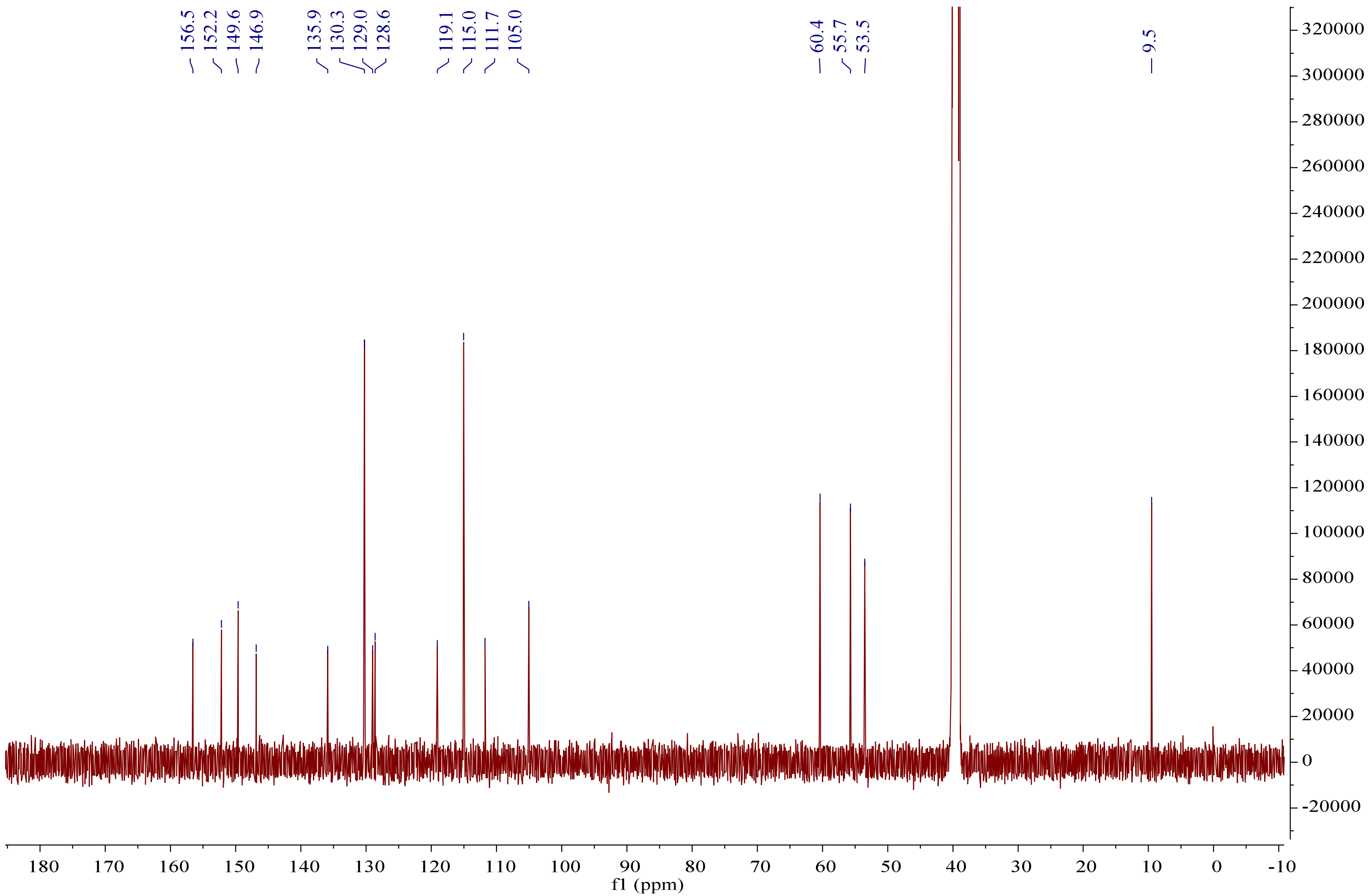

Figure S36. ^{13}C-NMR spectrum of **17** in DMSO at 125 MHz

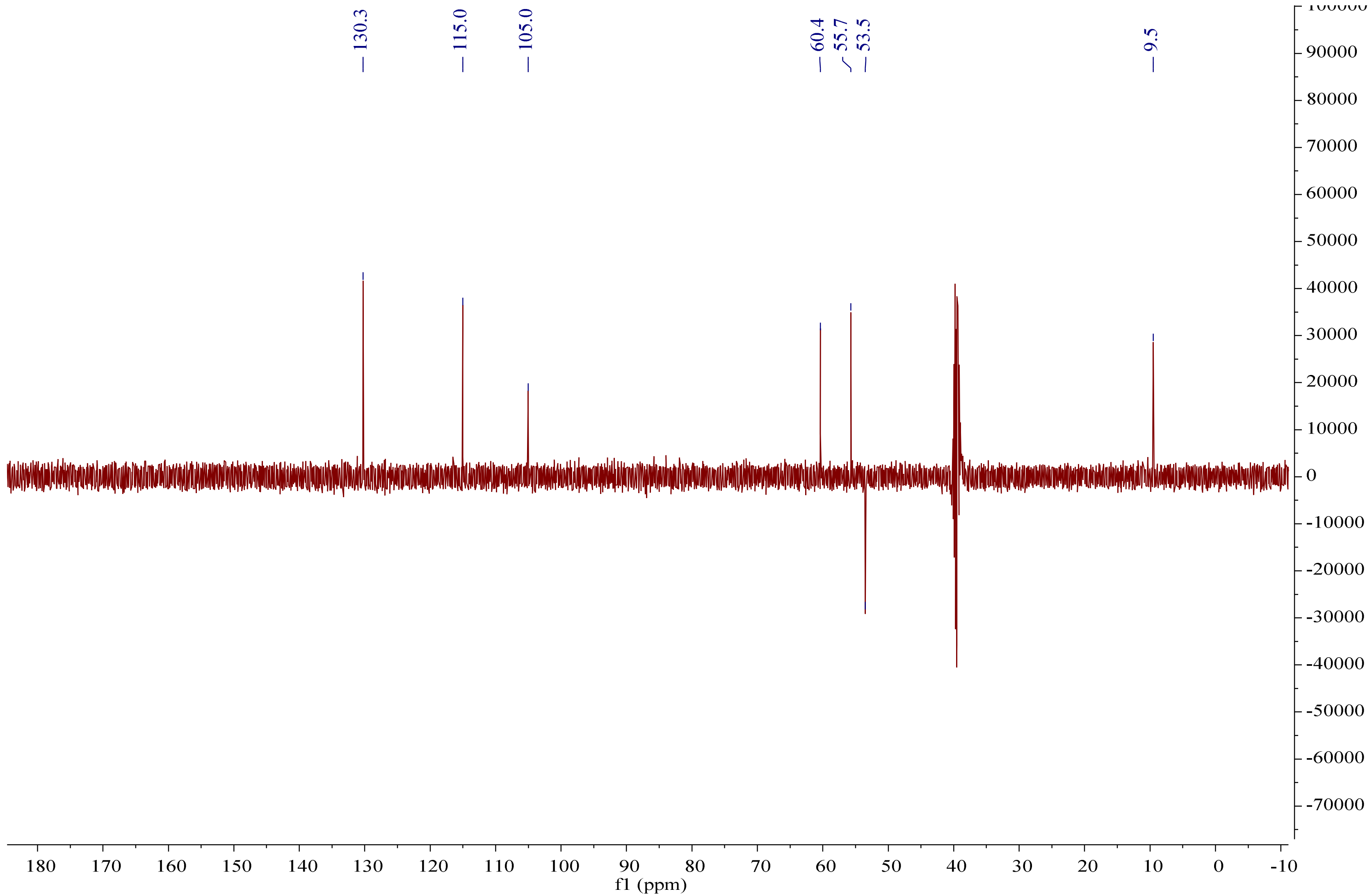

Figure S37. DEPT spectrum of **17** in DMSO at 125 MHz

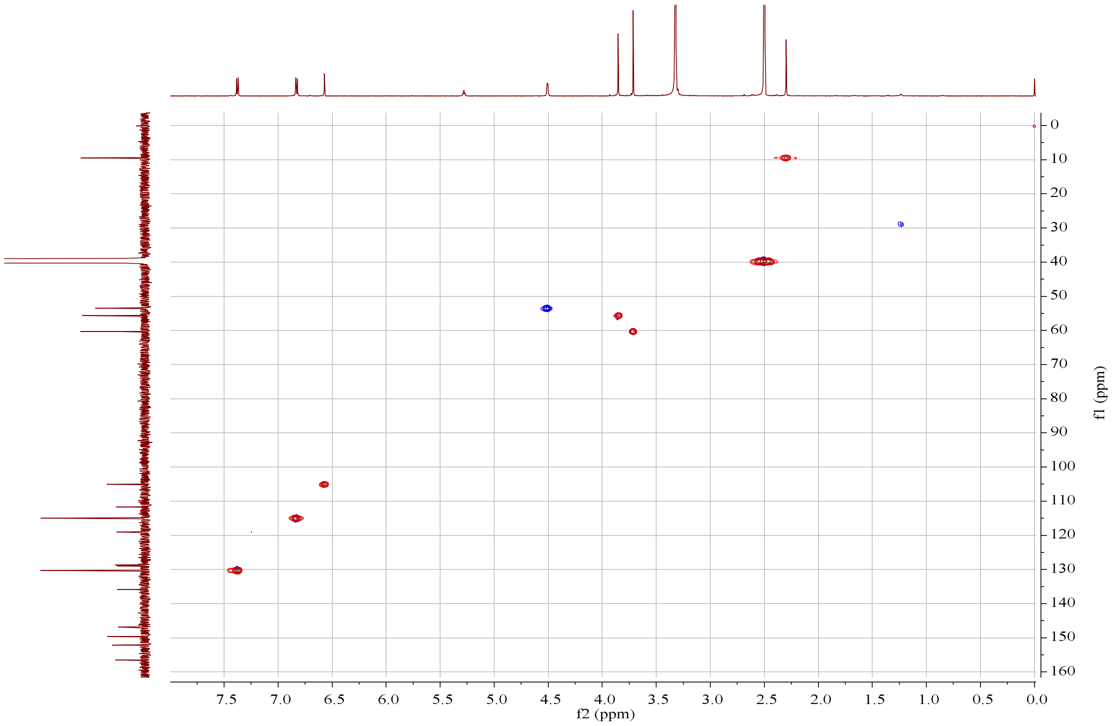

Figure S38. HSQC spectrum of **17** in DMSO

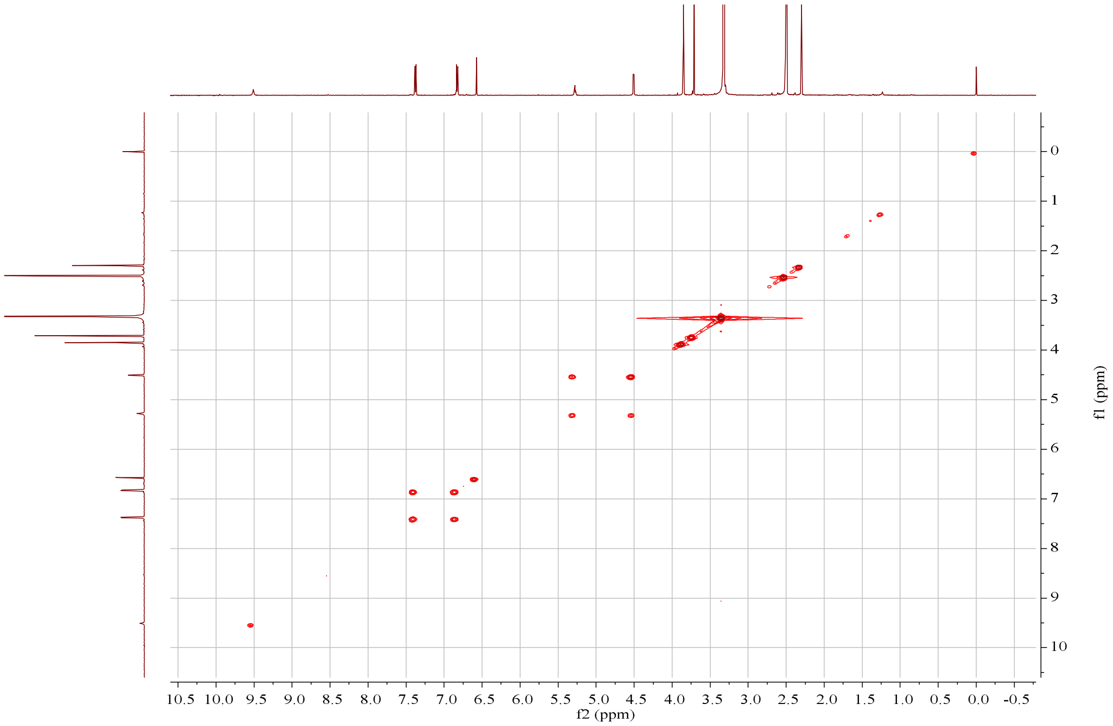

Figure S39. COSY spectrum of **17** in DMSO

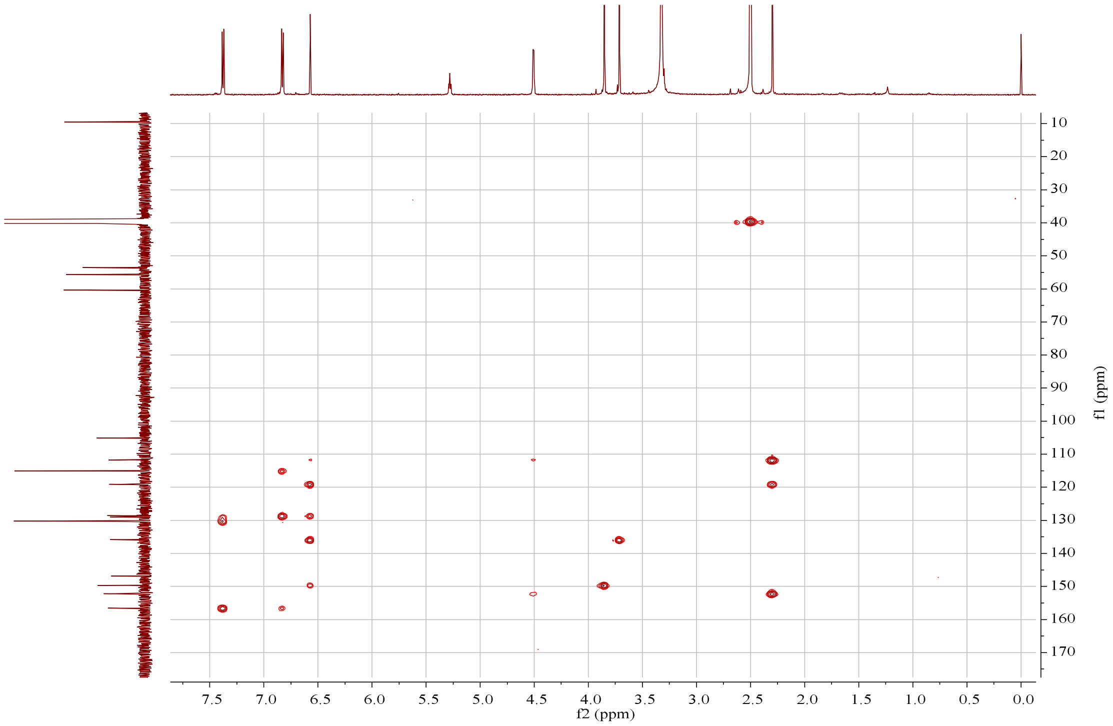

Figure S40. HMBC spectrum of **17** in DMSO

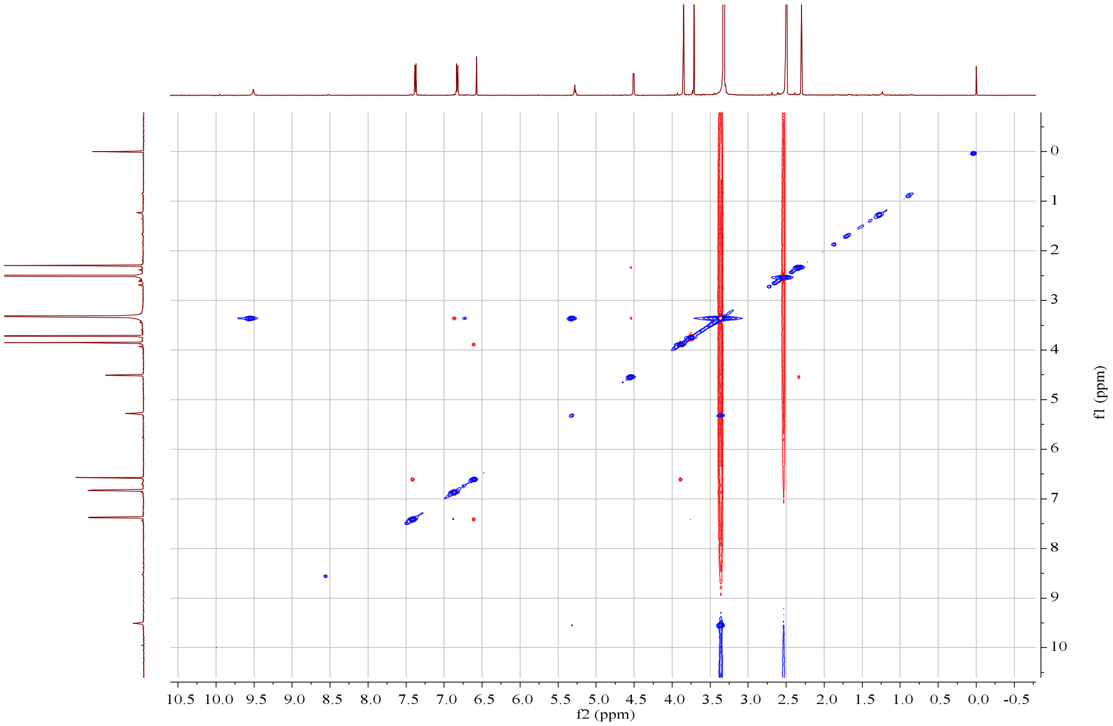

Figure S41. NOESY spectrum of **17** in DMSO

User Spectra

Fragmentor Voltage	Collision Energy	Ionization Mode
120	0	ESI

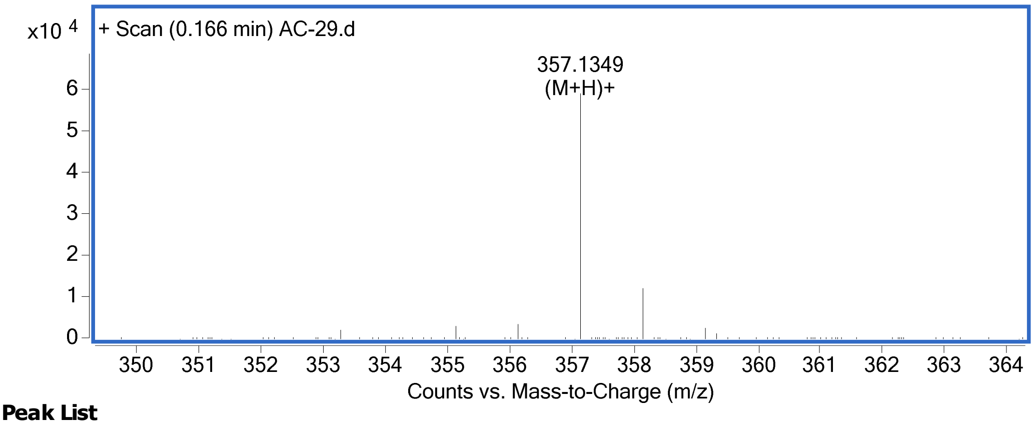

Peak List

m/z	Abund	Formula	Ion
125.9866	44687.1		
357.1349	59318	C20 H21 O6	(M+H)+
374.1614	53866.2		
379.117	46411.6		

Formula Calculator Element Limits

Element	Min	Max
C	0	70
H	0	120
O	6	6
N	0	0

Formula Calculator Results

Formula	Best	Mass	Tgt Mass	Diff (ppm)	Ion Species	Score
C20 H20 O6	TRUE	356.1276	356.126	-4.61	C20 H21 O6	91.08

Figure S42. MS of **18**

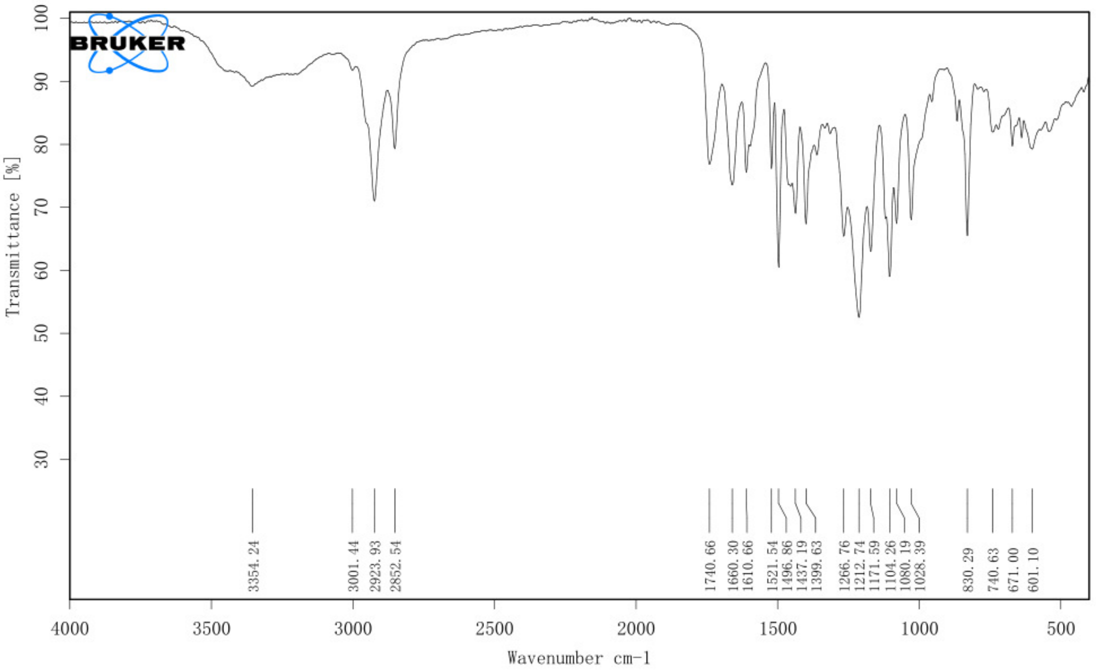

Figure S43. IR (film) of **18**

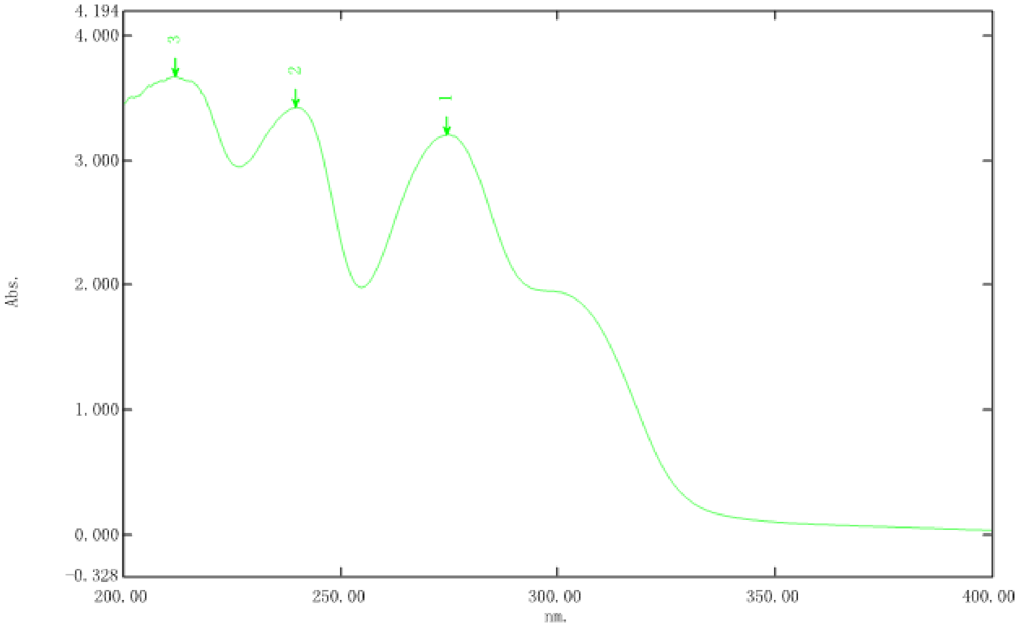

< 峰值检测表 >

No.	P/V	波长(nm)	Abs.	描述
1	⬆	274.60	3.209	
2	⬆	239.60	3.425	
3	⬆	211.80	3.674	
4	⬇	254.80	1.982	
5	⬇	226.60	2.946	

Figure S44. UV of **18** in MeCN

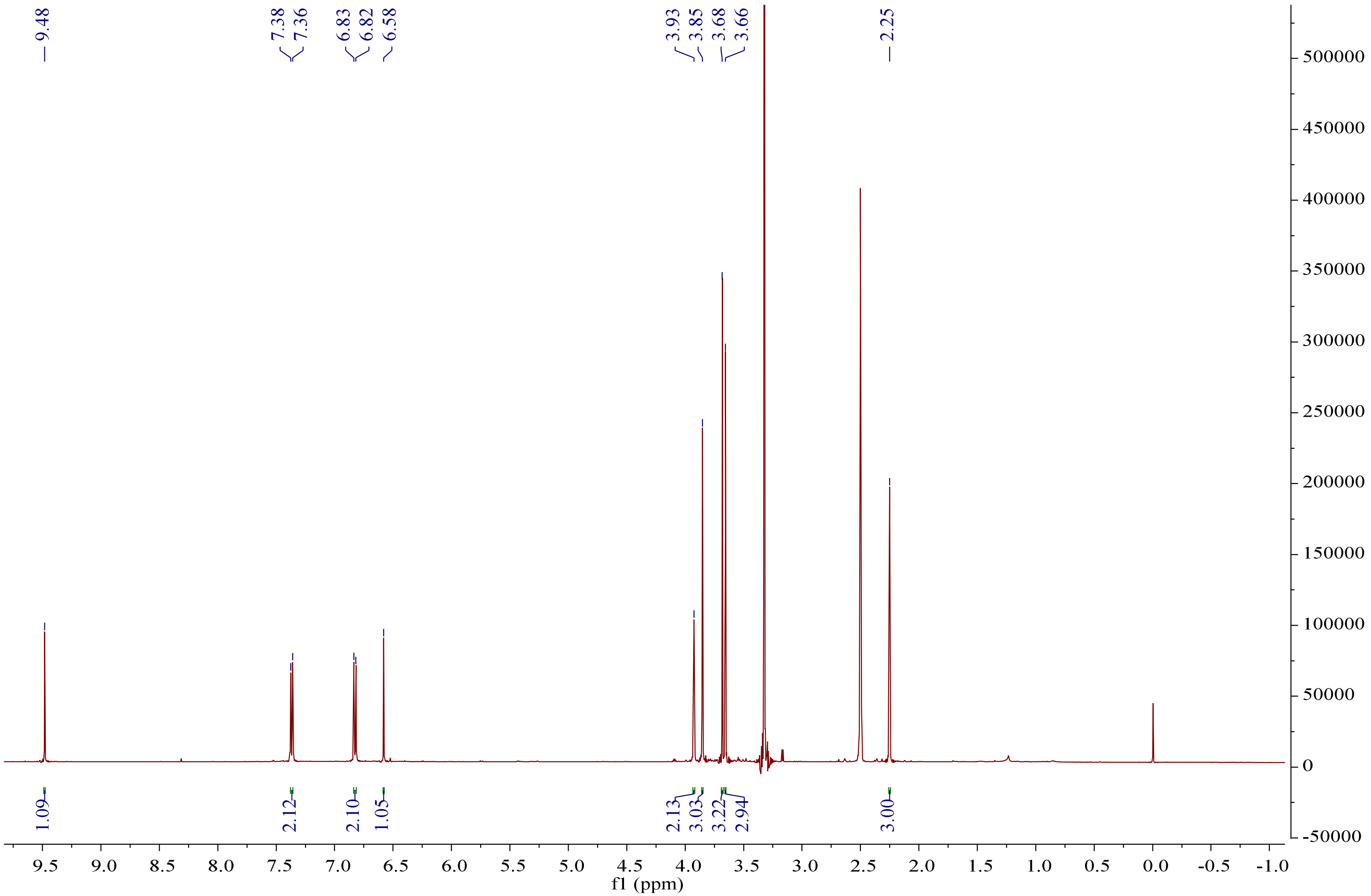

Figure S45. ^{1}H-NMR spectrum of **18** in DMSO at 500 MHz

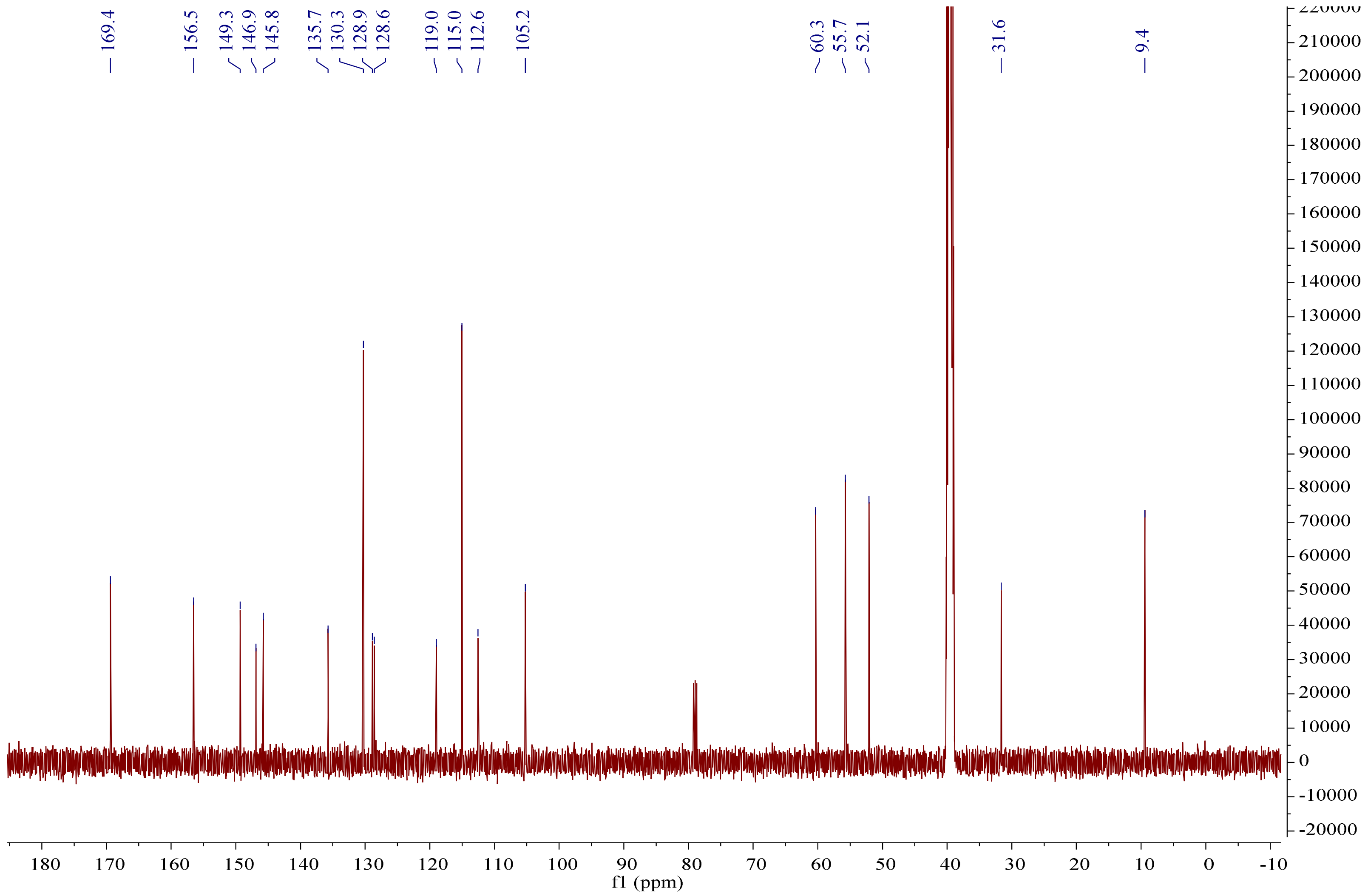

Figure S46. ^{13}C-NMR spectrum of **18** in DMSO at 125 MHz

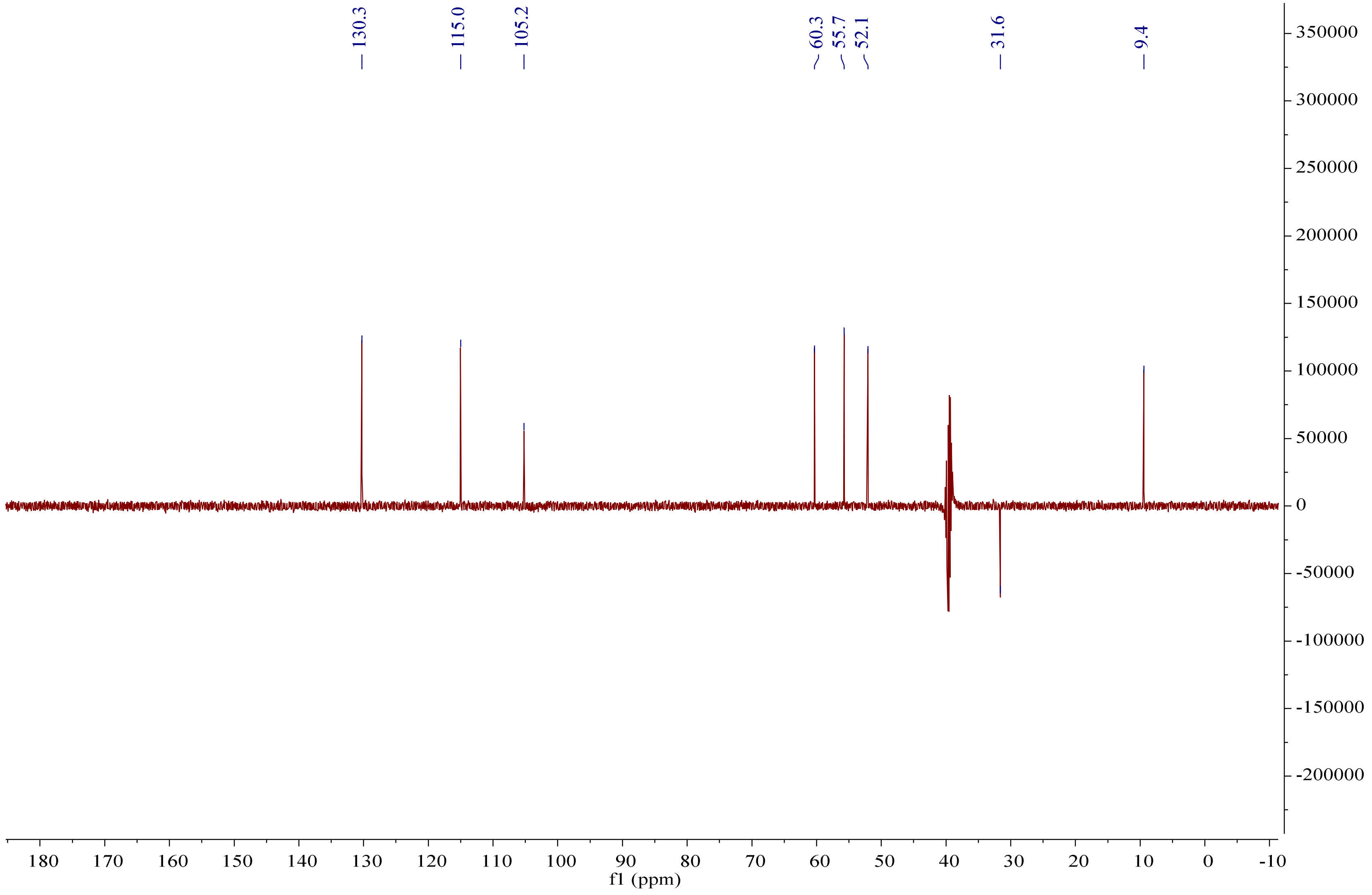

Figure S47. DEPT spectrum of **18** in DMSO at 125 MHz

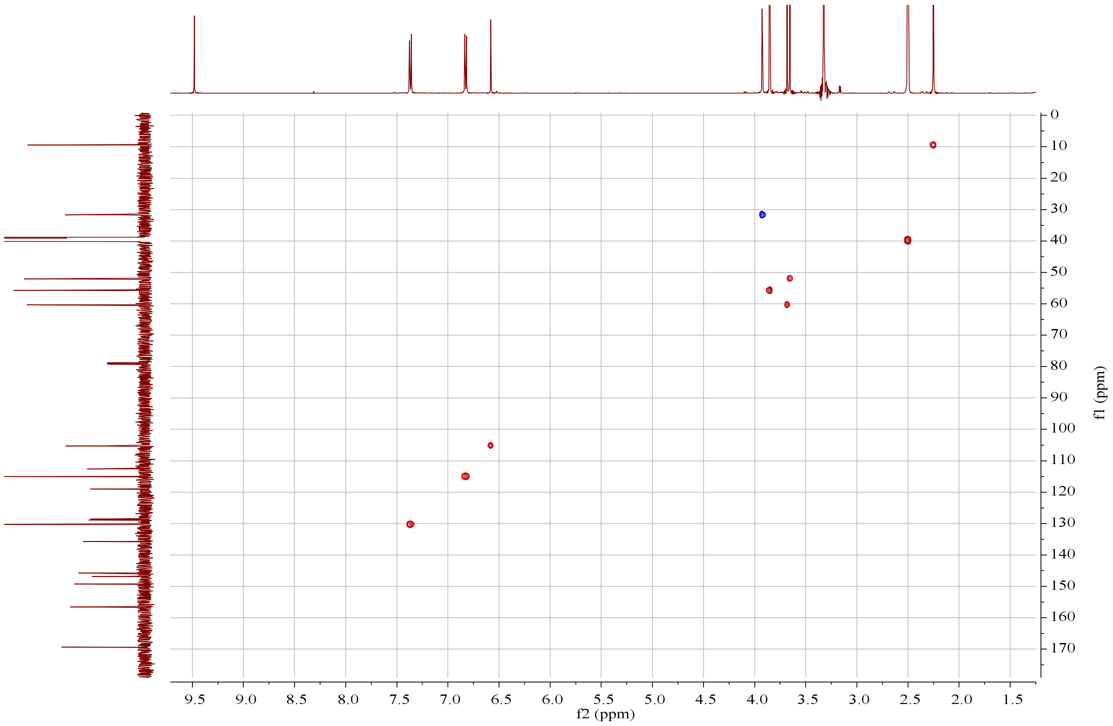

Figure S48. HSQC spectrum of **18** in DMSO

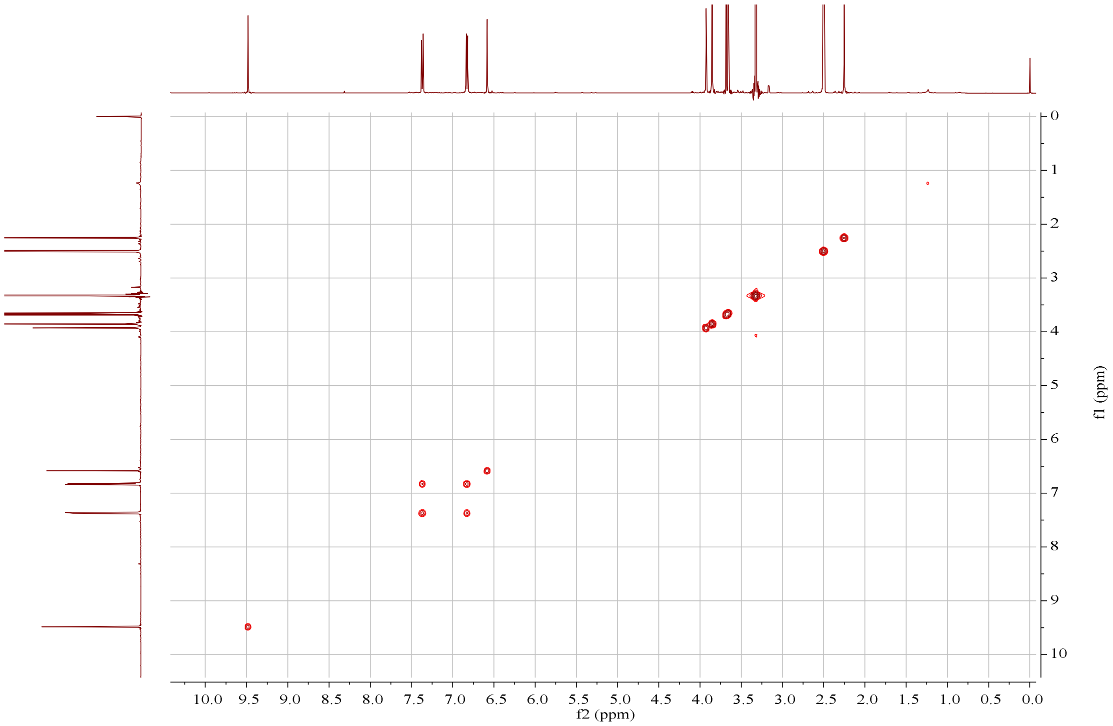

Figure S49. COSY spectrum of **18** in DMSO

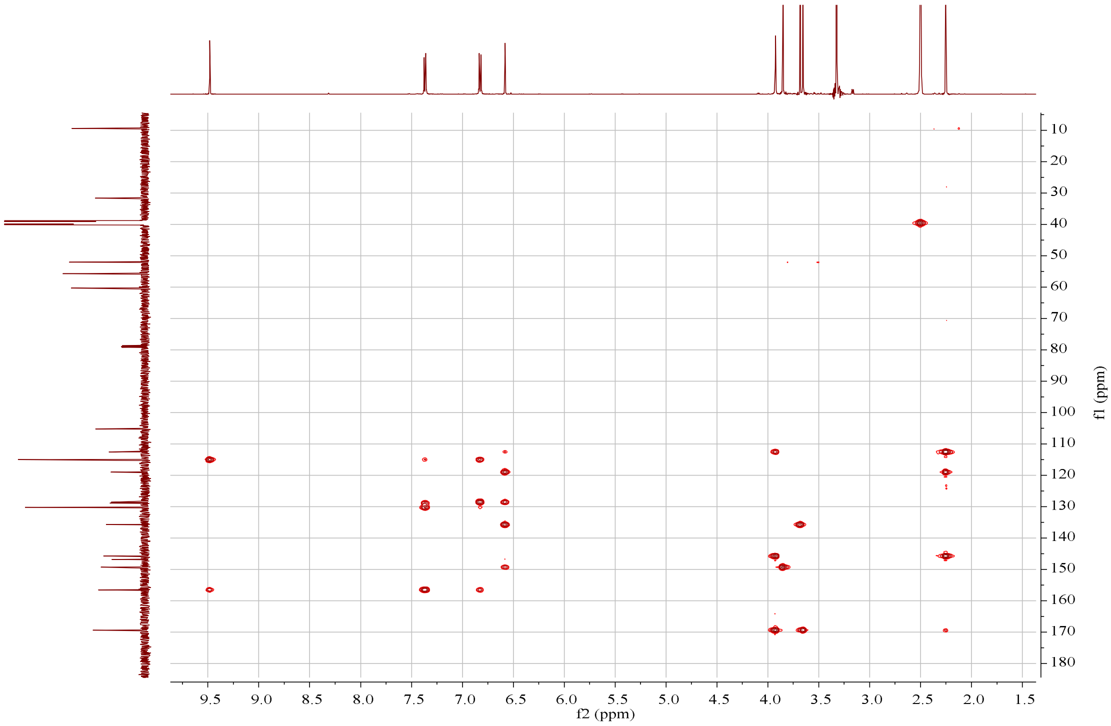

Figure S50. HMBC spectrum of **18** in DMSO

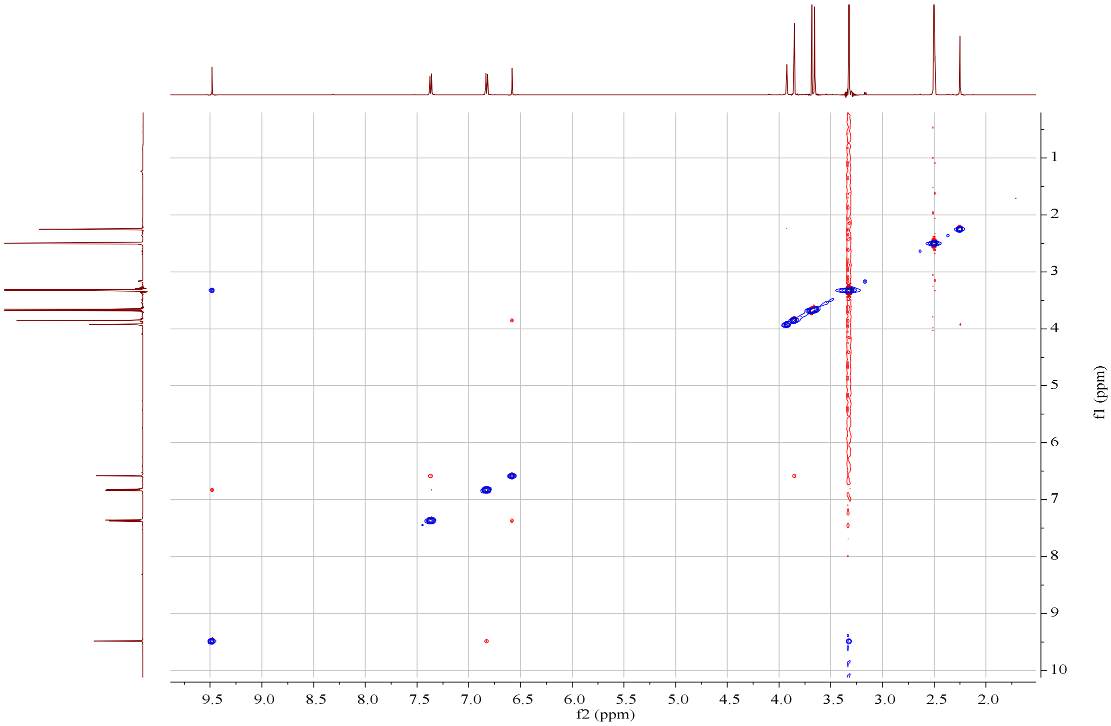

Figure S51. NOESY spectrum of **18** in DMSO

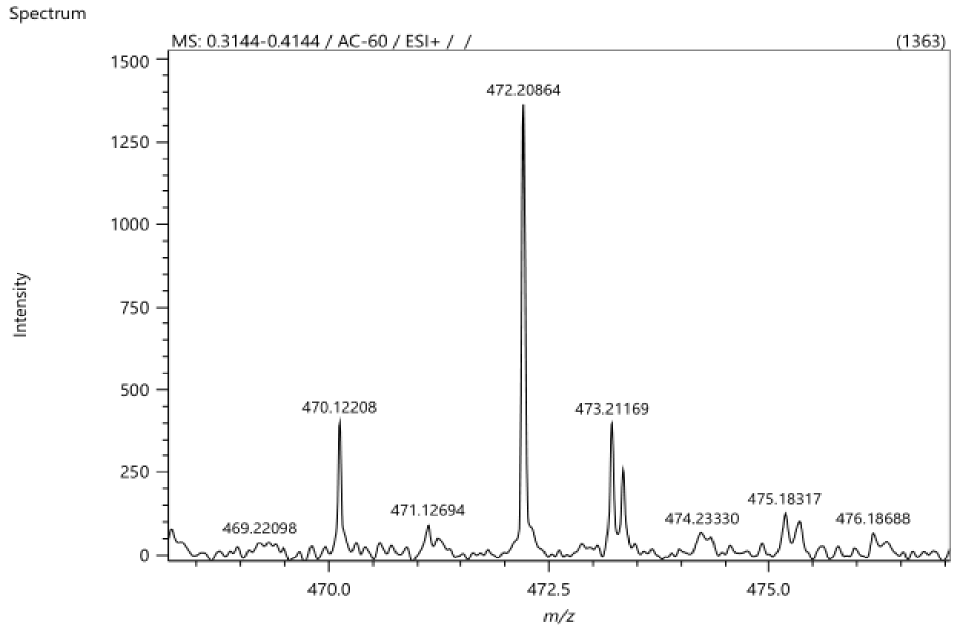

Elemental Composition

Parameters	
Tolerance:	±5.00 ppm
Electron:	Odd/Even
Charge:	+1
DBE:	-1.5 - 200.0

Elements Set 1:

Symbol	C	H	N	O	Na	S	Cl	Br
Min	0	0	0	0	1	0	0	0
Max	200	120	3	8	1	0	0	0

Symbol	F	Si	P	Mn	B	I	Ru
Min	0	0	0	0	0	0	0
Max	0	0	0	0	0	0	0

Results

Mass	Intensity	Intensity [%]	Formula	Calculated Mass	Mass Difference [mDa]	Mass Difference [ppm]	DBE
472.20864	1363.13	22.73	C27 H31 N O5 Na	472.20944	-0.80	-1.69	12.5
			C24 H33 O8 Na	472.20676	1.88	3.98	8.0

Figure S52. MS of **22**

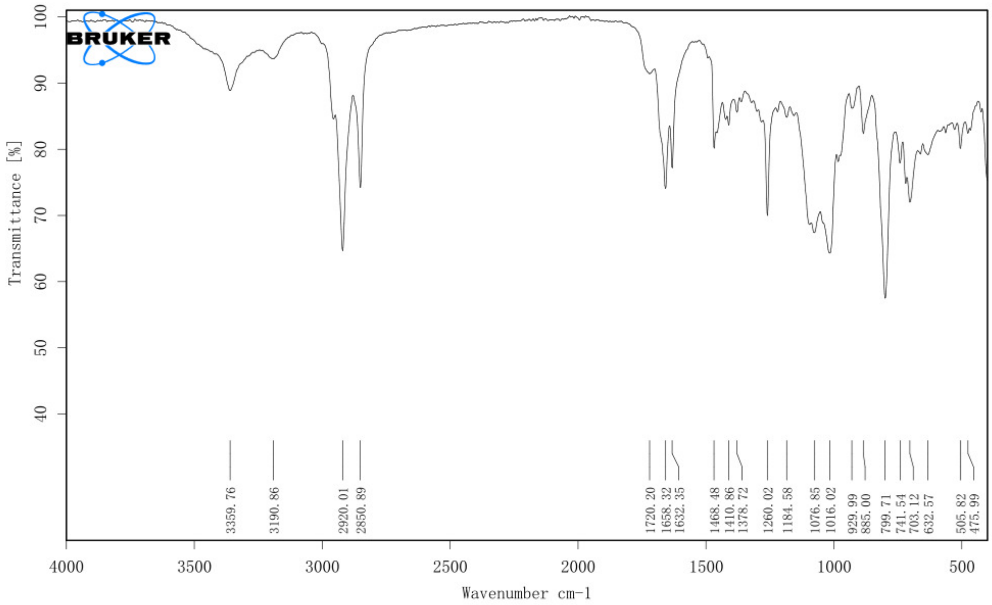

Figure S53. IR (film) of **22**

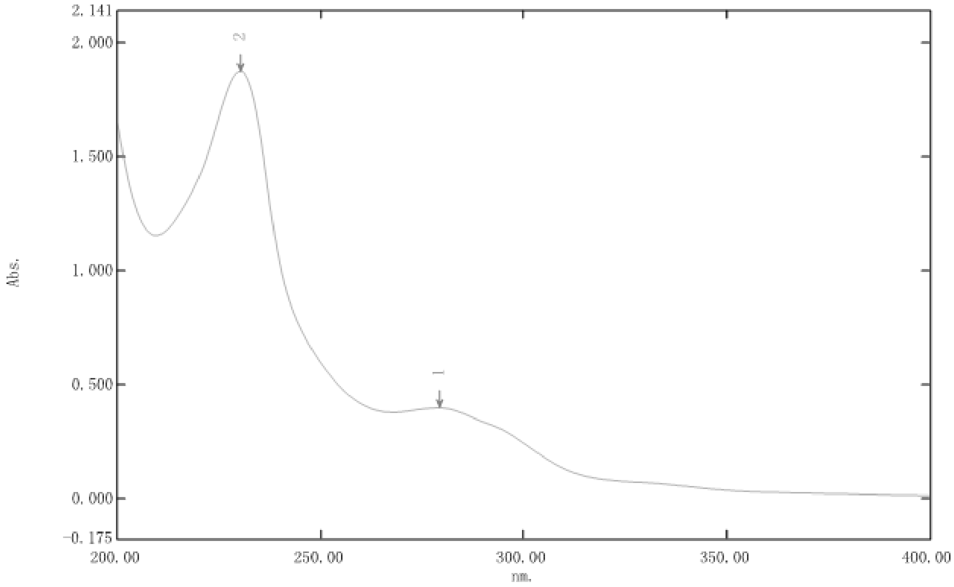

< 峰值检测表 >

No.	P/V	波长(nm)	Abs.	描述
1		279. 40	0. 397	
2		230. 40	1. 875	
3		268. 20	0. 377	
4		209. 60	1. 154	

Figure S54. UV of **22** in MeCN

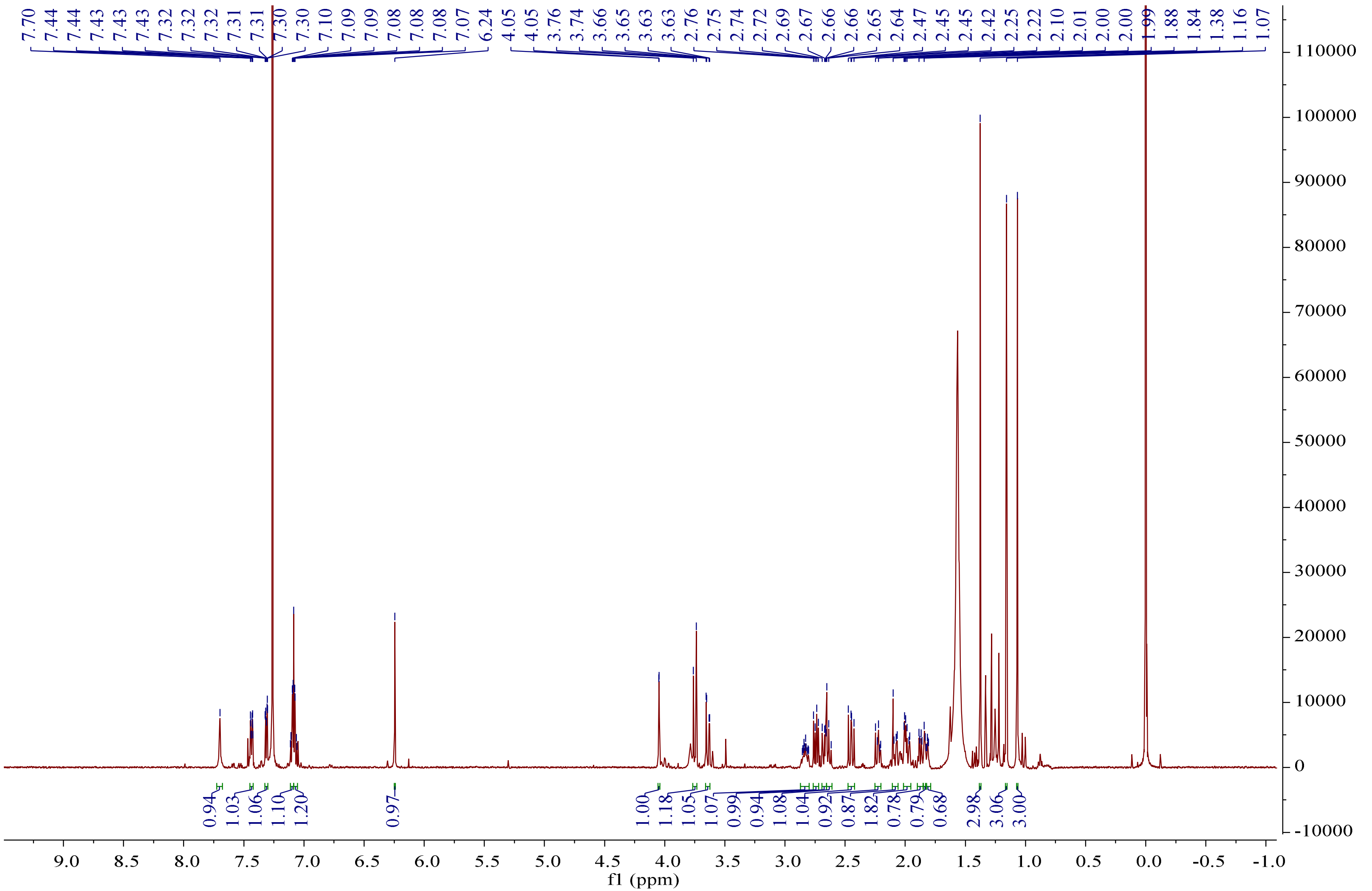

Figure S55. ^{1}H-NMR spectrum of **22** in $CDCl_3$ at 500 MHz

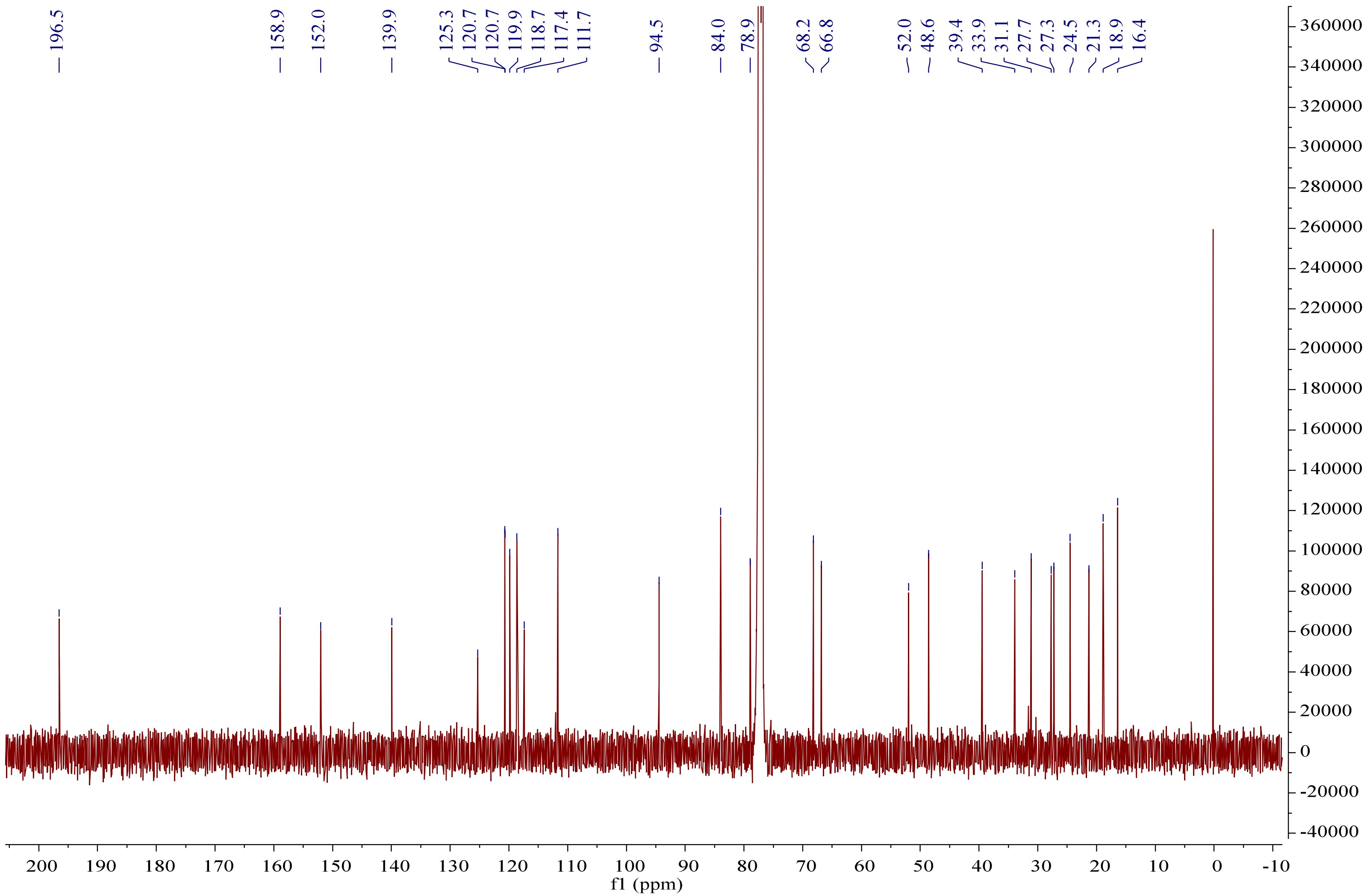

Figure S56. ^{13}C-NMR spectrum of **22** in $CDCl_3$ at 125 MHz

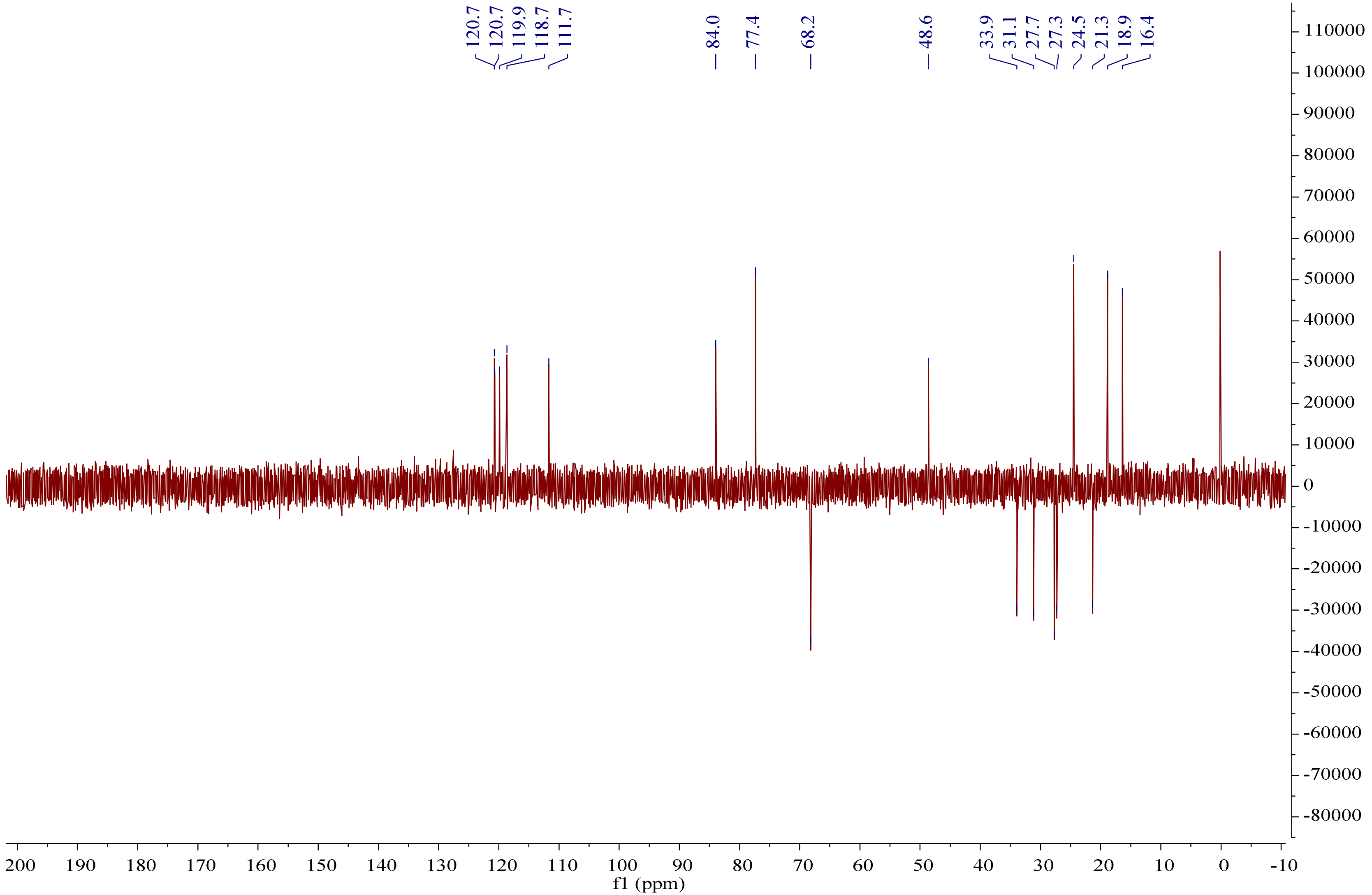

Figure S57. DEPT spectrum of **22** in $CDCl_3$ at 125 MHz

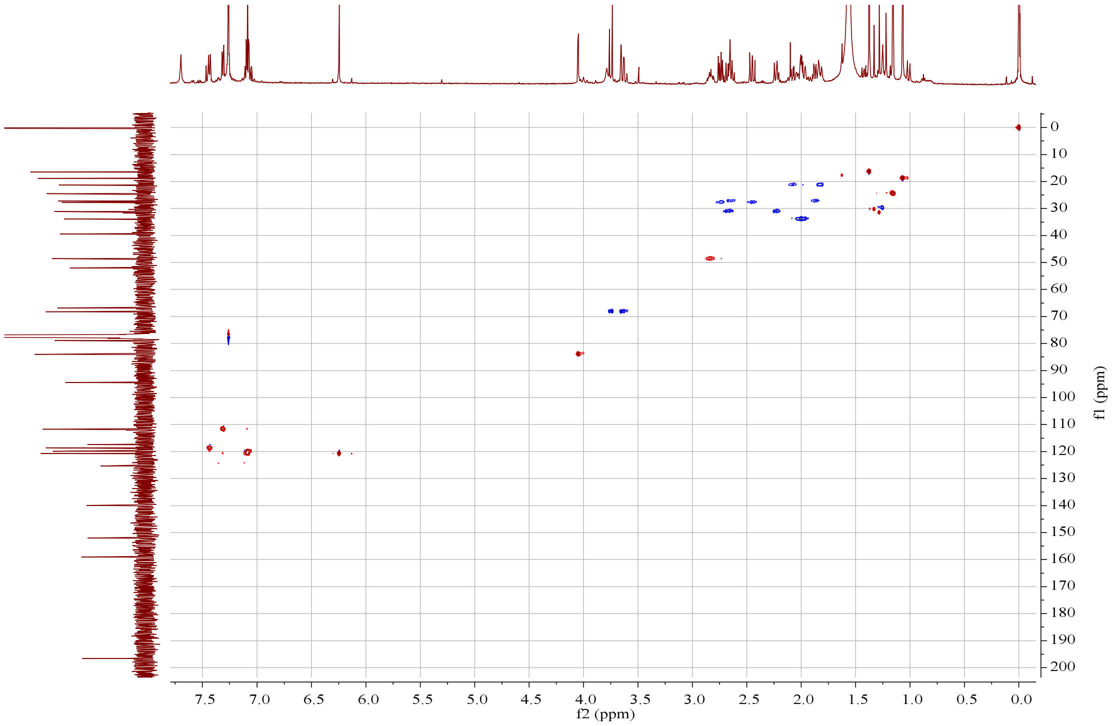

Figure S58. HSQC spectrum of **22** in $CDCl_3$

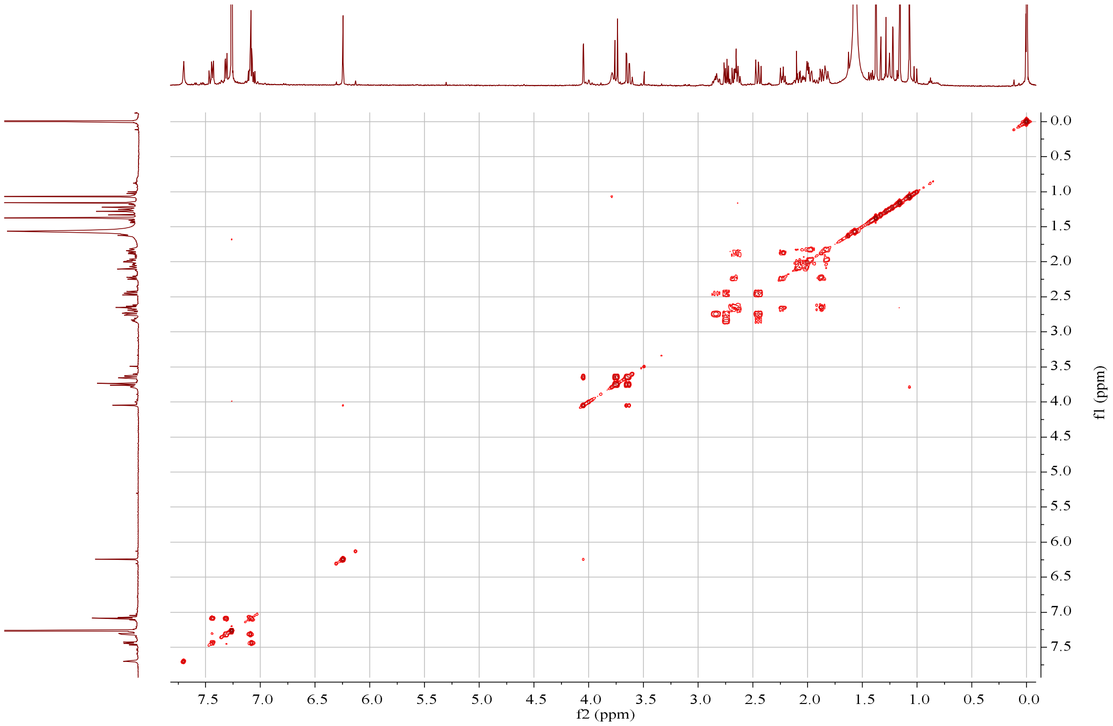

Figure S59. COSY spectrum of **22** in $CDCl_3$

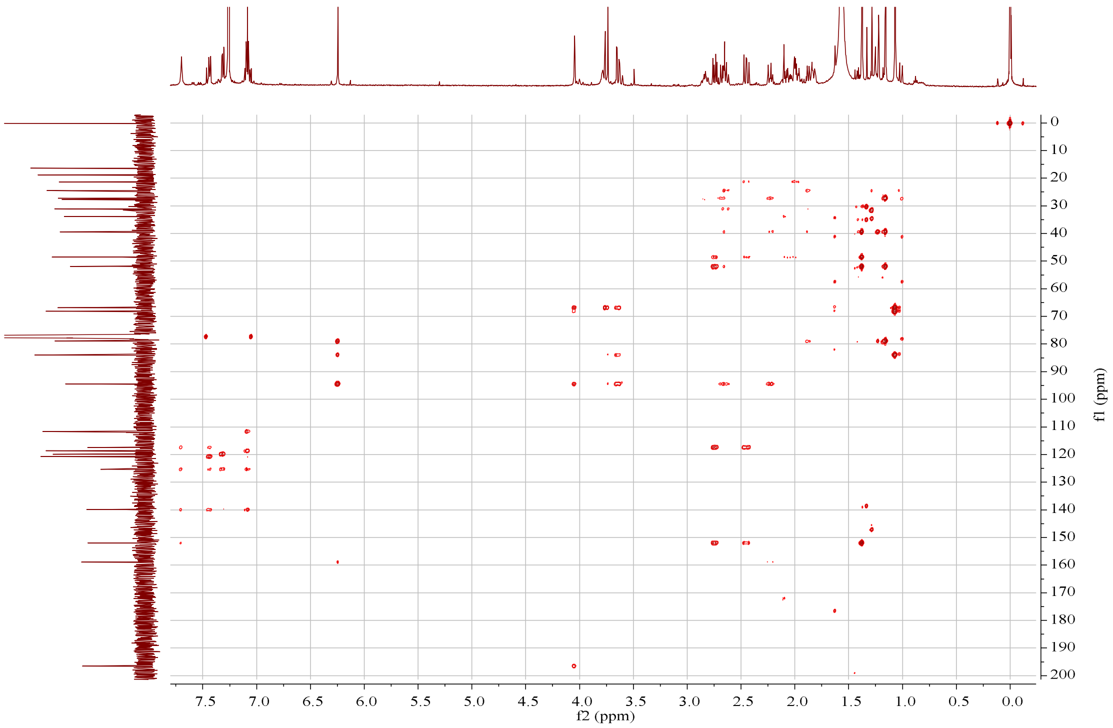

Figure S60. HMBC spectrum of **22** in $CDCl_3$

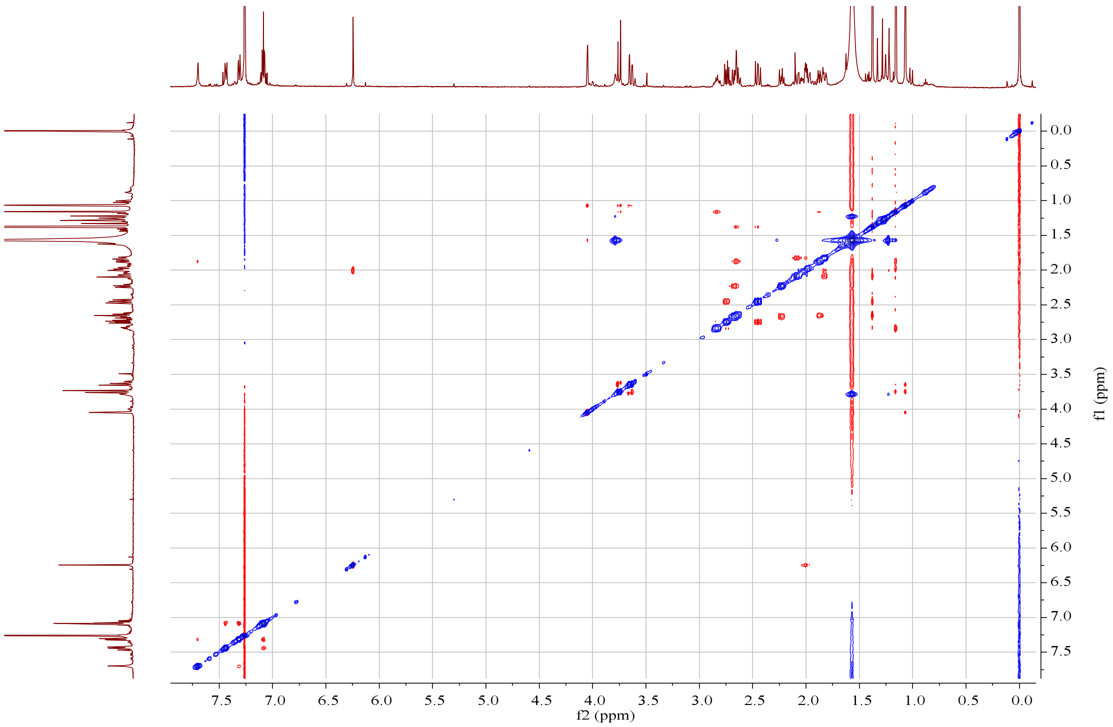

Figure S61. NOESY spectrum of **22** in $CDCl_3$

User Spectra

Fragmentor Voltage	Collision Energy	Ionization Mode
120	0	ESI

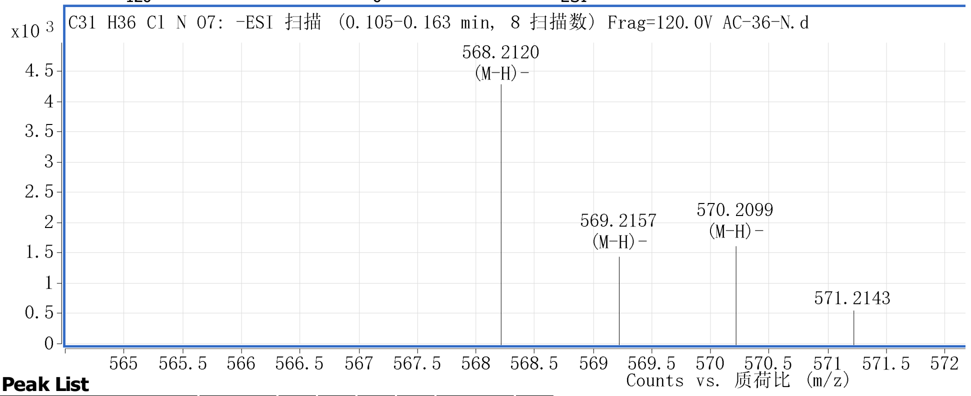

Peak List

m/z	Abund	Formula	Ion
568.2120	4293.03	C31 H35 N O7 Cl	(M - H)-
569.2157	1451.82		
570.2099	1625.9		
571.2143	573.73		

Formula Calculator Element Limits

Element	Min	Max
C	0	70
H	0	120
Cl	0	3
O	0	30
N	0	30

Formula Calculator Results

Formula	Best	Mass	Tgt Mass	Diff (ppm)	Ion Species	Score
C31 H36 N O7 Cl	TRUE	569.218	569.2157	-2.8	C31 H35 N O7 Cl	81.77

Figure S62. MS of **23**

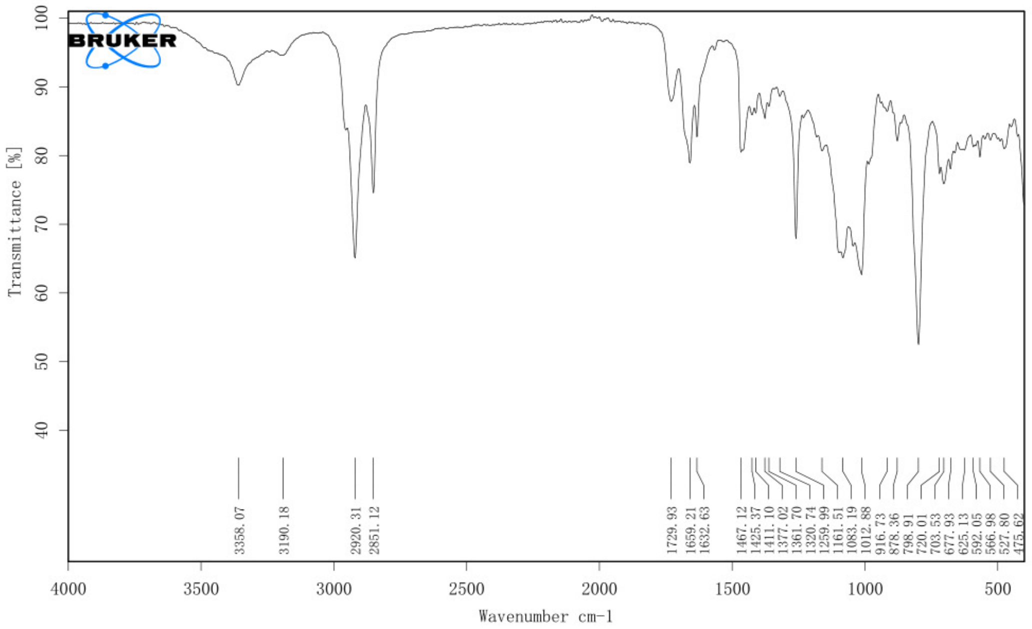

Figure S63. IR (film) of **23**

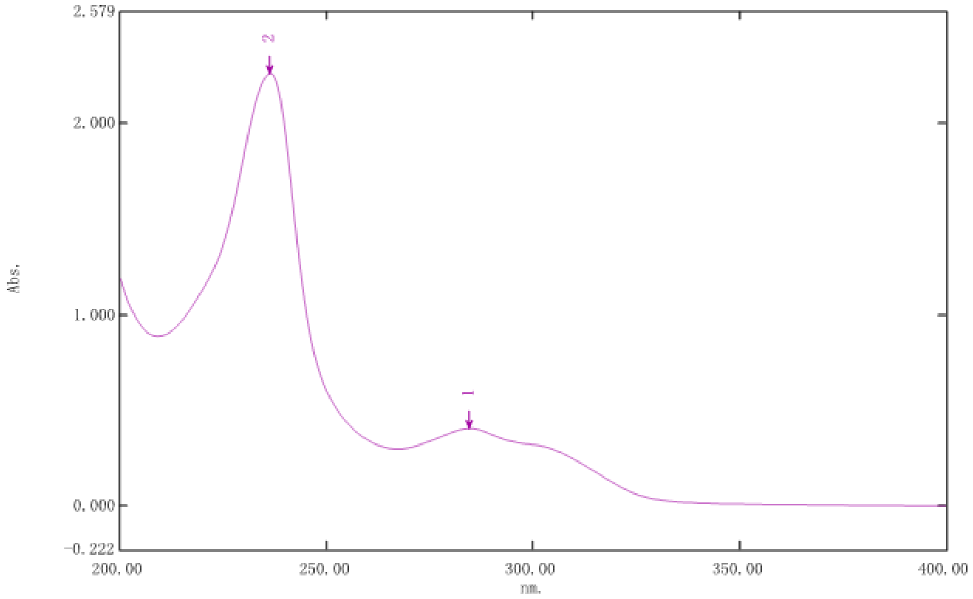

< 峰值检测表 >

No.	P/V	波长(nm)	Abs.	描述
1	↑	284.80	0.407	
2	↑	236.40	2.256	
3	↓	267.40	0.300	
4	↓	209.40	0.887	

Figure S64. UV of **23** in MeCN

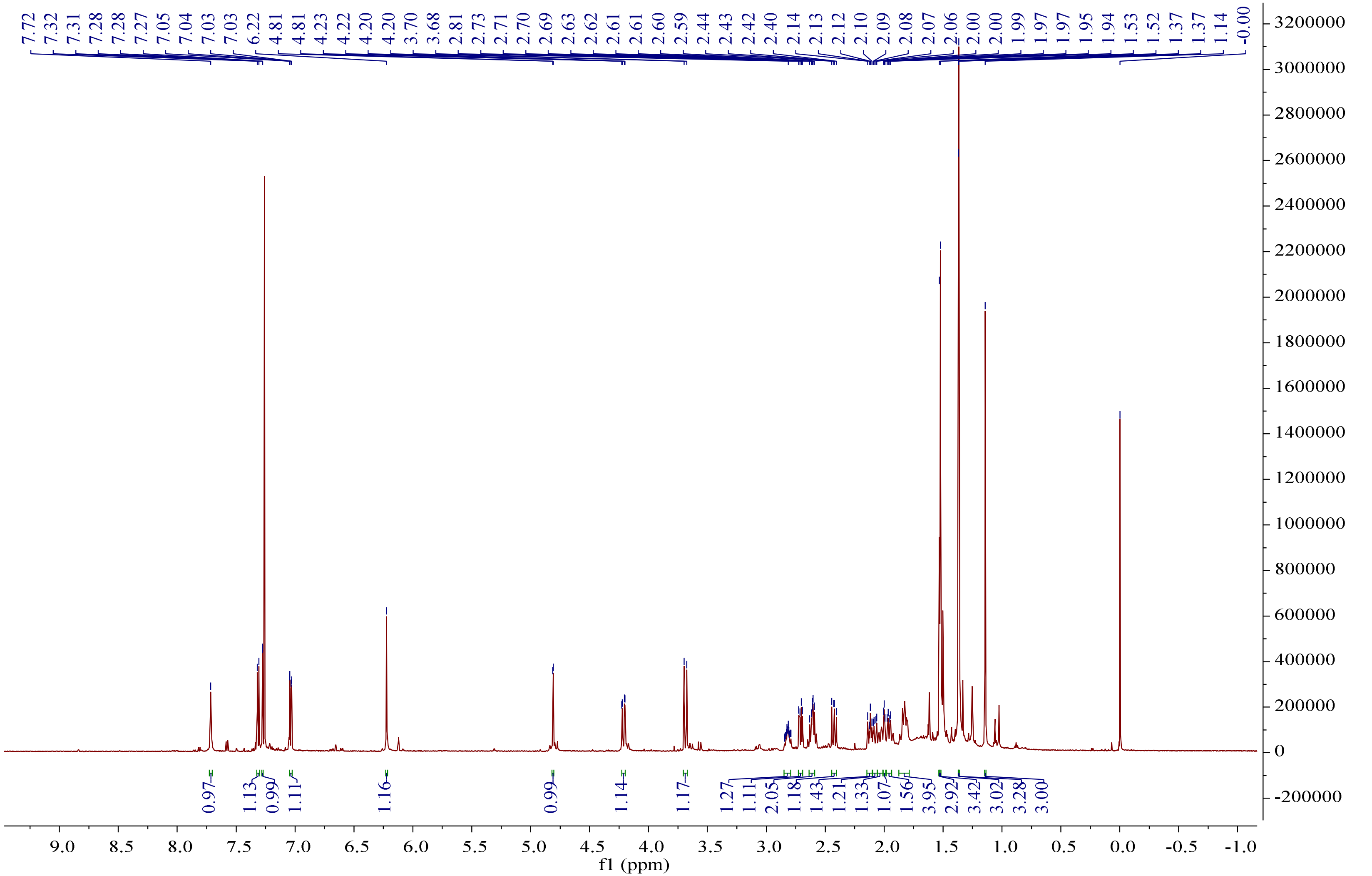

Figure S65. ^{1}H-NMR spectrum of **23** in $CDCl_3$ at 600 MHz

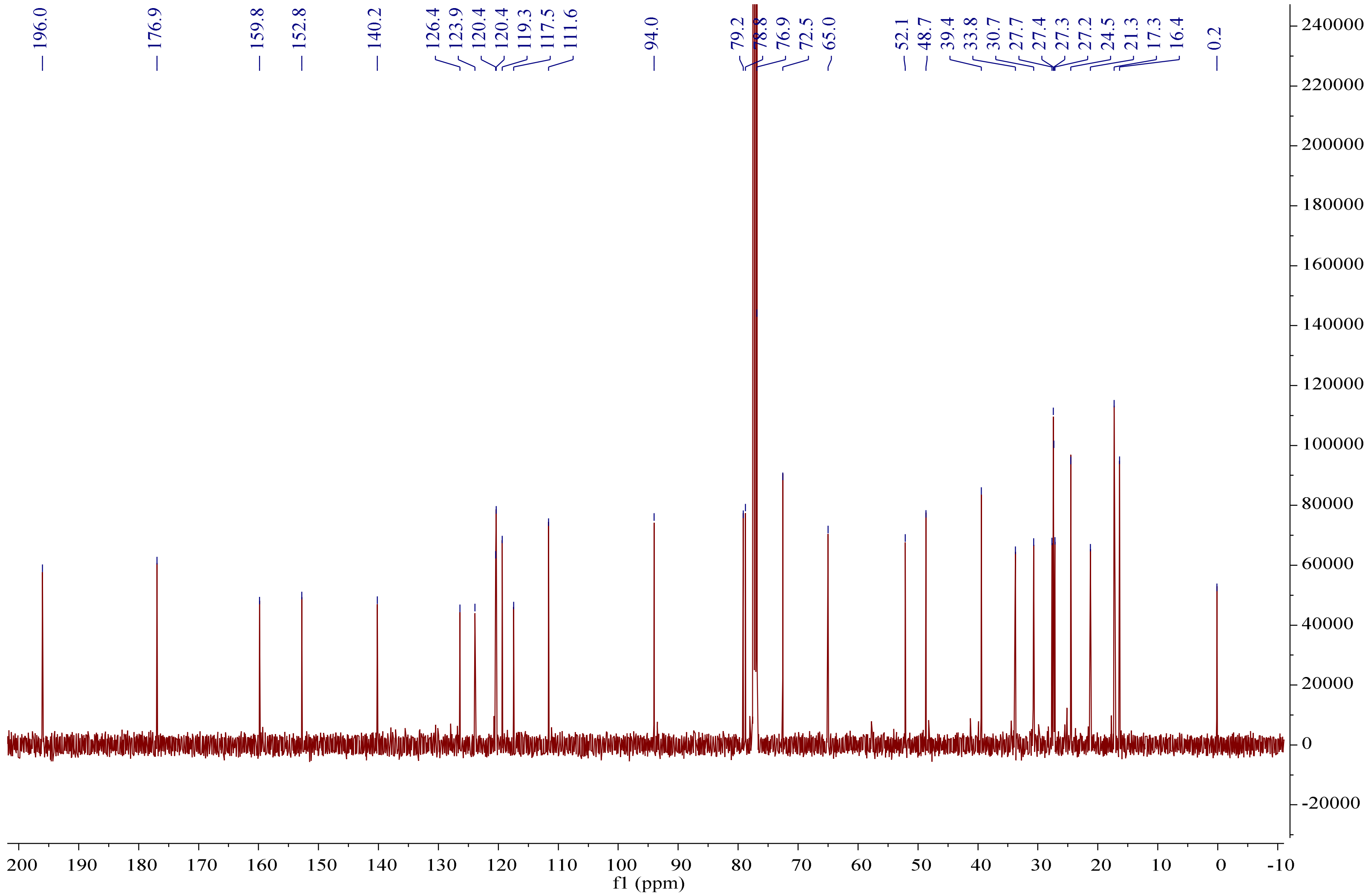

Figure S66. ^{13}C-NMR spectrum of **23** in $CDCl_3$ at 125 MHz

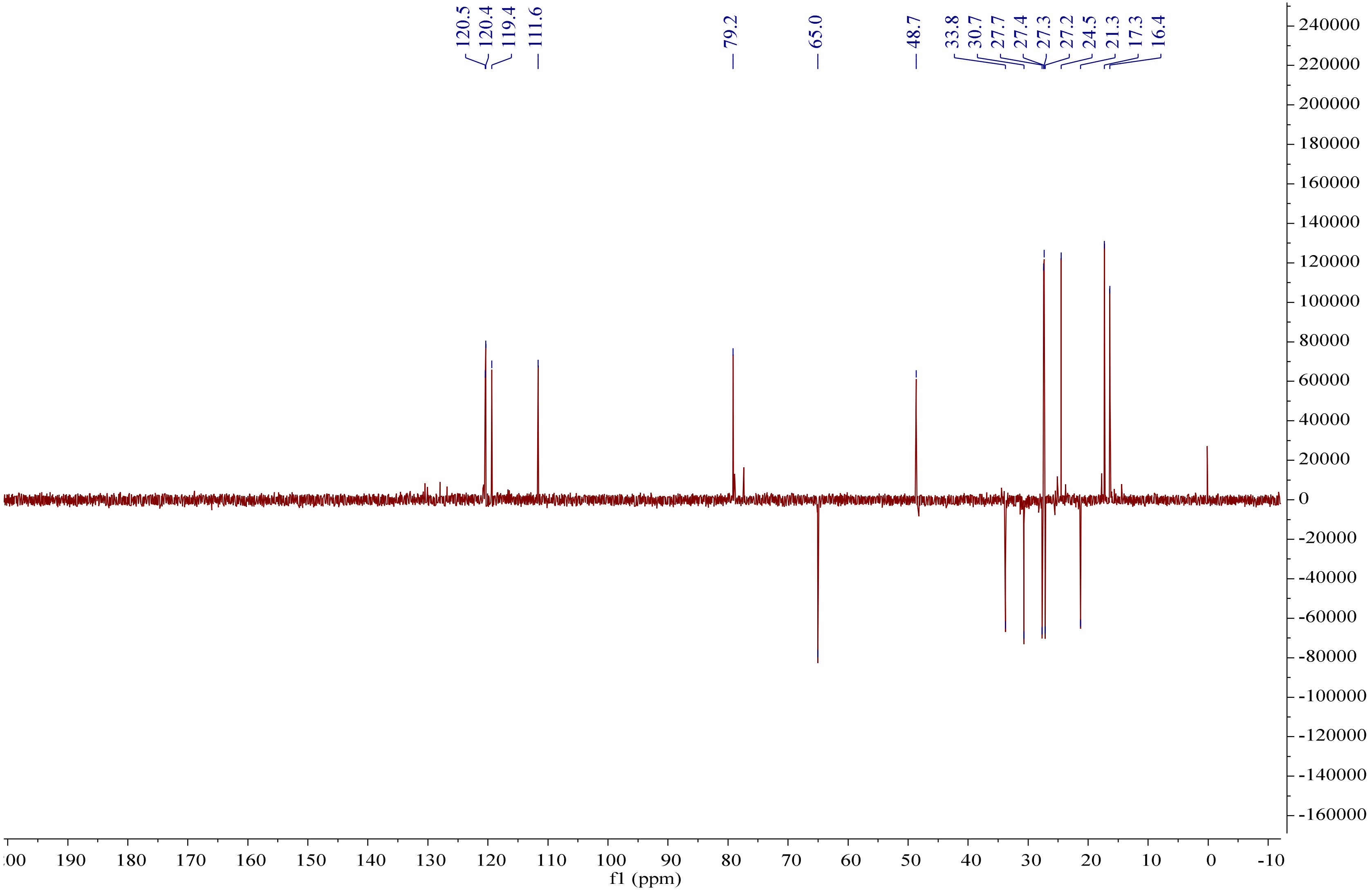

Figure S67. DEPT spectrum of **23** in $CDCl_3$ at 125 MHz

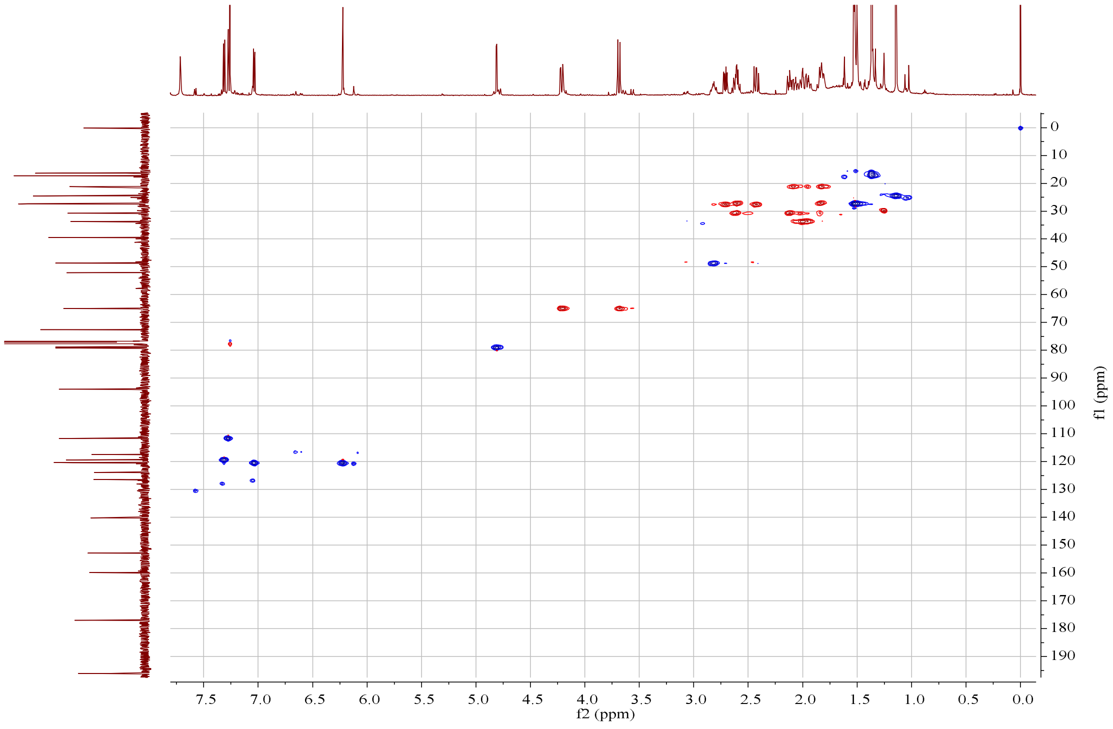

Figure S68. HSQC spectrum of **23** in $CDCl_3$

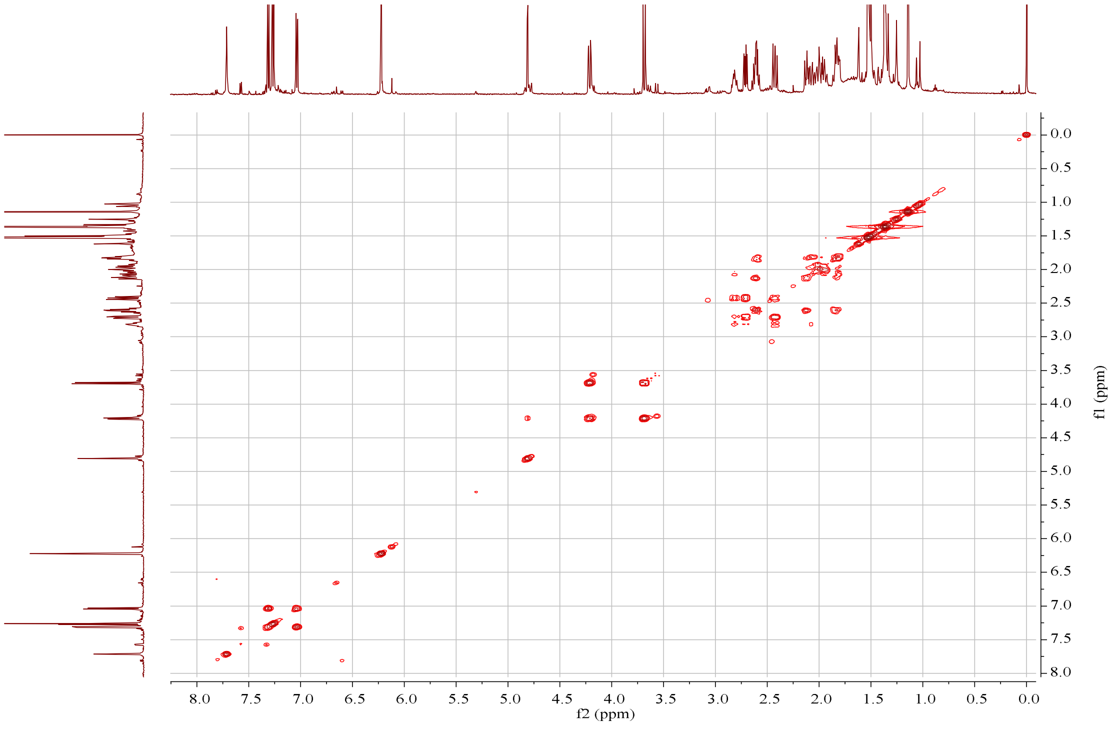

Figure S69. COSY spectrum of **23** in $CDCl_3$

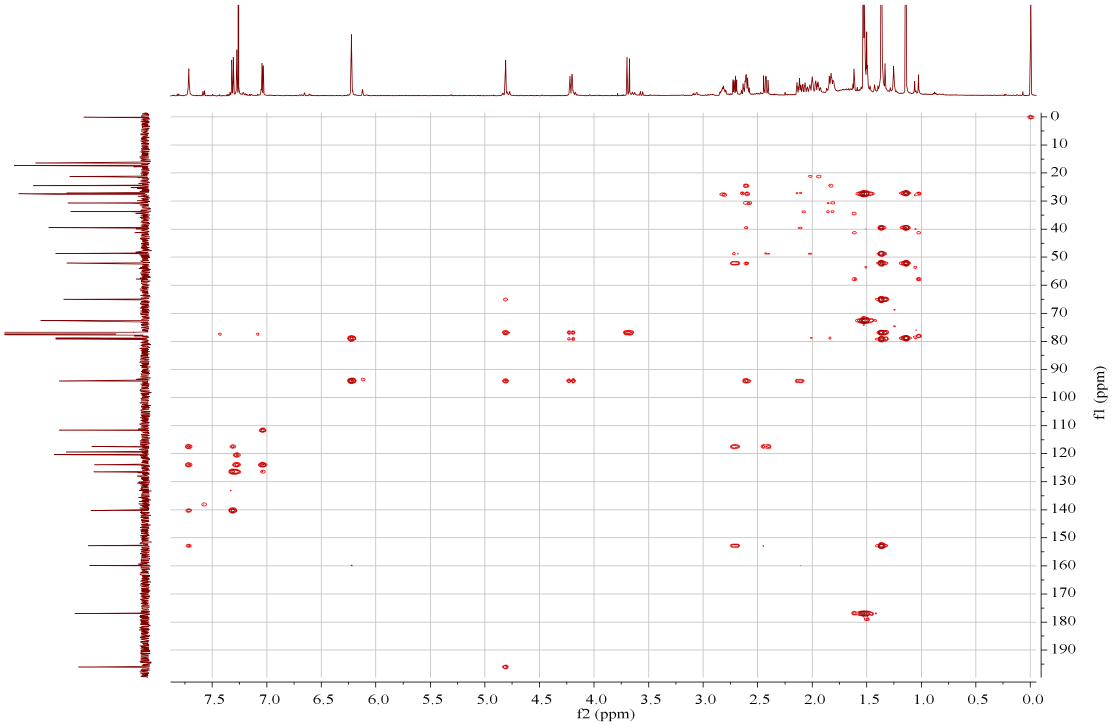

Figure S70. HMBC spectrum of **23** in $CDCl_3$

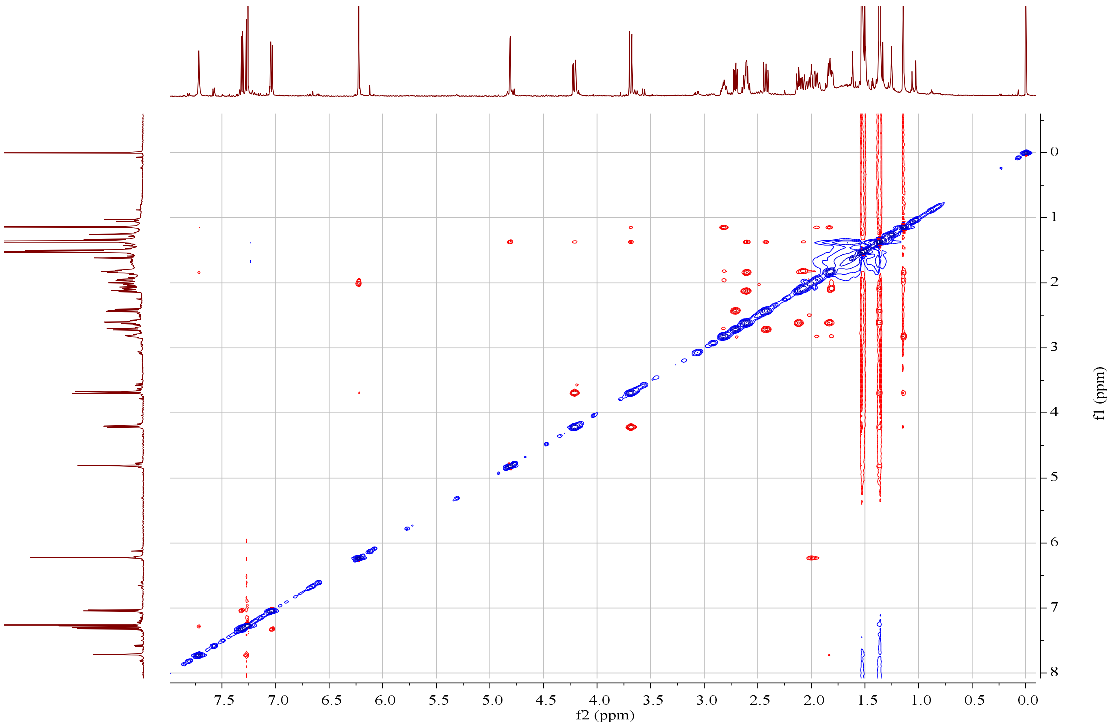

Figure S71. NOESY spectrum of **23** in $CDCl_3$

Spectrum

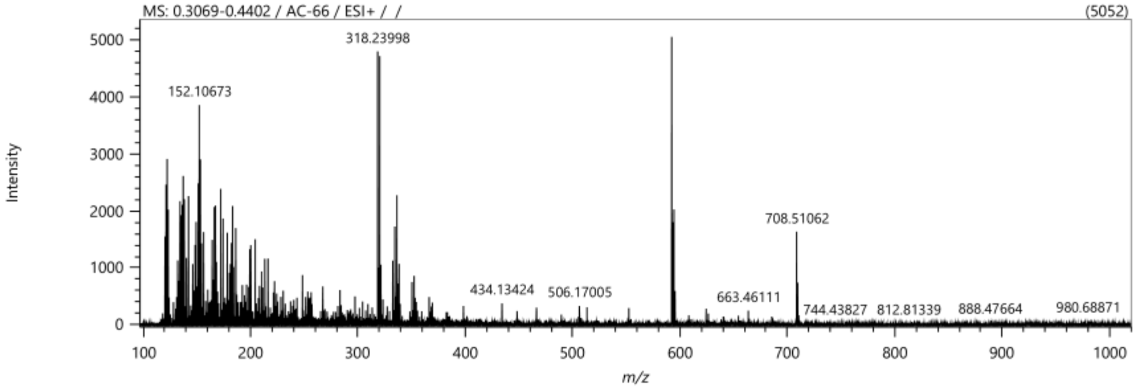

Elemental Composition

Parameters		Elements Set 1:										
Tolerance:	±5.00 ppm	Symbol	C	H	N	O	Na	S	Cl	Br	F	Si
Electron:	Odd/Even	Min	0	0	0	0	1	0	1	0	0	0
Charge:	+1	Max	200	120	3	8	1	0	1	0	0	0
DBE:	-1.5 - 200.0											

Results

Mass	Intensity	Intensity [%]	Formula	Calculated Mass	Mass Difference [mDa]	Mass Difference [ppm]	DBE
592.20681	5051.93	100.00	C31 H36 N O7 Na Cl	592.20725	-0.44	-0.74	13.5

Figure S72. MS of **24**

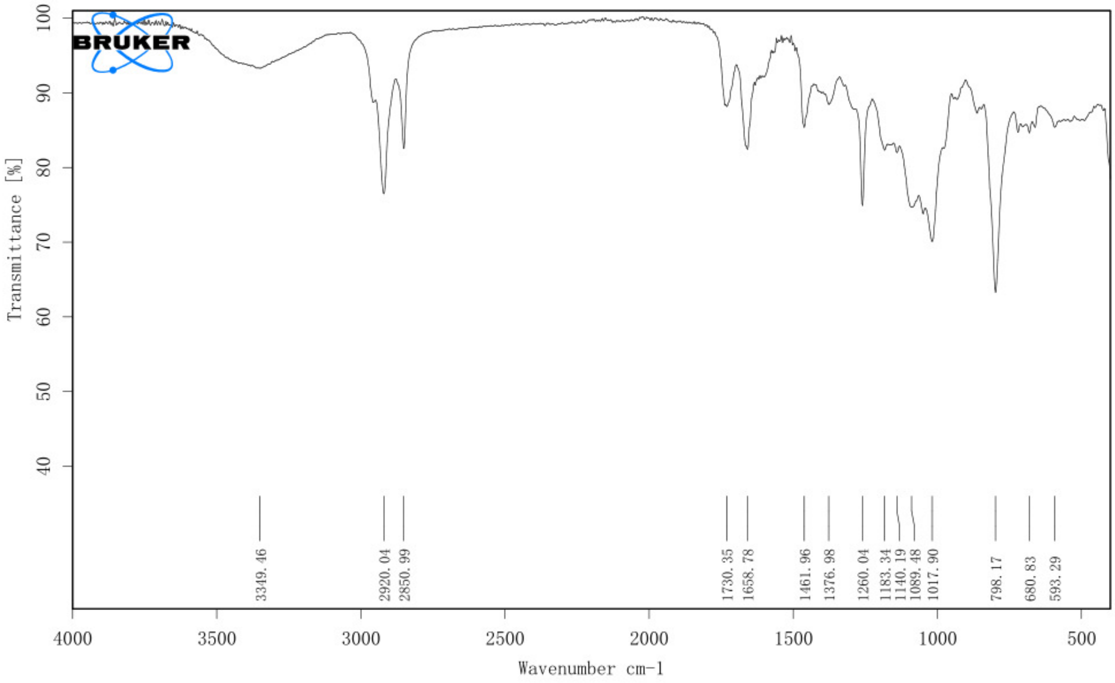

Figure S73. IR (film) of **24**

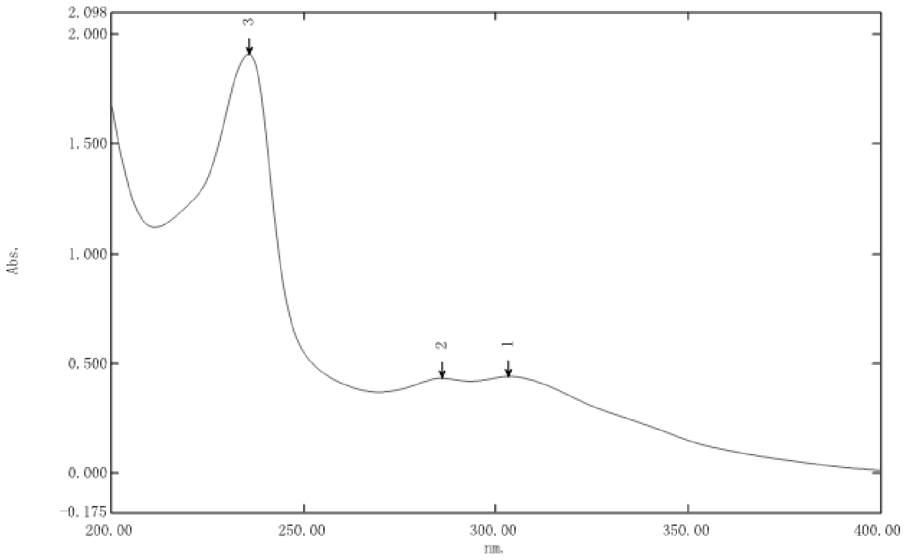

No.	P/V	波长(nm)	Abs.	描述
1		303.40	0.442	
2		286.20	0.434	
3		235.80	1.909	
4		293.20	0.419	
5		270.00	0.370	
6		211.20	1.121	

Figure S74. UV of **24** in MeCN

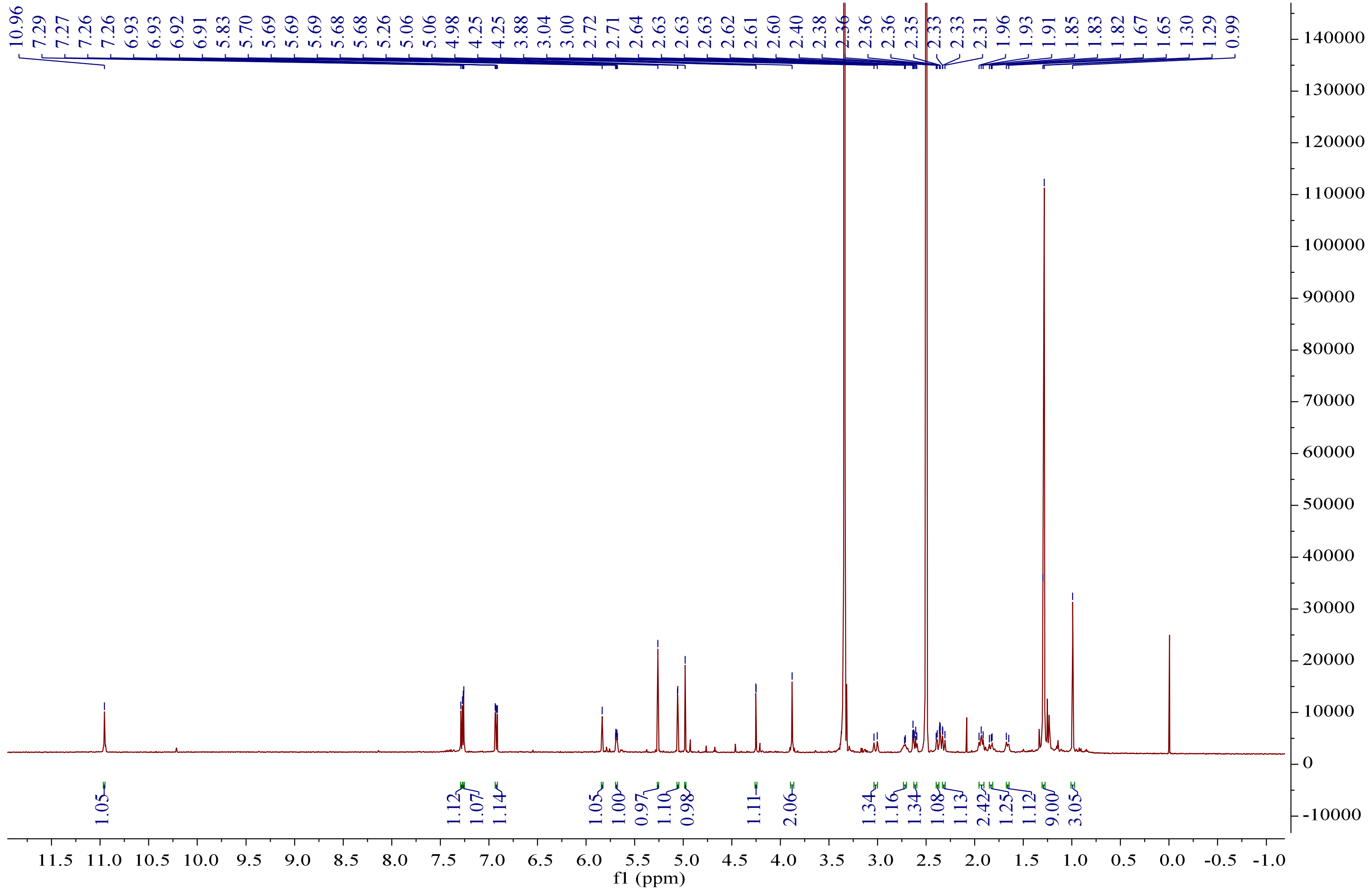

Figure S75. ^{1}H-NMR spectrum of **24** in DMSO at 500 MHz

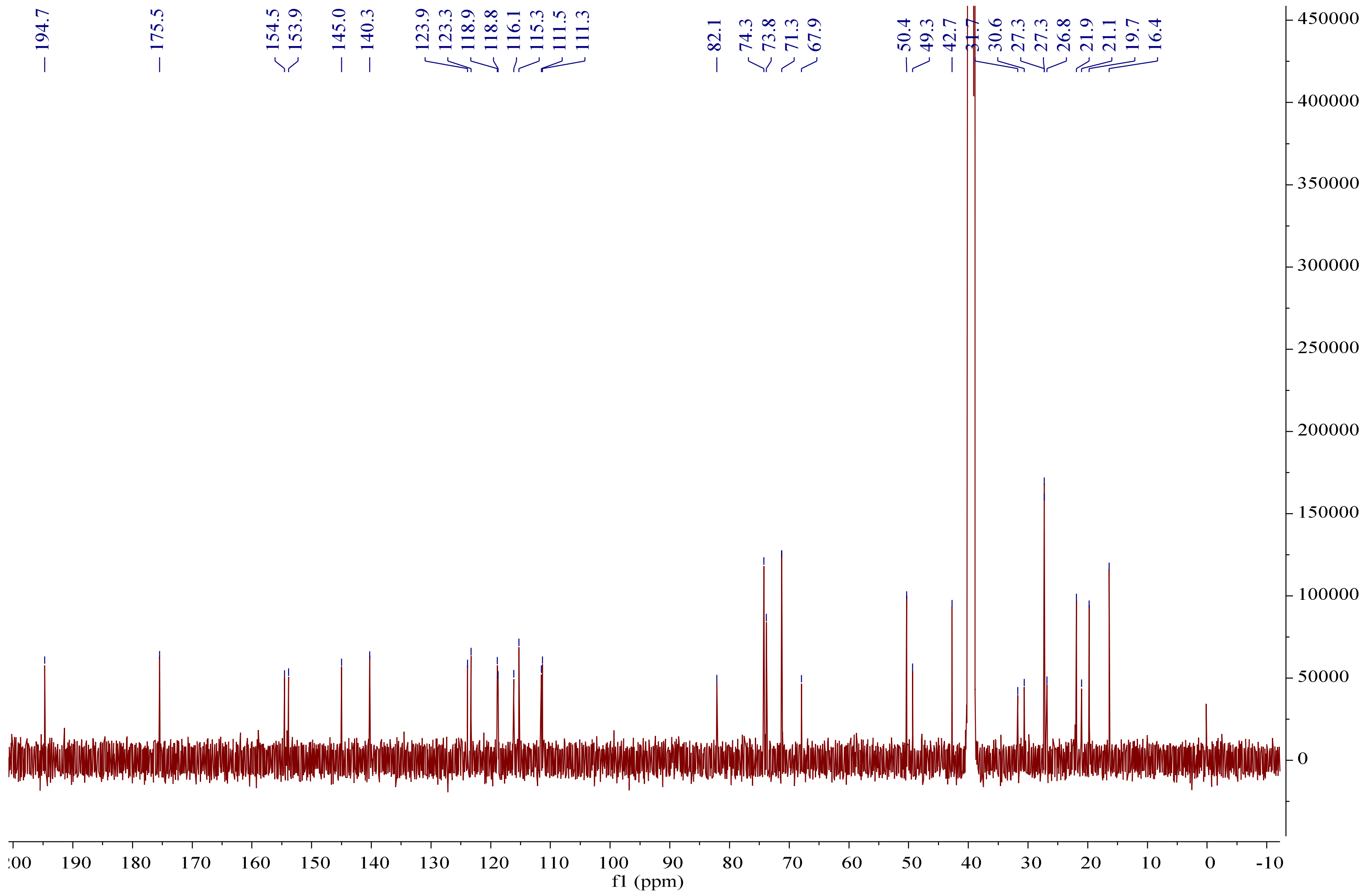

Figure S76. ^{13}C-NMR spectrum of **24** in DMSO at 125 MHz

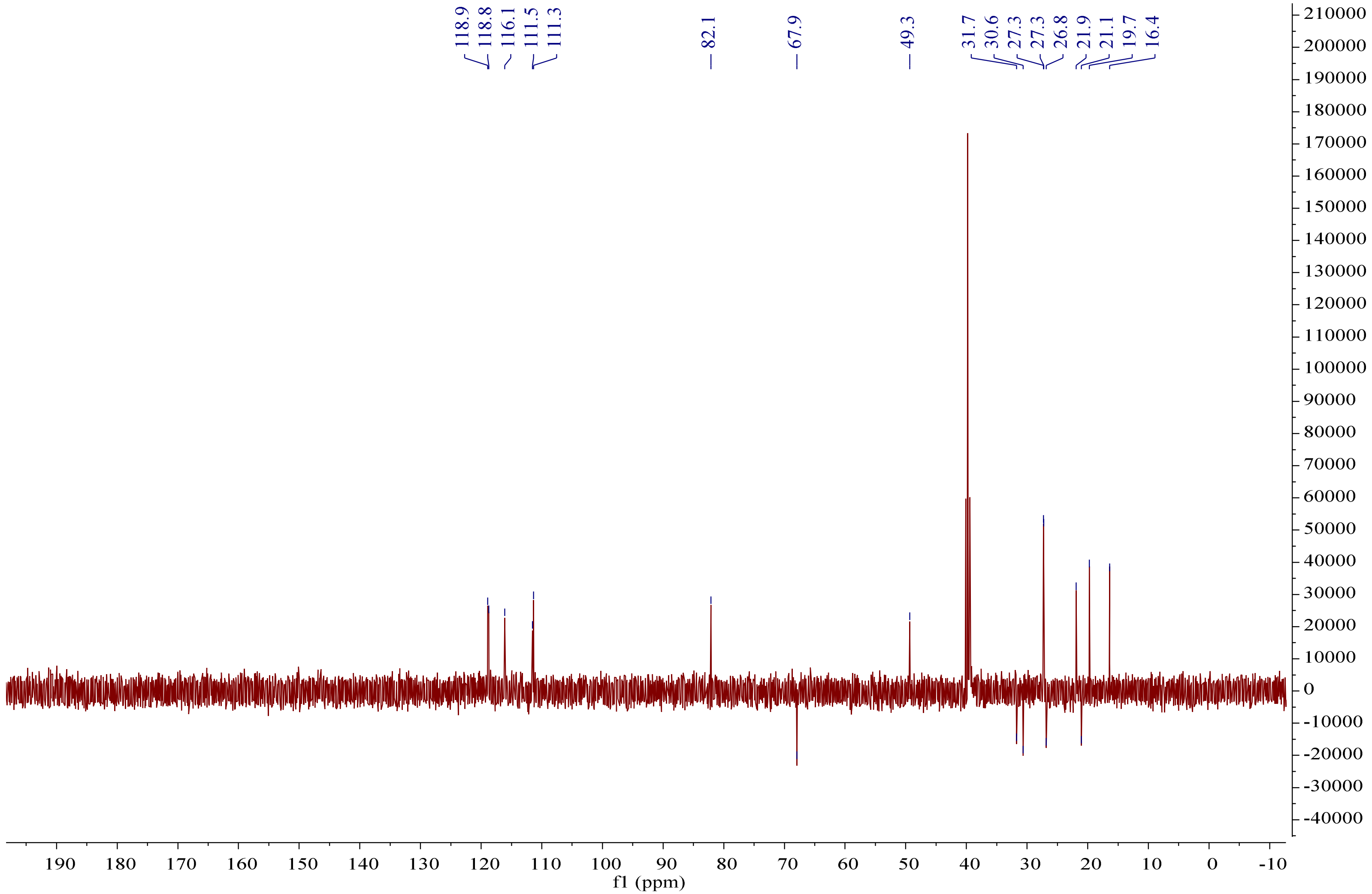

Figure S77. DEPT spectrum of **24** in DMSO at 125 MHz

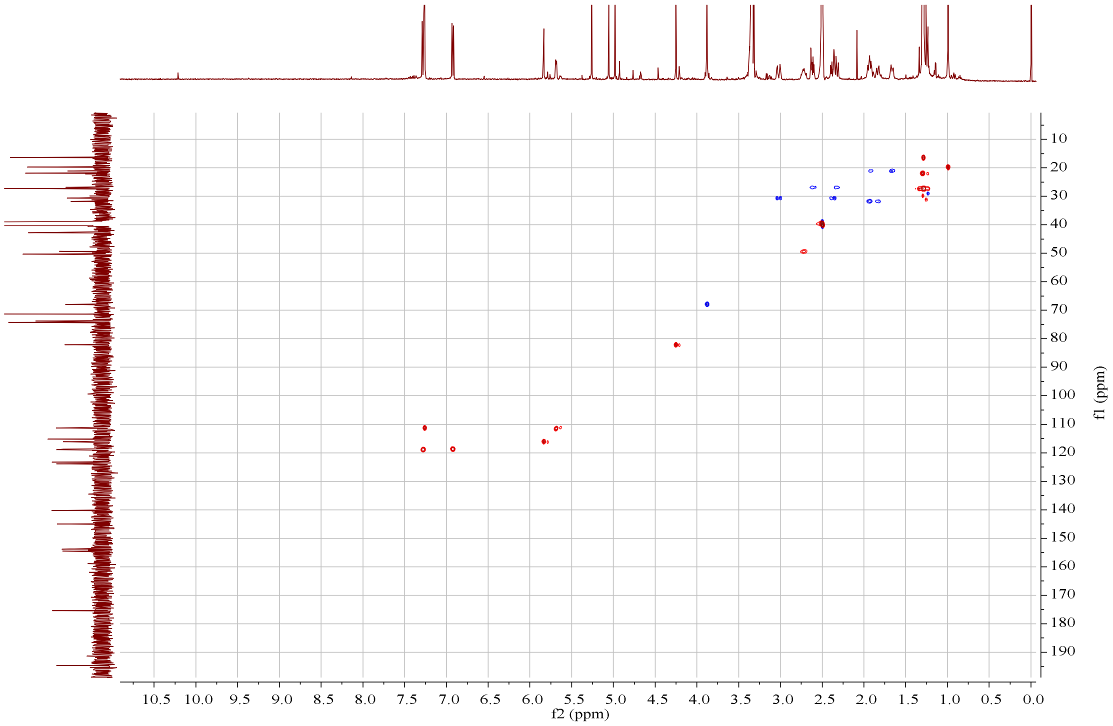

Figure S78. HSQC spectrum of **24** in DMSO

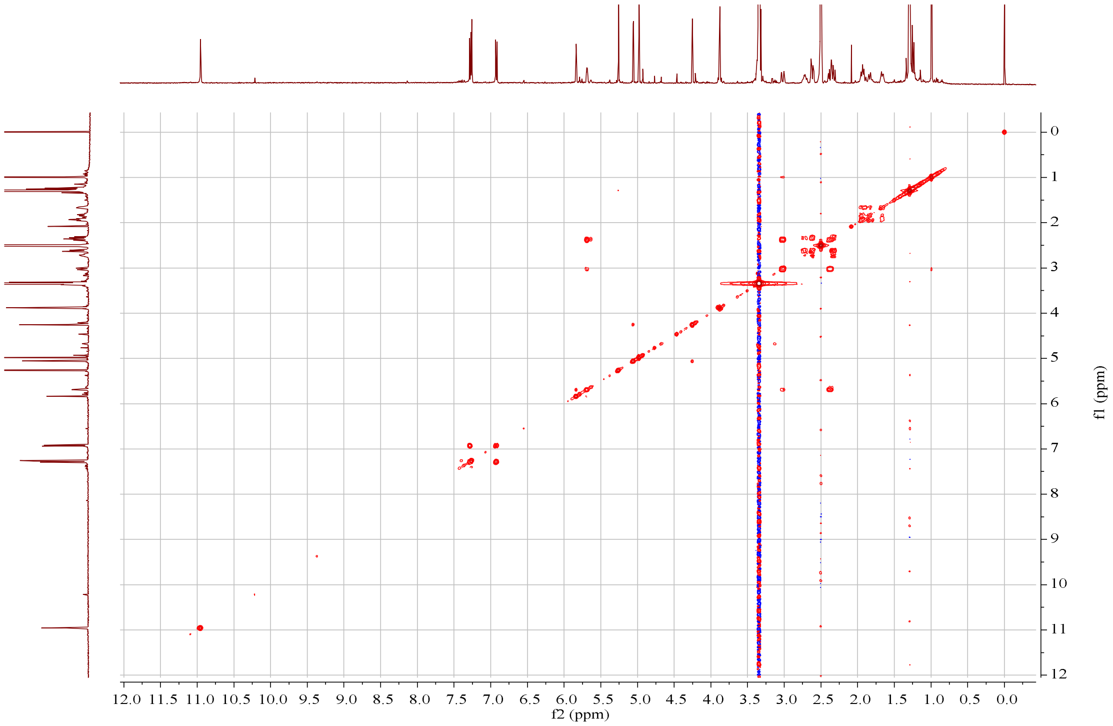

Figure S79. COSY spectrum of **24** in DMSO

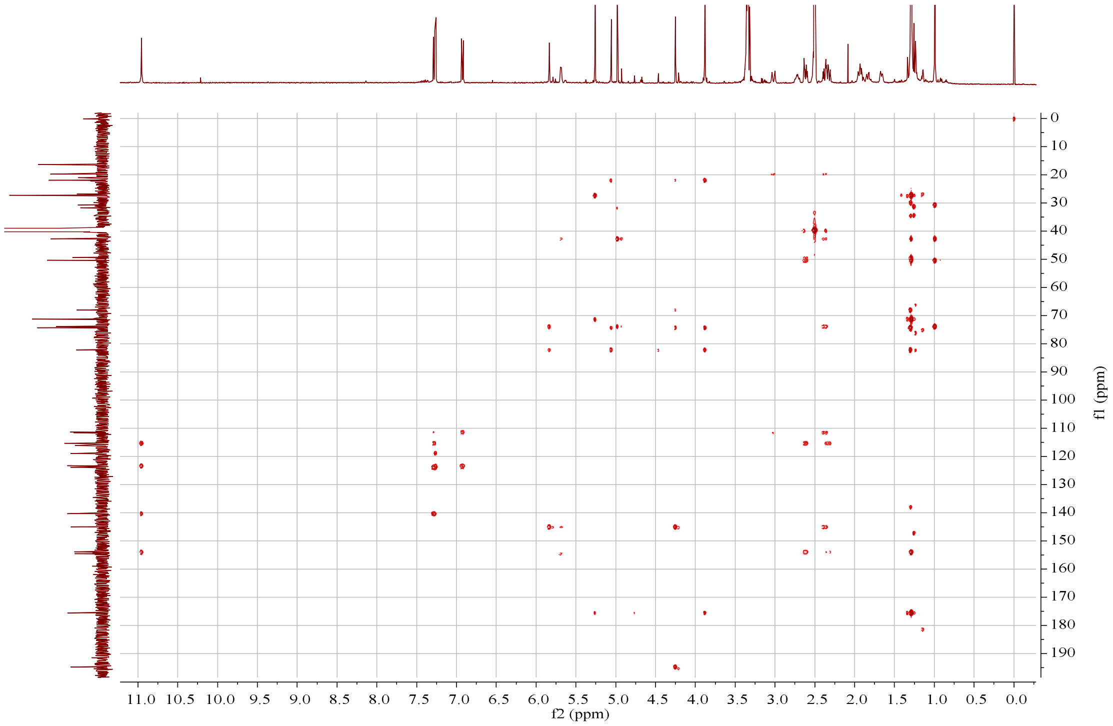

Figure S80. HMBC spectrum of **24** in DMSO

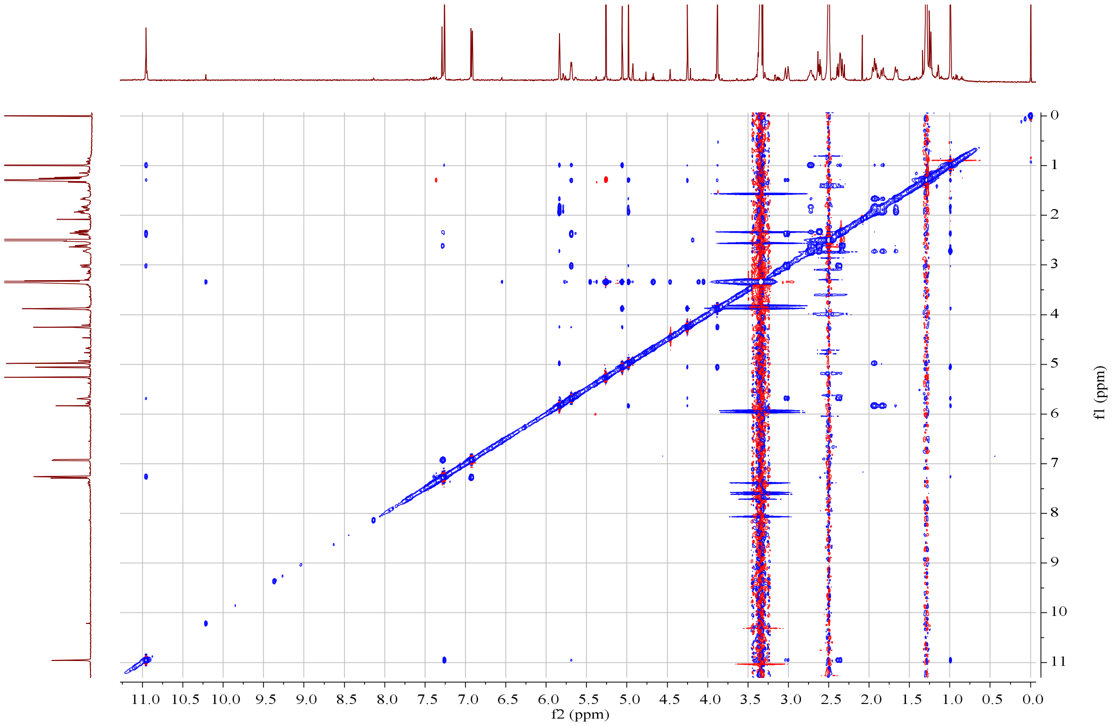

Figure S81. NOESY spectrum of **24** in DMSO

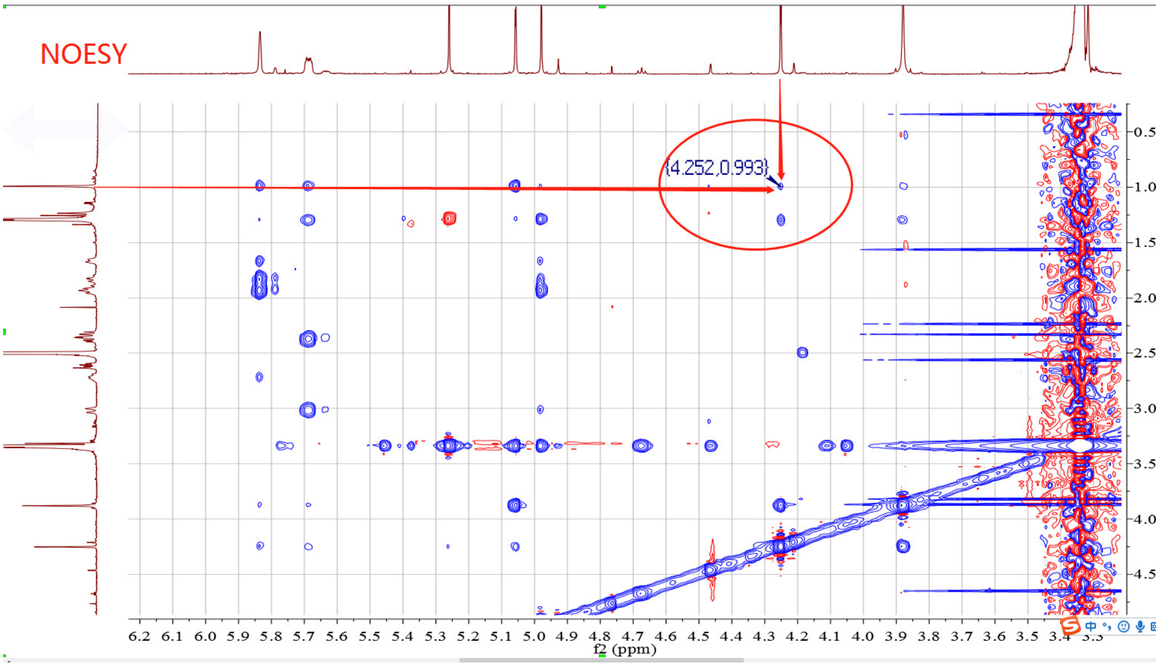

Figure S82. NOESY spectrum (enlarge) of **24** in DMSO

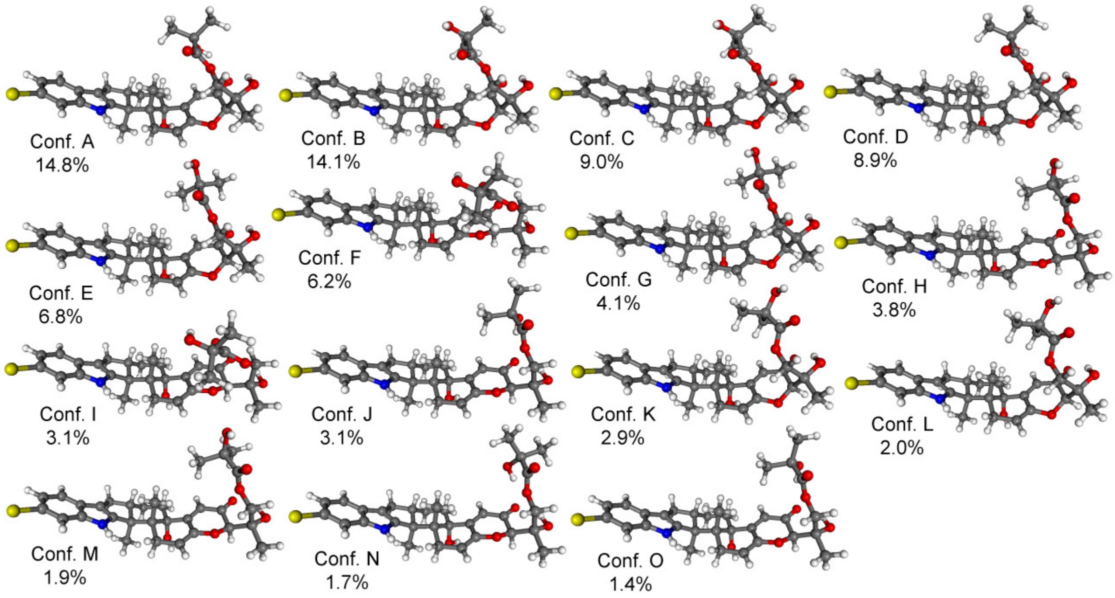

Figure S83. Structure and population of the low-energy ωB97X/TZVP PCM/MeCN conformers (> 1%) of (3*S*,4*R*,9*R*,13*S*,16*S*,27*S*)-**24**.

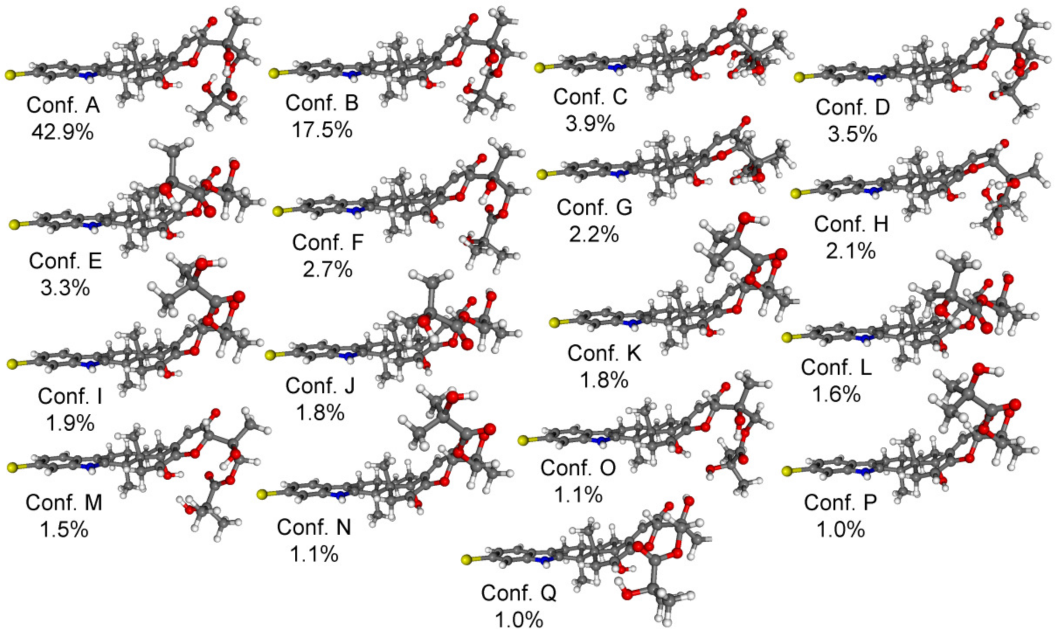

Figure S84. Structure and population of the low-energy ωB97X/TZVP PCM/MeCN conformers (> 1%) of (3*S*,4*R*,9*S*,13*S*,16*S*,27*S*)-**24**.

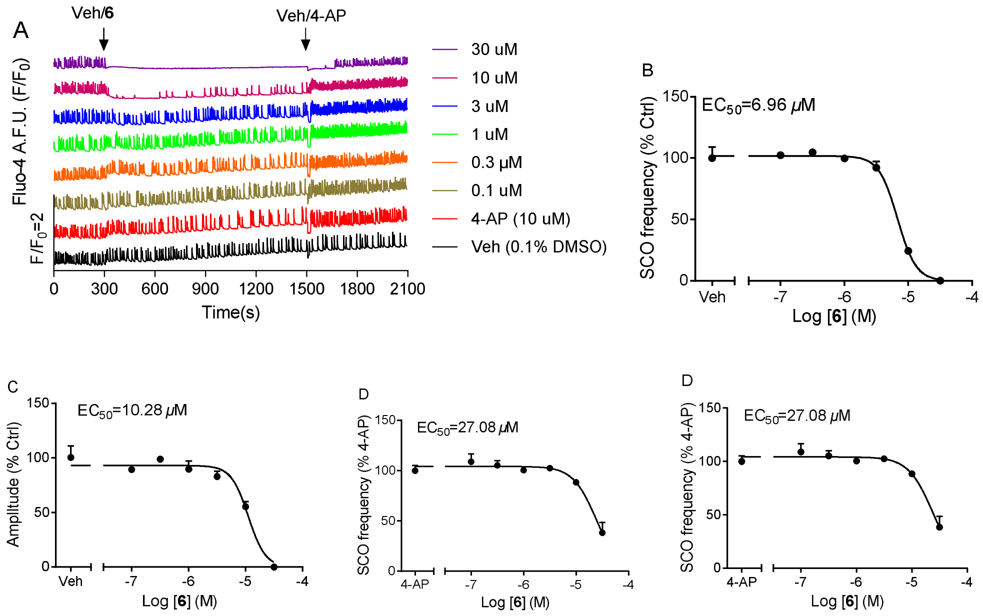

Figure S85. Effects of **6** on SCOs in primary cultured cortical neurons.

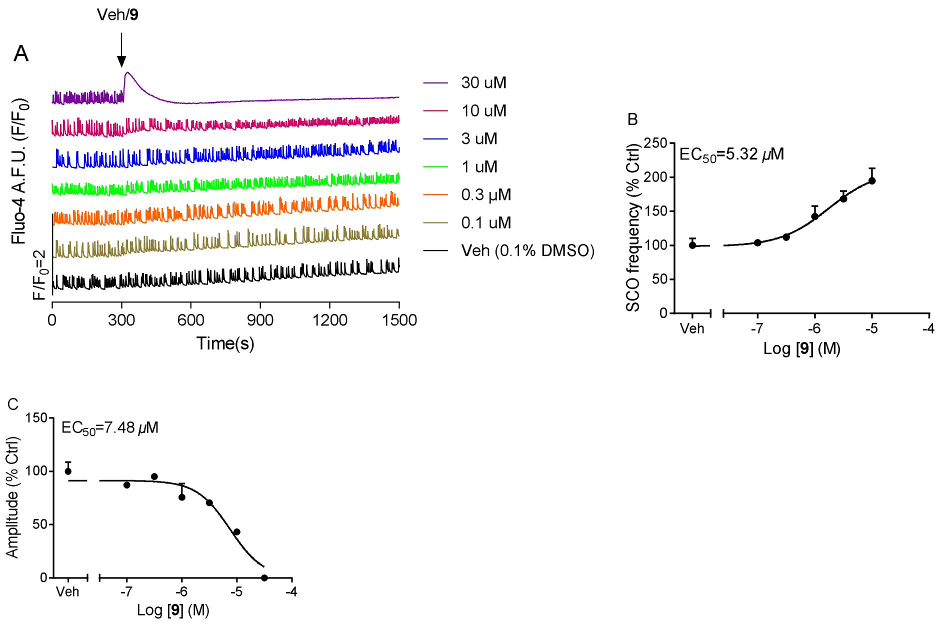

Figure S86. Effects of **9** on SCOs in primary cultured cortical neurons.

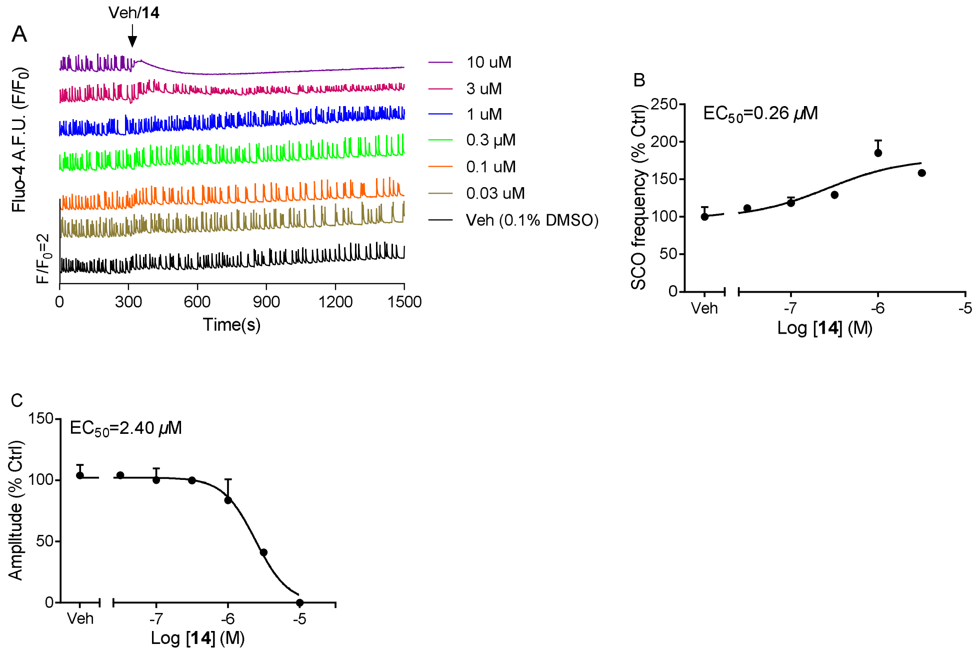

Figure S87. Effects of **14** on SCOs in primary cultured cortical neurons.

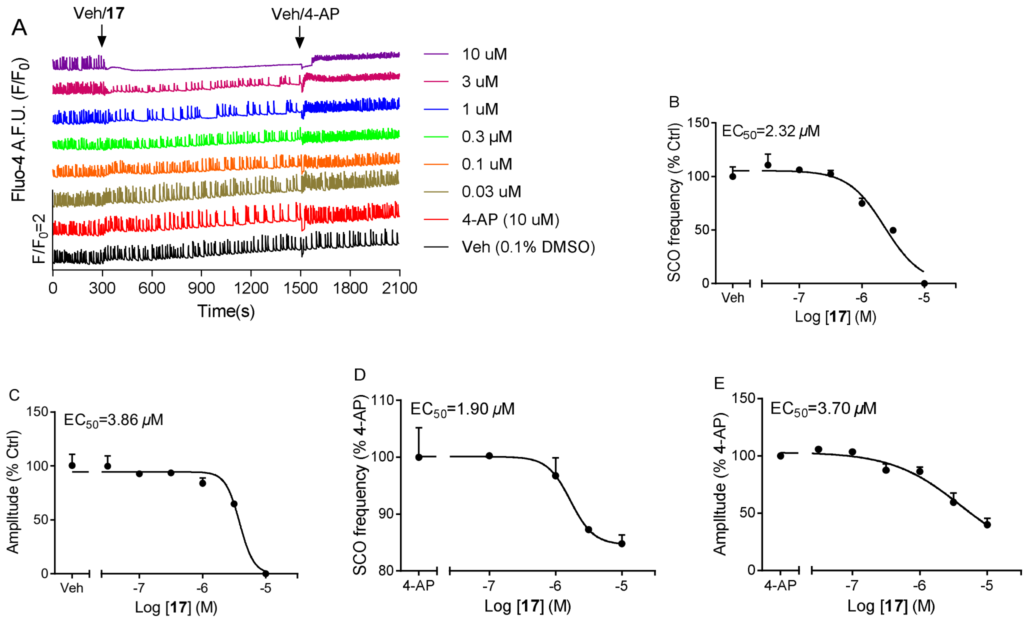

Figure S88. Effects of **17** on SCOs in primary cultured cortical neurons.

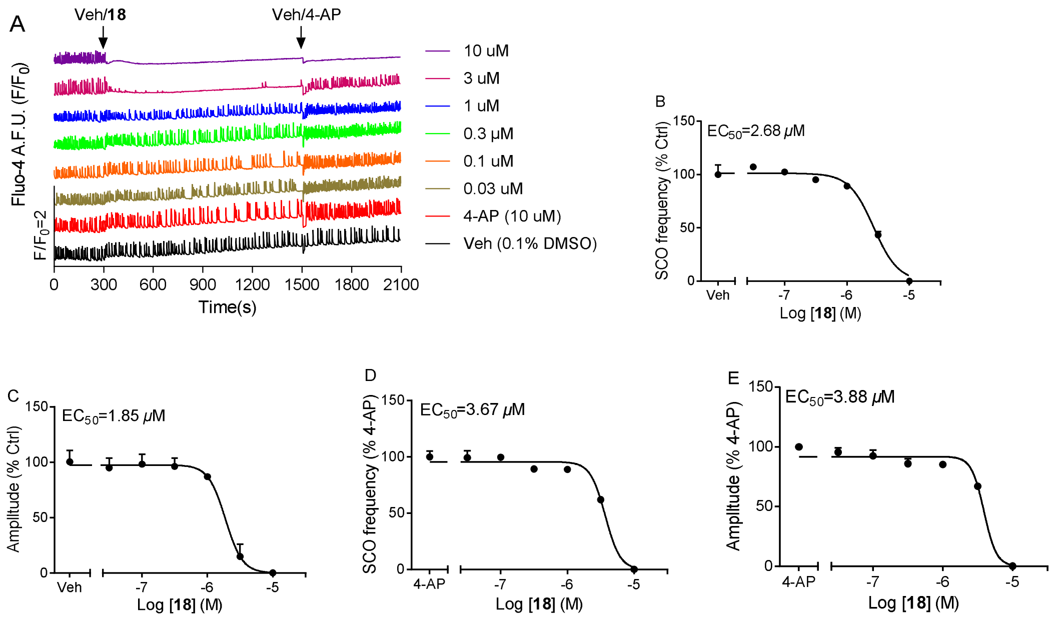

Figure S89. Effects of **18** on SCOs in primary cultured cortical neurons.

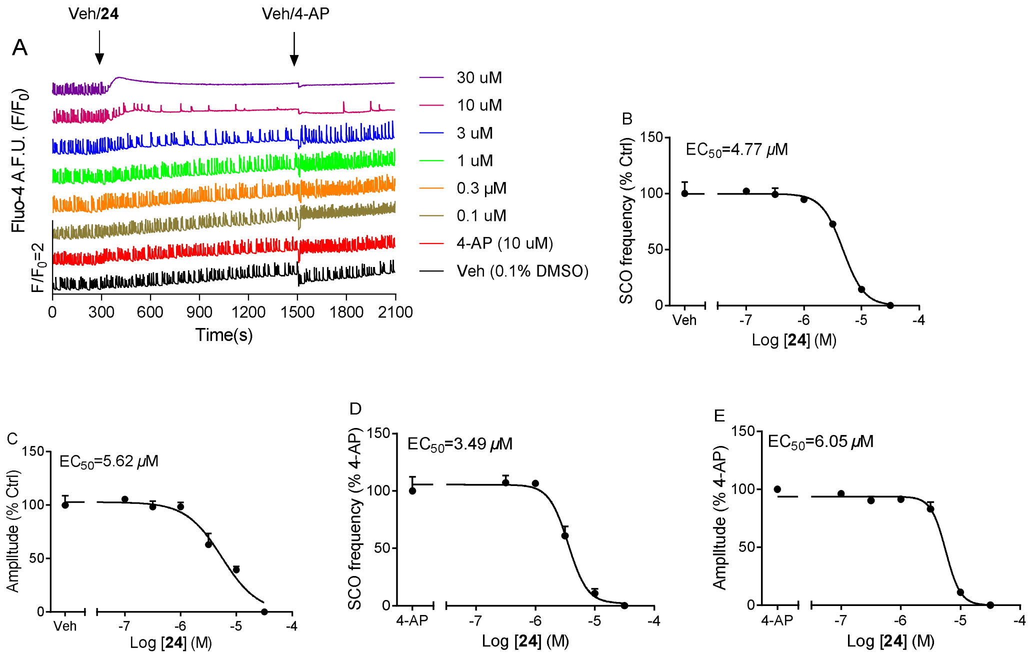

Figure S90. Effects of **24** on SCOs in primary cultured cortical neurons.

(A) Representative traces of compound suppression of SCOs before and after addition of Veh/4-AP to the cortical neurons. (B, C) Concentration-response relationships of compounds take effect on frequency and amplitude of SCOs. (D, E) Concentration–response relationships of compounds take effect on of 4-AP induced the frequency and amplitude of SCOs.
